# 2-(2-Phenylethyl)chromones increase in *Aquilaria sinensis* with the formation of agarwood

**DOI:** 10.3389/fpls.2024.1437105

**Published:** 2024-07-11

**Authors:** Yuanyuan Sun, Meiran Wang, Meng Yu, Jian Feng, Jianhe Wei, Yangyang Liu

**Affiliations:** ^1^ Key Laboratory of Bioactive Substances and Resources Utilization of Chinese Herbal Medicine, Ministry of Education and National Engineering Laboratory for Breeding of Endangered Medicinal Materials, Institute of Medicinal Plant Development, Chinese Academy of Medical Sciences and Peking Union Medical College, Beijing, China; ^2^ Key Laboratory of State Administration of Traditional Chinese Medicine for Agarwood Sustainable Utilization and Hainan Provincial Key Laboratory of Resources Conservation and Development of Southern Medicine and International Joint Research Center for Quality of Traditional Chinese Medicine, Hainan Branch of the Institute of Medicinal Plant Development, Chinese Academy of Medical Sciences and Peking Union Medical College, Haikou, China

**Keywords:** *Aquilaria sinensis*, agarwood, accumulation, whole-tree agarwood-inducing technique, non-targeted metabolomics technology, 2-(2phenylethyl)chromones

## Abstract

Obtained from *Aquilaria* Lam. and *Gyrinops* Gaertn., agarwood is a prestigious perfume and medicinal material in the world. Its primary chemical constituents and indicators of agarwood's development are 2-(2-phenylethyl)chromones (PECs). However, how PECs affect its quality, accumulation, and transformation pattern is still unclear. The present study investigated this issue by monitoring resin filling in agarwood generated by the whole-tree agarwood-inducing technique over a span of a year, observing the ethanol extract concentration at different sampling times, and statistically examining PECs in agarwood from each sampling period. In agarwood, the resin accumulated over time, except during the 4th–6th month due to the creation of a barrier layer. The relative content of total PECs demonstrated an overall increase throughout the year but a decrease from the 4th month to the 6th month, and the relative content of 19 PECs that persisted throughout the year was positively correlated with the content of ethanol extracts. In addition, the process of chromone accumulation was accompanied by the production and transformation of different types of chromones, with flindersia type 2-(2-phenylethyl)chromones, epoxy-2-(2-phenylethyl)chromones, and diepoxy-2-(2-phenylethyl)chromones being the major chromone components; in addition, the content of 5,6,7,8-tetrahydro-2-(2-phenylethyl)chromones kept increasing after 6 months of agarwood formation. Three main trends were identified from 58 analogs of PECs, each with notable variation. The first type had the highest content at the beginning of resin formation. The second type had the highest content at 6 months and then started to decrease, and the third type had a slowly increasing content. As a whole, this study systematically investigated the accumulation of PECs during injury-induced agarwood production in *A. sinensis*, which is of scientific significance in resolving the transformation of PECs and revealing the secret of agarwood formation.

## 1 Introduction

Agarwood, musk, borneol, and sandalwood are four of the most famous perfumes in China. Among them, agarwood is the most valuable material, with a global trade worth tens of billions of dollars (Gao et al., 2017). Agarwood is a resinous wood obtained from *Aquilaria* Lam. and *Gyrinops* Gaertn (Li et al., 2021). In China, only *A. sinensis* (Lour.) Gilg, an endemic species, is used as a plant resource for producing agarwood (China Pharmacopoeia Committee, 2020). Agarwood has been extensively used in religious ceremonies, fragrances, and artworks, such as wood carving (Li et al., 2021). Moreover, it is also employed in traditional Chinese medicinal drugs, and many ancient books have recorded it. For example, *Ben Cao Yan Yi*, written in the Song Dynasty, states that *A. sinensis* is very widespread and extremely abundant in the wild, especially in areas close to the sea. However, wild *A. sinensis* is almost extinct at present due to excessive exploitation, and it has been protected as an endangered wild plant (CITES, http://checklist.cites.org).

In addition to overexploitation, the special formation mechanism of agarwood and the long formation time also contribute to the scarcity of wild agarwood. To obtain more agarwood to meet market demand, the formation mechanism as well as artificial induction techniques have been explored. Zhang et al. (2010) proposed the hypothesis that the defense response of *A. sinensis* induces agarwood formation. In other words, physical or chemical damage or fungal infestation can trigger a defensive reaction in *A. sinensis*, producing defensive chemicals that involve PECs and sesquiterpenes, which combine with other components to form fillers that block the ducts of the secondary xylem and accumulate over time; hence, the agarwood forms slowly. Based on this hypothesis, Liu et al. (2013) invented a stable and efficient Agar-Wit. They injected the inducer into *A. sinensis*, and the plant infusion tissue transported the inducer to different tissues of the tree, activating the defense reaction of the plant’s thin-walled cells to generate secondary metabolites, such as PECs and sesquiterpenes, which eventually led to agarwood production throughout the body of *A. sinensis* from the trunk to the branches after a long accumulation time (Mei et al., 2013). Furthermore, this hypothesis was confirmed by Gao and Wei (2016), who initially discovered the process of agarwood synthesis.

It has been well established that external damage can induce the generation of two characteristic components of *A. sinensis*, sesquiterpenes, and PECs, which fill the xylem to promote agarwood formation (Ito et al., 2005; Kumeta and Ito, 2010; Naef, 2011). In recent years, scholars in China (Gao Z. H. et al., 2012; Xu et al., 2013, 2016), Japan (Kumeta and Ito, 2016), and Vietnam (Nguyen et al., 2017) have begun to focus on the deep molecular mechanisms of agarwood formation and how to efficiently regulate and improve its quality. The molecular mechanism of sesquiterpene production, the primary constituents of resin, and the molecular network that causes the thickening of the agarwood layer have been largely revealed (Gao and Wei, 2016). In contrast, studies on the biosynthetic pathways and metabolic regulation of PECs are limited.

The PECs are characteristic components of agarwood and contain a myriad of biological functions, such as antiviral, antidiabetic, anti-inflammatory, antibacterial, and antioxidant effects (Yu et al., 2022). Primarily, PECs are structurally composed of monomers, dimers, trimers, and others; monomers include 5,6,7,8-tetrahydro-2-(2-phenylethyl)chromones (THPECs), diepoxy-2-(2-phenylethyl)chromones (DEPECs), epoxy-2-(2-phenylethyl)chromones (EPECs), and flindersia type 2-(2-phenylethyl)chromones (FTPECs) (Okudera and Ito, 2009; Naef, 2011; Gao et al., 2019). In addition, on the B ring, there are four main types of substitutions: (i) no substitutions; (ii) OH substitutions; (iii) OCH3 substitutions; and (iv) OH and OCH3 replacements.

To investigate the formation of PECs after fungal infection of *A. sinensis*, Qi et al. (2000) established a method for the determination of 6,7-dimethyl-PECs and 6-methoxy-2-[2-(4′-methoxy)phenethyl]chromones by high-performance liquid chromatography (HPLC) and analyzed samples from healthy *A. sinensis* infected with *Menanotus flavovirens* for different lengths of time. The PECs began to form in the 3rd month onward, and as time went on, their content grew. Yagura et al. (2005) also hypothesized that ternary oxephenone plays a significant function in the early synthesis of PECs. Yang et al. (2016) analyzed the chemical composition of agarwood at 2, 4, and 5 years of age using HPLC-DAD-ESI-MS/MS. They discovered that as the agarwood formation time was prolonged, the total relative content of ternary-oxetane-PECs tended to decrease, whereas the relative content of tetrahydrochromones and simple PECs tended to increase. Liao et al. (2018) sampled and analyzed agarwood at 1-month intervals for 1 year and found that simple PECs were the most considerable chromones in agarwood and that PECs with no substituent on the B ring and 4′-methoxy were distributed throughout the agarwood formation time, whereas PECs with both 3′-hydroxyl substitution and 4′-methoxy substitution on the B ring appeared only at the 5th month after agarwood formation. The reciprocal conversion and accumulation of PECs occur concurrently with the development of agarwood; thus, the length of the agarwood formation time is one of the reasons for the formation of PECs with various structures. The age of *A*. *sinensis* may also be an influencing factor (Liu et al., 2013; Kalita and Lekhak, 2015), but this has not yet been reported. The PECs are not simply superimposed and accumulated during the agarwood formation process. There may be a conversion between them, but the mechanism of their accumulation remains unknown.

Research on the buildup of PECs can help to clarify the relationship between PECs and quality during the agarwood production process. This will assist in fully explaining the mechanism of agarwood formation and, consequently, improve artificial induction technology, regulating and improving agarwood quality. For these reasons, this study analyzed the cumulative changes in PECs during the agarwood formation process at both macro and micro levels. First, a freezing microtome was used to observe the cross-sectional morphology and color of the inter-xylary phloem in the main places where agarwood resin was formed and accumulated, and changes in microscopic characteristics were observed over time. Second, based on the resin extract, the content of the ethanol extract was analyzed. Third, UNIFY was used to construct a library of PECs, and combined with fingerprint maps, the differences in PECs among agarwood samples were analyzed and identified. Fourth, based on UPLC-Q/TOF-MS, non-targeted metabolomics analysis was carried out to analyze the different metabolites in samples from different sampling times, and then their mutual transformation relationship was analyzed. This work will help with future industrial development and agarwood resource utilization in addition to offering fresh insights into the distinctive buildup of PECs.

## 2 Materials and methods

### 2.1 Plant materials

*A. sinensis* 3 years of age and with a diameter at breast height of approximately 5 cm was selected as the material for the agarwood induction experiments at the plantation in Huazhou, Guangdong, China. Agarwood samples produced by Agar-Wit have been collected 2, 4, 6, 8, 10, and 12 months after injection with an agarwood inducer (Liu et al., 2013). In addition, the samples collected immediately after the infusion were chosen as the model control group (i.e., the JXY group), and non-infused healthy *A. sinensis* samples were chosen as the blank control group (BM); the remaining details are shown in **Table 1**. Sampling and pretreatment were performed according to the Hainan standard of China DB46/T 257–2013 (https://dbba.sacinfo.org.cn/).

**Table 1:** Grouping of plant materials.

Group	Description	Sample no.	Group	Description	Sample no.
Black control group (BM)	Uninfused healthy A. sinensis, collected immediately	BM1	6-month group for agarwood formation (6M)	Collected after 6 months of infusion	6M-025
		BM2			6M-031
		BM3			6M-032
		BM4			6M-033
		BM5			6M-034
		BM6			6M-036
Model control group (JXY)	The samples taken immediately after the infusion	JXY1	8-month group for agarwood formation (8M)	Collected after 8 months of infusion	8M-035
		JXY2			8M-043
		JXY3			8M-044
		JXY4			8M-045
		JXY5			8M-046
		JXY6			8M-141
2-Month group for agarwood formation (2M)	Collected after 2 months of infusion	2M-011	10-Month group for agarwood formation (10M)	Collected after 10 months of infusion	10M-051
		2M-012			10M-052
		2M-013			10M-053
		2M-015			10M-054
		2M-016			10M-055
		2M-143			10M-056
4-Month group for agarwood formation (4M)	Collected after 4 months of infusion	4M-014	12-Month group for agarwood formation (12M)	Collected after 12 months of infusion	12M-042
		4M-021			12M-061
		4M-022			12M-062
		4M-023			12M-063
		4M-024			12M-064
		4M-026			12M-065

### 2.2 Microstructural characteristics

Agarwood samples (approximately 0.5 cm in thickness) were collected and subjected to a 5-h heating process at 70°C using a water bath to soften them. Then, they were dried and trimmed into squares of the right size for slicing with a CM1950 freezing microtome (Leica Corporation, Wetzlar, Hesse, Germany). The slice thickness was 60 μm. The slices were soaked in a permeabilizing solution for 8 h, and slide specimens were made to observe the cross-sectional morphology and color of inter-xylary phloem where the resin initially formed and mainly accumulated. The objective magnification was 4×, 10×, and 20×.

### 2.3 UPLC-Q/TOF-MS/MS analysis

To optimize the extraction method of chromones in agarwood samples, the six peaks with the highest content in the HPLC chromatogram of the extract were used as indexes for investigation, comparing the ultrasonic-assisted extraction method and the hot reflux extraction method and examining methanolic and ethanolic aqueous solutions at different concentrations. Based on the experimental results (**Supplementary Figure S1**), the hot reflux method for chromone extraction with 50% ethanol was used as the extraction solvent. The agarwood sample of precisely 0.2 g was added to 20 mL of 50% ethanol, which was then tightly corked and reweighed before being refluxed at 90°C for a duration of 60 min. After cooling, the sample was reweighed again, with any weight loss being compensated by adding an appropriate amount of 50% ethanol. The resulting mixture was shaken well, filtered, and diluted to obtain a solution in a specific volume of 25 mL.

This experiment used a Waters ACQUITY UPLC BEH C18 column (2.1 mm × 150 mm, 1.7 μm) to separate the compounds and a Xevo G2-XS UPLC-QTOF-MS (Waters Corporation, Milford, MA, USA) to analyze the compounds in the agarwood samples. The mobile phase was acetonitrile (A) and 0.1% formic acid water (B). The column temperature was 25°C, and the flow rate was 0.1 mL/min. The elution gradient was as follows: 0 min–29 min, 21%–50% A; 29 min–34 min, 50%–70% A; 34 min–42 min, 70%–90% A; 42 min–45 min, 90%–100% A; 45 min–48 min, 100% A. The injection volume was 2 μL, and the detection wavelength was 252 nm.

Within positive ion mode, an electrospray ionization source (ESI) has been used to obtain Q/TOF-MS information in MS^E^ mode with the following conditions: scanning range of m/z 100–1,500 and m/z 556.2771 as a real-time correction by leucine-enkephalin ([M+H]^+^); cone voltage, 40 V; desolation temperature, 350°C; capillary ionization voltage, 3.0 kV; volume flow rate of desolation N2, 600 L/h; ion source temperature, 100°C; and cone blowback N_2_ flow rate, 50 L/h. Low-energy scanning had no collision energy, whereas the high-energy MS^E^ mode sweep collision energy was 35 eV–45 eV. Argon was the collision gas.

### 2.4 Data processing and statistical analysis

To identify PECs in the samples, the UNIFY platform was used to build a library of PECs. For screening the differential metabolites, data preprocessing, such as peak extraction and identification, was performed on the raw UPLC-Q/TOF-MS^E^ data using Waters Progenesis QI (Zhang et al., 2016), and a three-dimensional data matrix consisting of exact m/z, retention time, and peak area obtained after standardization of each sample was output separately and imported into SIMCA14.1 (Umetrics, Sweden) and MetaboAnalyst 5.0 (Xia Lab, Canada). Intra- and intergroup variation in samples was first observed based on unsupervised principal component analysis (PCA). Further supervised sparse partial least-squares discrimination analysis (sPLS-DA) was used to fully reflect the information on the variation between each pair of consecutive groups in the experimental sequence. Similarly, PCA, orthogonal PLS-DA (OPLS-DA), cluster analysis, and t-test were employed to analyze the data for each pair of consecutive groups in the experimental sequence. The variable importance in the projection values, fold change (FC) values, S-plot, and p-values were combined with the actual model parameters to screen for differential metabolites. Finally, the content variation in agarwood samples at different times was investigated using the peak area of the extracted ion chromatogram corresponding to each metabolite.

## 3 Results

### 3.1 Apparent and microscopic characteristics

**Figure 1:**
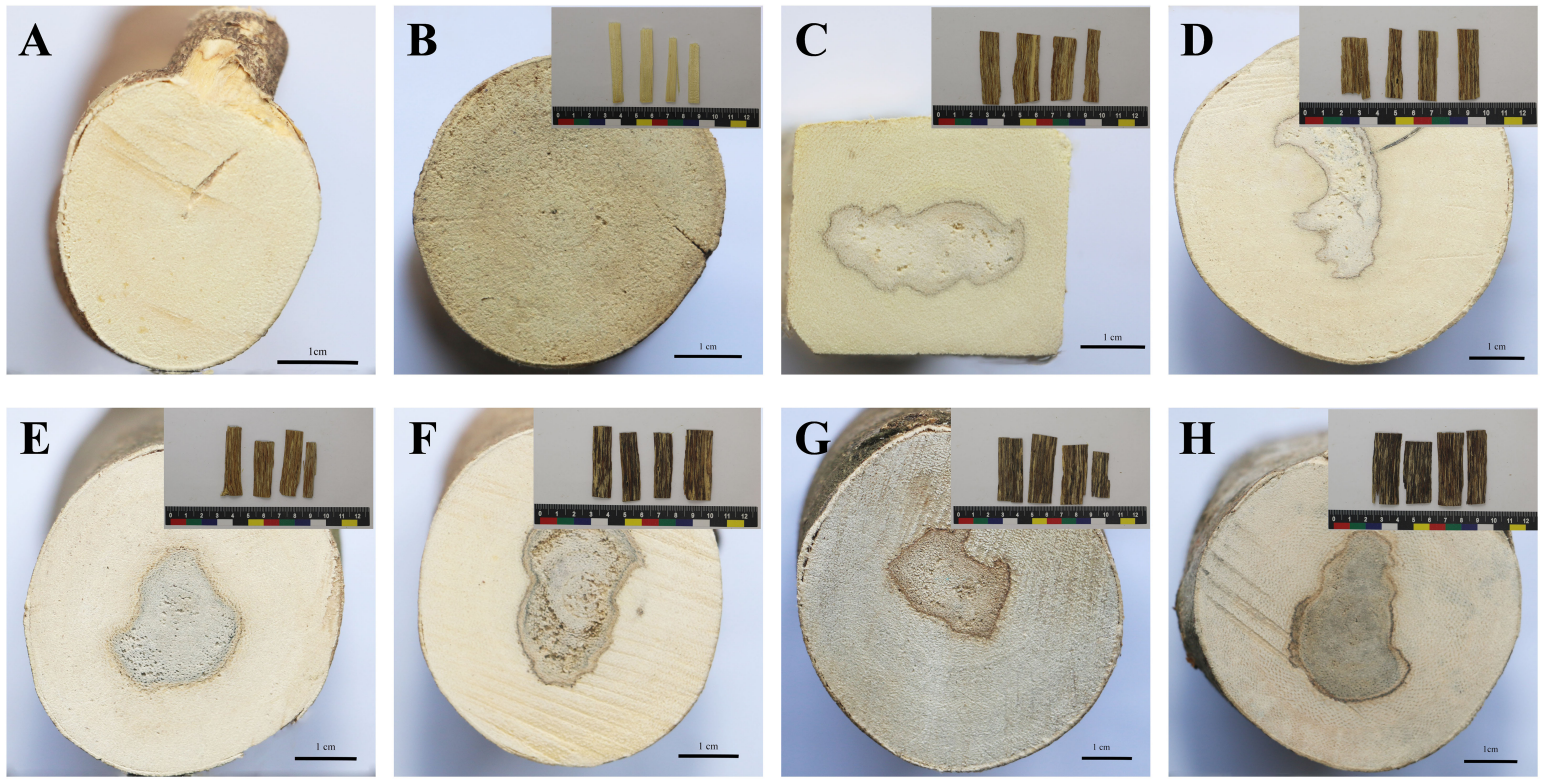
Appearance of the cross section of *A. sinensis* and the appearance of agarwood slices from 0 to 12 months. The cross section of non-infused healthy *A. sinensis* [**(A)**, BM group]. The cross section and agarwood slices of *A. sinensis* that were collected one day [**(B)**, JXY group], 2 months [**(C)**, 2M group], 4 months [**(D)**, 4M group], 6 months [**(E)**, 6M group], 8 months [**(F)**, 8M group], 10 months [**(G)**, 10M group], and 12 months [**(H)**, 12M group] after infusion.

With the prolongation of the agarwood formation time, the resin area expands, and the surface color of the agarwood tends to become darker, which is consistent with the results of this experiment. **Figure 1** depicts the samples of BM (a) and JXY (b) and the agarwood samples at 2, 4, 6, 8, 10, and 12 months after injection with inducer (c-h). The agarwood samples of 2–6 months were interlaced with brown-yellow resin and yellow-white wood, whereas samples of 8–12 months were interlaced with black-brown resin and yellow-white wood.

**Figure 2:**
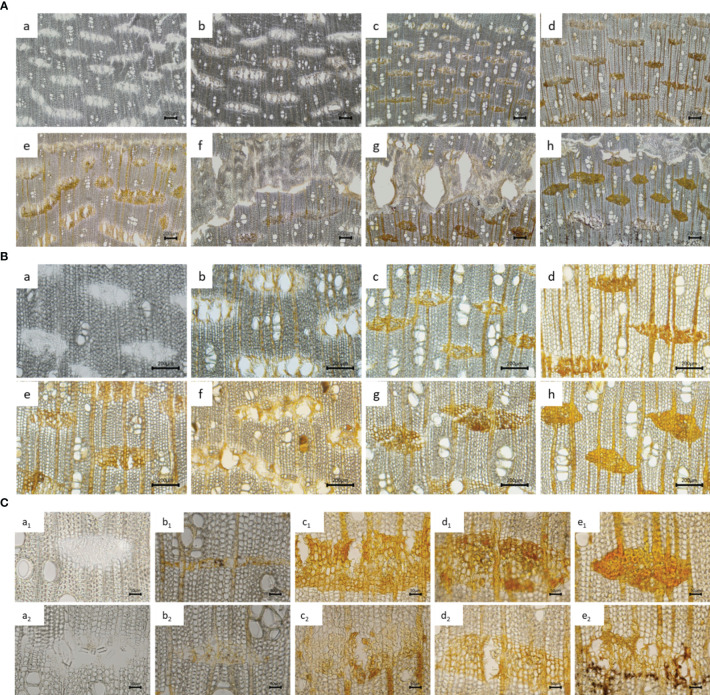
Microscopic characteristics of agarwood samples. **(A, B)** Microscopic characteristics of agarwood samples observed under different microscope objectives [**(A)** 4× and **(B)** 10×]. Samples were collected at 1 day (a), 3 days (b), 30 days (c), 2 months (d), 4 months (e), 6 months (f), 8 months (g), and 12 months (h) after infusion. **(C)** Microscopic characteristics of agarwood samples in different groups observed under a 20× objective microscope. Samples were collected at 1 day (a), 15 days (b), 6 months (c), 8 months (d), and 12 months (e) after infusion.

The information obtained from the appearance was limited; thus, the changes in the inter-xylary phloem of the agarwood after injury were observed using a freezing microtome. After agarwood was induced by injury, from day 3 to day 5, the resin began to fill the inter-xylary phloem near the side of the decay layer, and the degree of filling of the inter-xylary phloem increased over time. From the 4th month after injection, a barrier layer was formed in the inter-xylary phloem on the outside of the agarwood layer, and 6 months after agarwood formation, the barrier layer was obvious (**Figures 2A, B**). The resin color changed from early golden yellow to brownish red. The changes in the inter-xylary phloem of the agarwood samples at different times under a 20× objective microscope are shown in **Figure 2C**. Considering the longitudinal distribution of the inter-xylary phloem in agarwood, a_1_–e_1_ in **Figure 2C** depict the gradually changing trend of the inter-xylary phloem near the decay layer, and a_2_–e_2_ show the changing trend closest to the decaying layer. From a lateral point of view, the resin gradually filled from the cell wall to the inside of the wood parenchyma cells in the inter-xylary phloem, thus filling the inter-xylary phloem. The most filled inter-xylary phloem, according to the longitudinal comparison, was located second closest to the decay layer rather than closest to it. In addition, the starch grains within the inter-xylary phloem and xylem ray parenchyma cells sharply decreased as they were converted to agarwood resin after the agarwood was damaged (Zhang, 2013). The amount of starch grains was significantly lower in xylem rays that intersected with inter-xylary phloem than in xylem rays that did not intersect with the inter-xylary phloem. Interestingly, the number of inter-xylary phloem with high resin filling decreased from 3–4 columns to 1–2 columns after the 6th month.

### 3.2 Quantity and content of PECs

#### 3.2.1 Total PECs

**Figure 3:**
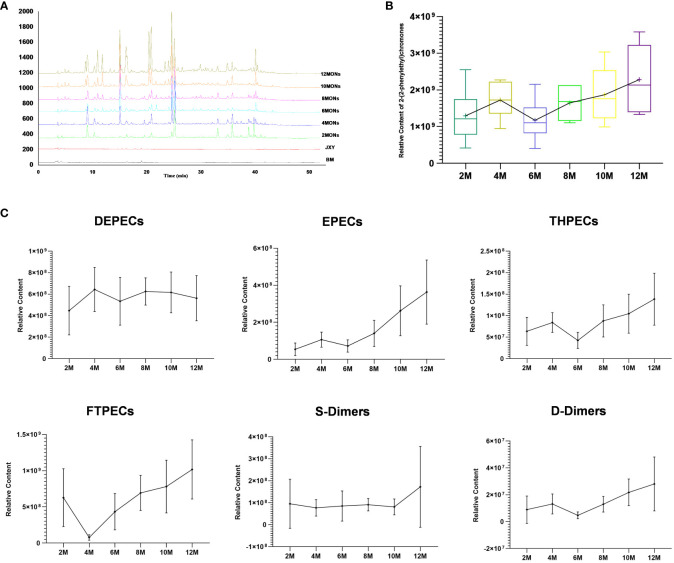
Total PECs at different time points. **(A)** Fingerprints of the total PECs at different time points. **(B)** Total relative content of PECs at different time points. **(C)** Relative content of six structural types of PECs at different time points.

Based on the 7′, 8′ B^+^ diagnostic ions and the characteristic fragment ions of the various structural types of chromones (**Supplementary Figure S2**), the study completed the construction and confirmation of UPLC fingerprints of agarwood samples. The identification is shown in detail in section 2 of the **Supplementary Material**. A total of 108 PECs were identified (**Supplementary Table S1**). Over time, the structure types of PECs in the agarwood samples became increasingly abundant, and the total peak area of PECs also increased (**Figure 3A**). The agarwood samples at 0–12 months were dominated by PECs of three structural types, namely, DEPECs, EPECs, and FTPECs, of which FTPECs were the most abundant. The FTPEC content increased significantly over time (**Table 2**; **Figures 3B, C**). Interestingly, over time, the types and content of PECs in the agarwood samples showed an overall upward trend, but they declined from the 4th to the 6th month.

**Table 2:** Proportion of six structural types of PECs in agarwood samples.

Group	THPECs (%)	S-Dimers (%)	FTPECs (%)	EPECs (%)	DEPECs (%)	D-Dimers (%)	DEPECs+ EPECs (%)
2M	4.91	7.35	48.42	4.18	34.44	0.69	38.63
4M	4.87	4.44	46.46	6.15	37.31	0.76	43.46
6M	3.63	7.26	36.99	6.15	45.57	0.40	51.73
8M	5.32	5.51	42.00	8.50	37.89	0.79	46.39
10M	5.61	4.33	41.83	14.05	33.01	1.17	47.06
12M	6.07	7.55	44.59	15.93	24.64	1.23	40.57

Similarly, the DEPEC content peaked in the 4th month of agarwood formation and then gradually decreased, whereas the trends of the remaining five structural types of PECs were consistent with the changes in the total PEC content (**Figures 3B, C**).

#### 3.2.2 Common PECs

**Figure 4:**
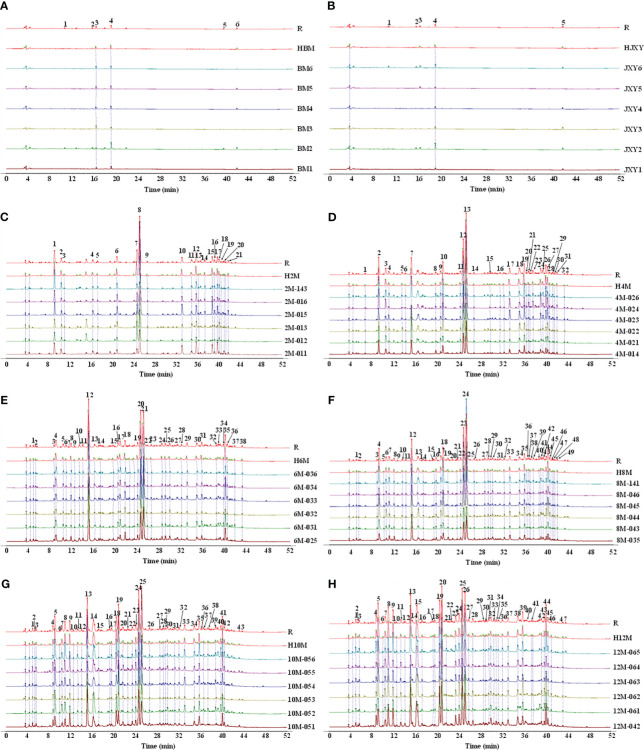
Common compounds in agarwood samples collected from non-infused healthy *A. sinensis* **(A)** and *A. sinensis* one day **(B)**, 2 months **(C)**, 4 months **(D)**, 6 months **(E)**, 8 months **(F)**, 10 months **(G)**, and 12 months **(H)** after infusion.

According to the common peaks from each month, 56 PECs were identified in eight groups (**Supplementary Table S2**). Five common peaks but no chromones were found in the control group or the model group, whereas 22, 33, 29, 45, 41, and 43 common compounds were found in the remaining groups, among which 21, 31, 27, 43, 39, and 41 common chromone compounds were identified, respectively (**Figure 4**). More information about the compounds is shown in **Supplementary Tables S3**–**S8**. Moreover, the chemical composition similarity of the agarwood samples at different times was higher than 0.879, indicating that the main PECs in the agarwood samples were basically the same at different times, and 19 PECs were identified that existed in every group.

#### 3.2.3 Correlation analysis of the ethanol extract and common components

**Figure 5:**
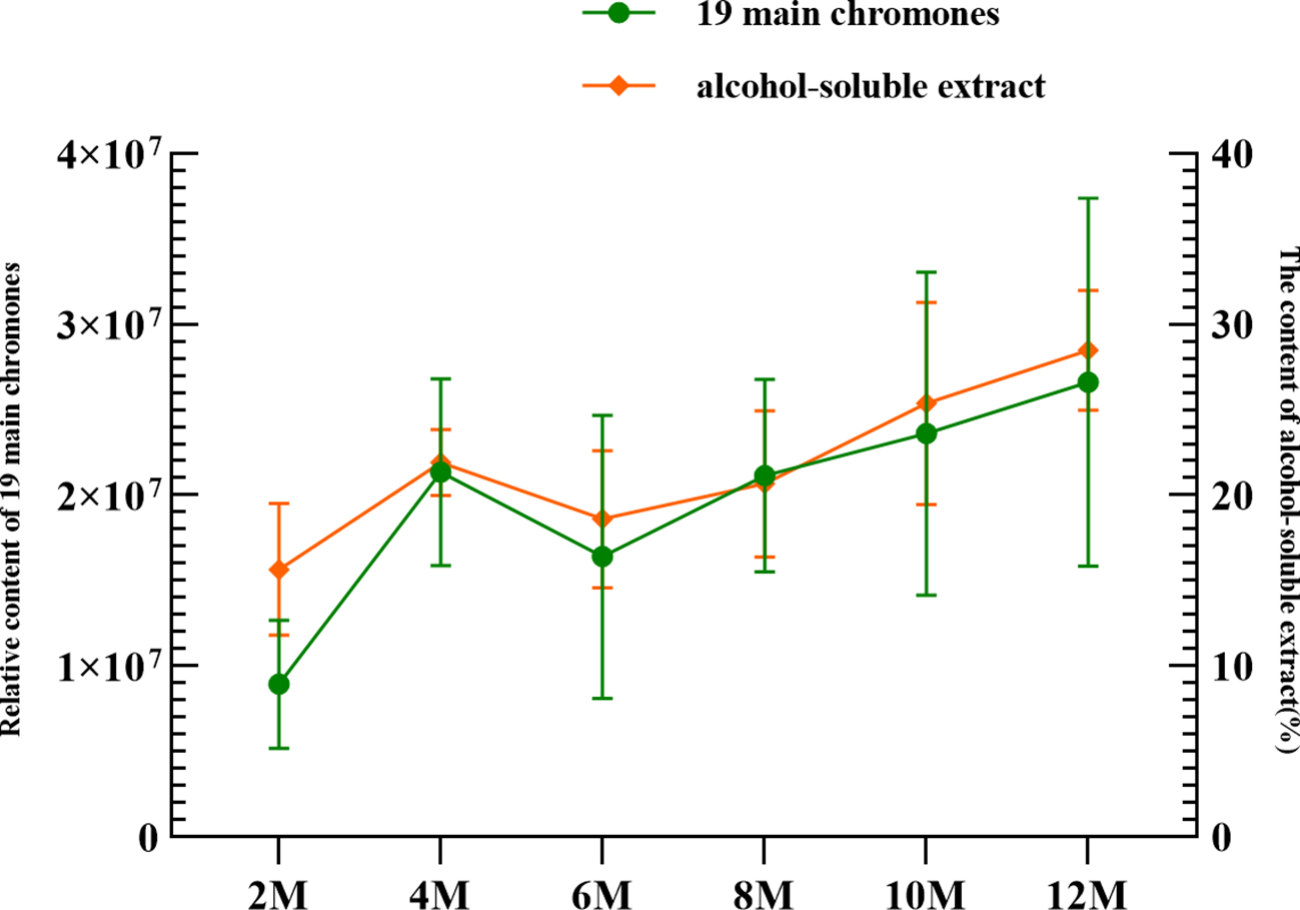
Relationship between 19 main chromones and the alcohol-soluble extract at different sampling times.

The extract content is generally considered an index for evaluating agarwood quality. In this study, the ethanol extract was determined by referencing the Chinese Pharmacopoeia (China Pharmacopoeia Committee, 2020). The ethanol extract contents in agarwood were 4.26% (BM), 6.1% (JXY), 15.66% (2 months, 2M), 21.91% (4 months, 4M), 18.6% (6 months, 6M), 20.67% (8 months, 8M), 25.37% (10 months, 10M), and 28.49% (12 months, 12M) (**Figure 5**). The ethanol extract content from the agarwood sample increased over time, but the extract content of the samples in the 6th and 8th months was lower than that of the agarwood samples in the 4th month.

The trend of the ethanol extract content was similar to the trend of the total content of the abovementioned 19 common components; they both showed a trend of increasing over time (**Figure 5**). First, the content increased from 2 to 4 months. Then, probably due to the appearance of the barrier layer, their content dropped sharply in the 6th month. Finally, after agarwood formation for 10 months, the content of agarwood exceeded that after 4 months.

### 3.3 Analysis of differential compounds in agarwood based on non-targeted metabolomics

#### 3.3.1 Validation

The study investigated the precision and reproducibility of the experimental method and the stability of the samples. The relative standard deviation (RSD) values of the relative retention time and the relative peak area of the common peaks were less than 0.56% and 0.89%, respectively, indicating excellent instrument precision. In the repeatability experiments, the RSD was less than 0.97% and 1.53%, respectively, indicating that the method was reproducible. In the stability test, the samples were injected at intervals of 0 h, 2 h, 4 h, 6 h, 12 h, and 24 h. The RSD values were less than 0.84% and 1.96%, respectively, indicating that the sample solution was stable at room temperature for 24 h.

#### 3.3.2 Differential compounds between samples of each pair of consecutive groups

PCA and OPLS-DA were performed on agarwood samples from BM and 2M (**Supplementary Figure S3**). The two groups of samples showed obvious differences in the types and contents of chemical composition, and the model had good explanatory and prediction abilities. Based on the OPLS-DA model, 24 metabolites were screened based on their variable importance in the projection values and the S-Plot (**Supplementary Figure S3**). They all had higher levels in agarwood samples of the 2M group. Combined with the FC value (threshold 2 or 0.5) and the independent sample t-test (*p* < 0.05), the false peaks, different adducted ion peaks, and noise in the candidate difference variables were manually removed. Finally, between the BM group and the 2M group, a total of 18 compounds with significant differences were identified, which distinguished the two groups of samples well. The differential compounds were qualitatively analyzed according to the fragment ion information, ultraviolet absorption spectra, and high-resolution relative molecular mass (**Supplementary Table S9**).

Healthy *A. sinensis* can induce a defense response when subjected to external damage (physical, chemical, or biologically induced) to synthesize a bacteriostatic defense substance (Rahman and Basak, 1980; Nobuchi and Siripatanadilok, 1991; Ng et al., 1997; Pojanagaroon and Kaewrak, 2005; Ding et al., 2020; Sun et al., 2020). Therefore, in this set of experiments, there was a significant difference in the composition of PECs between the samples in the 2M and BM groups. In addition to the compound with an m/z of 255.1345, the remaining 17 differential metabolites were PECs. The FC values of agarotetrol, oxidoagarochromone B, and oxidoagarochromone A were larger at 462.38, 449.16, and 1,281.6, respectively; these substances accumulated in significant numbers in the first 2 months, suggesting that they originated during the initial phases of agarwood formation.

Using the same process, the study analyzed other samples from each pair of consecutive groups in the experimental sequence. The analysis diagrams are shown in **Supplementary Figures S4**–**S9**, and information on the differential metabolites is presented in **Supplementary Tables S10**–**S15**. Similar to the healthy BM group, there were great differences in the composition of PECs between the JXY group and the 2M group. Among them, agarotetrol, oxidoagarochromone B, oxidoagarochromone A, 6-methoxy-PEC, 6,7-dimethoxy-PEC, PEC, and 6-methoxy-PEC accumulated rapidly and abundantly in the first 2 months of agarwood formation. Therefore, it is speculated that these PECs formed in the early stage.

The differences in the composition of the PECs between the 2M group and the 4M group were not significant. The content of compound 2-[2-(3′-hydroxy-4′-methoxyphenyl)ethyl]-7,8-epoxy-5-methoxy-6-hydroxy-5,6,7,8-tetrahydrochromone was highest in the samples of 2M group, whereas the other differential metabolites were highest in the samples of 4M group. Overall, the PEC content increased in the 4th month. The content of 16 of these compounds showed a significant difference between the two sample groups (p < 0.05). Furthermore, oxidoagarochromone C (isomer) (12.86 min), 6,7-dimethoxy-PEC (34.41 min), 6-methoxy-PEC (isomer) (41.88 min), and S3–2 (isomer) (42.59 min) accumulated rapidly in the 4th month compared with the second month.

There was also a significant difference in the composition of the PECs between the 4-month-old and 6-month-old samples. The final screening yielded 16 different metabolites between the two sample groups. The content of oxidoagarochromone C (isomer), 6-methoxy-2-[2-(3′-hydroxy-4′-methoxyphenyl)ethyl]chromone (isomer) (27.84 min), and two 2-(2-phenethyl)chromone dimers (641.2023 in theory m/z) was higher in samples at 6 months, especially the content of 6-methoxy-2-[2-(3′-hydroxy-4′-methoxyphenyl)ethyl]chromone (isomer) (27.84 min) with an FC value of up to 5.78. The higher levels of the other 12 differential metabolites in the 4-month samples indicated that most of the PECs in the agarwood samples decreased in the 6th month. The decrease was particularly significant for agarotetrol (isomer) (8.88 min), S4–1 (isomer) (37.4 min), and 6-methoxy-PEC (isomer) (41.95 min).

The difference in the composition of PECs in the agarwood formation samples of 6 and 8 months was mainly manifested in the content of 22 differential metabolites. The oxidoagarochromone C (isomer) content was extremely high in samples at 6 months, which was significantly different from that in samples at 8 months. The content of compounds agarotetrol, agarotetrol (isomer), oxidoagarochromone A, 2-(2-phenylethyl)-6, 7-epoxy-5, 8-dihydroxy-5,6,7,8-tetrahydrochromone (isomer), 2-(2-phenylethyl)-6,7-epoxy-5,8-dihydroxy-5,6,7,8-tetrahydrochromone, 8-chloro-5,6,7-trihydroxy-5,6,7,8-tetrahydro-2-(2-phenylethyl)chromone (isomer), and 6-methoxy-2-[2-(3′-hydroxy-4′-methoxyphenyl)ethylchromone (isomer) was two times higher in 8-month samples than in 6-month samples.

Although there was no significant difference in the composition of PECs in samples at 8 and 10 months, the final screening yielded 21 differential metabolites that well distinguished the two groups of samples. The content of 2-[2-(3′-hydroxy-4′-methoxyphenyl)ethyl]-6,7-epoxy-5,8-dihydroxy-5,6,7,8-tetrahydrochromone (isomer), 2-[2-(3′-hydroxy-4′-methoxyphenyl)ethyl]-6,7-ep-5,8-dihydroxy-5,6,7,8-tetrahydrochromone (isomer), oxidoagarochromone B (isomer), 2-[2-(3-hydroxy-4-methoxyphenyl)ethyl]-5,6,7,8-tetrahydroxy-5,6,7,8-tetrahydrochromone (isomer), and 6,7-dimethoxy-PEC (isomer) was more than twice as high in the 10-month sample than in the 8-month sample.

In samples that were 10 and 12 months old, there was little difference in the composition of PECs. The final 30 differential metabolites included 4 THPECs, 6 EPECs/DEPECs, 11 FTPECs, and 8 dimers. Although the two groups of samples were well separated based on these differential compounds, the difference between the two groups of samples was small in terms of FC values and p-values. The content of compounds 5,6,7-trihydroxy-5,6,7,8-tetrahydro-PEC (isomer) (11.22 min), 6-methoxy-2-[2-(3′-hydroxy-4′-methoxyphenyl)ethyl]chromone (isomer) (28.87 min), S6–1 (isomer) (32.97 min), and 6,7-dimethoxy-2-[2-(4′-methoxyphenyl)ethyl]chromone (isomer) (33.28 min) was twice as high in the 10-month sample than in the 12-month sample, whereas compounds S4–1 (isomer) (36.05 min), 2-(2-phenylethyl)chromone, and S-2 (isomer) (47.4 min) showed the opposite trend.

#### 3.3.3 Differential components in agarwood samples

**Figure 6:**
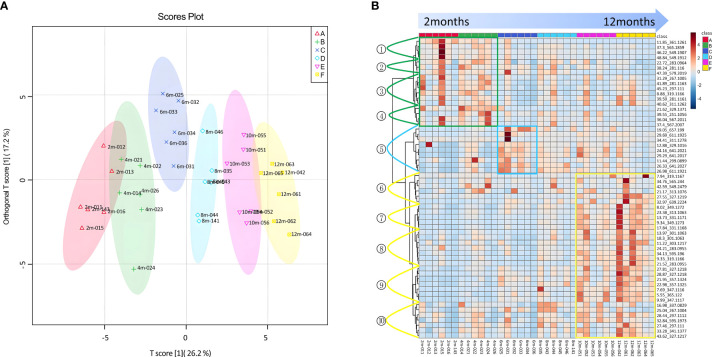
The 58 differential compounds distinguishing between different time points of agarwood formation. **(A)** S-plot based on the OPLA-DA model of agarwood samples at 2–12 months. **(B)** Heatmap of the 58 differential compounds.

Based on the analysis of differential compounds between samples of each pair of consecutive groups in the experimental sequence, 58 components were found in the agarwood samples with different curing times (**Supplementary Table 16**), which could better distinguish the agarwood samples with different sampling times (**Figure 6A**). This study obtained the extracted ion chromatogram data by extracting 58 molecular ion peaks and performed a non-clustered heat map analysis based on the peak area of each compound (**Figure 6B**).

The 58 differential compounds were classified into three categories according to their variation trends. The first group named the transformed type comprised 17 differential compounds, including 2-[2-(3′-hydroxy-4′-methoxyphenyl)ethyl]-7,8-epoxy-5-methoxy-6-hydroxy-5,6,7,8-tetrahydrochromone (isomer) (11.85 min), oxidoagarochromone A (isomer) (21.62 min), oxidoagarochromone A, S7–1 (isomer) (37.3 min), 2-[2-(4′-methoxyphenyl)ethyl]chromone (isomer) (38.24 min), S3–2(isomer) (46.22 min), and S3–2 (isomer) (48.81 min). The content of transformed-type components was the highest at the beginning of formation but gradually decreased with the extension of formation time. The first class of differential metabolites was divided into four subclasses (1–4). Subclass 1 included one EPEC and three S-Dimers; their content was the highest at 2 months, which then decreased to the 12th month with the prolongation of time. Subclass 2 included one DEPEC, one FTPEC, and one S-Dimer; their content was the highest in the 4th month, which then decreased to the 12th month. Subclass 3 included one THPEC and five FTPECs; their content was the highest in the 4th month, decreased sharply in the 6th month, and then increased slowly to 12 months. Subclass 4 included one DEPEC, one FTPEC, and two S-Dimers; similar to subclass 2, their content was the highest in the 4th month and then decreased to the 12th month. Among them, the content of class 3 compounds increased after 12 months and exceeded that at 4 months. The second group consisted of nine differential compounds, including 5,6,7,8-diepoxy-2-[2-(4-hydroxy)ethyl]-5,6,7,8-tetrahydrochromone (isomer) (11.44 min), oxidoagarochromone C (isomer) (12.88 min), D-14 (isomer) (19.05 min), S1–2 (isomer) (26.33 min), and S1–1 (isomer) (26.98 min). Their content increased during agarwood formation but began to decrease after the 6th month. The third group was named the accumulated type and contained 32 differential compounds, including 2-[2-(4′-methoxyphenyl)ethyl]-5,6,7,8-tetrahydroxy-5,6,7,8-tetrahydrochromone (isomer) (8.02 min), 2-(2-phenylethyl)-5,6,7,8-tetrahydroxy-5,6,7,8-tetrahydrochromone (isomer) (9.33 min), oxidoagarochromone B (isomer) (13.73 min), 2-[2-(4′-methoxyphenyl)ethyl]-6,7-epoxy-5,8-dihydroxy-5,6,7,8-tetrahydrochromone (isomer) (17.84 min), 2-[2-(4′-methoxyphenyl)ethyl]-6,7-epoxy-5,8-dihydroxy-5,6,7,8-tetrahydrochromone (isomer) (23.38 min), and 6-methoxy-2-[2-(3′-hydroxy-4′-methoxyphenyl)ethyl]chromone (isomer) (27.81 min). The content of these compounds increased over time. The third class of differential metabolites was divided into five subclasses (6–10). Subclass 6 included one THPEC, two FTPECs, and three S-Dimers, and their content increased continuously with time. Subclass 7 included two THPECs, one DEPEC, and two EPECs; their content increased evidently over time. Subclass 8 included two THPECs, two DEPECs, and two EPECs, and subclass 9 included one THPEC, two EPECs, and four FTPECs. Their content generally increased over time, but subclass 8 decreased at 6 months and subclass 9 decreased at 8 months. The remaining seven compounds (one THPEC, five FTPECs, and one S-Dimer) were classified into subclass 10, and their changes did not have a uniform trend.

## 4 Discussion

Upon exposure to external damage, *A. sinensis* gradually develops a specific layered structure on the injured surface: a normal layer, an agarwood–normal transition layer, an agarwood layer, a decay–agarwood transition layer, and a decay layer (Liu J. et al., 2022). Polysaccharides and starch grains are only present in normal layer and agarwood–normal transition layer. Resin is abundant in agarwood layer and agarwood–normal transition layer, whereas most cells in decayed–agarwood layer and agarwood layer were dead (Liu P. W. et al., 2022). These results from Liu P. W. et al. (2022) are consistent with those of previous studies, and the current experiment showed similar results. In the current study, the results suggested that the amount of inter-xylary phloem with high resin filling decreased after the 6th month; a barrier layer began forming in the 4th month, becoming obvious after 6 months of agarwood formation. Liu et al. (2016) found that the barrier layer was formed by the dedifferentiation and re-division of some inter-xylary phloem on the periphery of the agarwood layer. Therefore, the barrier layer should correspond to the agarwood–normal transition layer. According to the barrier formation mechanism and the morphology of the inter-xylary phloem near the decay layer, it may be that the inter-xylary phloem near the decay layer gradually decays in the initial phase of agarwood formation. The inter-xylary phloem near the normal layer participates in the formation of the barrier layer, resulting in an inter-xylary phloem with high filling, which increases first and then decreases. The agarwood-normal transition layer plays a crucial role in connecting the agarwood layer and normal layer, as it contains living parenchyma cells that have the potential to continuously accumulate resin. This confirms our speculation that as the early decay layer expands outward, the dead cells of the original agarwood layer increase, whereas the dead parenchyma cells no longer continuously accumulate resin, and the formation of the barrier layer has an impact on resin accumulation. It also explains the decrease in the ethanol extract during the 4th and 6th months. Subsequently, the content of the ethanol extract increases as the resin in the inter-xylary phloem of the agarwood layer continues to accumulate over time.

Most researchers use ethanol extracts to extract and separate chromone monomers. The PECs are currently the most common type of separated chromone monomers, and they are also characteristic components of agarwood. The quality of agarwood is believed to improve with higher resin content (Liu et al., 2017). Some studies have found that the content of the ethanol extract is related to the content of resin, which can be used as an indicator to judge the quality of agarwood (Gao X. X. et al., 2012; Li et al., 2017). The PECs are the primary compounds of resin, among which agarotetrol is a vital index for quality assessment (Takamatsu and Ito, 2022a). Therefore, in this study, the content of the ethanol extract and PECs in 12-month agarwood samples was measured. The results suggested that the 19 common PECs, which were consistently presented during the 12 months of agarwood formation, were positively correlated with the total content of the ethanol extract. Presumably, PECs continued to build as the resin content of agarwood increased, leading to an increase in ethanol extracts and an improvement in agarwood quality.

To further investigate the accumulation and transformation of chromones, this study performed UPLC-Q/TOF-MS/MS analysis on each group of samples. According to the order of peaks on the fingerprints, the efflux of THPECs was mostly seen in the retention time range of 5–10 min, whereas DEPECs and EPECs were in the range of 10–27 min. FTPECs and dimers were in the region of 27 min–50 min, with the dimers peaking after 35 min. In conclusion, 108 PECs were identified in all agarwood samples. To exclude the interference of chance factors, this study screened the common components in each group of six samples, and a total of 56 common PECs were identified in all samples. Three structural types of compounds, EPECs, DEPECs, and FTPECs, were predominant, with FTPECs being the most abundant. The content of PECs showed the same changing trend of an overall increase and a decrease at 4–6 months of agarwood formation. Combined with microscopic observations, it is hypothesized that the decline is associated with the formation of the barrier layer.

The process of accumulation is always accompanied by the creation of new compounds and the disappearance of old ones and by the transformation of the structure of the substance. In a previous study, more oxidoagarochromones were observed in the initial phase of resinification. As resinification proceeds, the proportion and amount of agarotetrol increase, so do other derivatives (Okudera and Ito, 2009). The samples of agarwood were analyzed by HPLC-ESI-MS/MS, 2, 4, and 5 years after its formation, as conducted by Yang et al. (2016). It was observed that the total relative contents of EPECs and DEPECs decreased with an increase in formation time, whereas the total relative contents of FTPECs and THPECs increased. Therefore, it can be speculated that there might be a synthesis and transformation of PECs. Subsequently, Takamatsu and Ito (2022b) observed a strong correlation between the proportion of oxido and flinder types; when the oxido-type proportion was low, the flinder-type proportion was high. The study showed that PECs directly change from the oxido type to the flinder type, and the agaro type does not change or it shows very slow conversion to the flinder type. In this study, PECs with FTPEC and THPEC structures were the most abundant, and the contents of PECs with FTPEC, DEPEC, and EPEC structure types were higher than those with the other structure types. An interesting observation is that the proportion of FTPECs shows a change contrary to the total proportion of EPECs and DEPECs. After 6 months of agarwood formation, the proportion of DEPECs continued to decrease, whereas the proportion of EPECs continued to increase. After 12 months of agarwood formation, the total proportion of EPECs and DEPECs showed a generally increasing trend. The proportion of FTPECs decreased, and the proportion of THPECs continued to increase after 6 months of agarwood formation.

To explore the specific substances contributing to accumulation and transformation, this study analyzed the differential metabolites among the different sample groups. Through non-targeted metabolomics analysis between each pair of groups, 58 differential metabolites were selected and divided into three groups. The first type (transformed compounds) contained the compounds that had the highest content at 2–4 months of agarwood formation. The second type of differential metabolites had the highest content in the 6th month of agarwood formation, which was the biggest characteristic of the changing trend of these differential metabolites. The content of the second type of differential compounds increased from the 2nd month to the 6th month and then decreased over time. The third type of differential metabolite (cumulative type) had the highest compound number, and their content displayed a cumulating trend with the extension of time.

In summary, the novelty of this study is that, based on the model of injury-induced agarwood formation in *A. sinensis*, the study systematically explored the accumulation of PECs by following the agarwood samples that had been formed for 0–12 months, analyzing the appearance, microscopic features, the content of ethanol extract, the content of PECs, and the changes of PECs in chemical compositions. It is of scientific significance to investigate the synthesis and transformation of PECs and to promote the development of the agarwood industry.

## 5 Conclusion

In this study, the Agar-Wit was adopted, and non-targeted metabolomics technology was employed to investigate the main metabolites and their differential metabolites in different periods of agarwood formation.

A total of 108 PECs have been identified from agarwood samples collected throughout time, comprising 19 common components and 58 distinct metabolites. The results showed that PECs accumulated over time, resulting in the accumulation of resin content, alcohol-soluble extract content, and resin color. To elucidate, the concentration of PECs and alcohol-soluble extract rose across the first year, and the quantity of ethanol extract was positively linked with the whole amount of 19 common components. The number and total content of PECs increased as well, with the number of FTPEC and THPEC structures staying the most abundant and FTPEC, DEPEC, and EPEC structure types having higher contents. An interesting discovery is that the fraction of FTPECs varies in contrast to the total proportion of EPECs and DEPECs. Furthermore, the starch particles in the parenchyma cells of inter-xylary phloem and rays in xylem gradually decreased, whereas resin accumulated in the inter-xylary phloem, resulting in a darker color in the agarwood samples.

In a nutshell, the dynamic changes in PECs in agarwood layers at different periods provided a scientific basis for the development and utilization of agarwood. Of course, it is unfortunate that the experiment did not evaluate the change in the spatial distribution of PECs. The analysis of the distribution and accumulation of PECs in time and space may reveal further scientific information about the formation of agarwood and its molecular mechanism.

## Data availability statement

The original contributions presented in the study are included in the article/**Supplementary Material**. Further inquiries can be directed to the corresponding author.

## Author contributions

YS: Conceptualization, Investigation, Methodology, Visualization, Writing – original draft, Writing – review & editing. MW: Conceptualization, Data curation, Formal analysis, Methodology, Writing – review & editing. MY: Investigation, Methodology, Resources, Validation, Writing – original draft. JF: Resources, Writing – review & editing. JW: Supervision, Writing – review & editing. YL: Conceptualization, Methodology, Writing – review & editing.
