# Supplementary material for: 2-(2-Phenylethyl)chromones increase in Aquilaria sinensis with the formation of agarwood
Source: Front Plant Sci. 2024 Jul 11;15:1437105. doi: 10.3389/fpls.2024.1437105 (PMC11273687; doi:10.3389/fpls.2024.1437105)
Supplement: Supplementary file 1 [file DataSheet_1.docx]

Supplementary material

2-(2-phenylethyl)chromones increase in *Aquilaria sinensis* with the formation of agarwood

# Comparison of extraction efficiency

**Supplementary Fig. S1.** Comparison of extraction efficiency. A: Methanol ultrasound, B: Ethanol ultrasound, C: Methanol reflux, D: Ethanol reflux.

# Identification of 2-(2-phenylethyl)chromones in agarwood

In this study, a 2-(2-phenylethyl)chromones identification database was constructed by UNIFY based on the published 2-(2-phenylethyl)chromones isolated or detected in agarwood or other family plants. Furthermore, the research investigated the mass spectrometry lysis mode of 2-(2-phenylethyl)chromones and found that the 7’, 8’B^+^ fragment ions obtained by breaking the single bond of C7-C8 were stable in the secondary mass spectrometry of such compounds and had good mass spectrometry response values. Thus, 7’, 8’B^+^ can be used as a diagnostic ion for 2-(2-phenylethyl)chromones in agarwood with high resolution mass-to-charge ratios of 91.0542 (no substitutions), 107.0497 (OH substitution), 121.0653 (OCH3 substitution) and 137.0630 (OH and OCH3 substitutions) respectively (Supplementary Fig. S2.). But the type and position of the B-ring substituent could affect the ionic form and response of the 2-(2-phenylethyl)chromone fragments. Besides, each of the five structural types of 2-(2-phenylethyl)chromones has its own specific cleavage pathway, in which THPECs can continuously lose 2 molecules of H2O; DEPECs can continuously lose 2 molecules of CO; EPECs can continuously lose 1 molecule of H2O followed by 1 molecule of CO; FTPECs can undergo RDA rearrangement in the B^+^C7’-C8’ and C rings; it can happen in dimers that chemical bonds between monomers are broken and corresponding fragments are obtained. The aforementioned characteristic fragment ions can be used to distinguish different structural types of 2-(2-phenylethyl)chromones.

**Supplementary Fig. S2.** Total Ion Chromatograms (TIC) under low-energy (F) and high-energy (E) conditions, as well as Extracted Ion Chromatograms (EIC) for four different diagnostic ions (A - D).

# Construction of a compound library of 2-(2-phenylethyl)chromones based on UNIFY

Based on the constructed UNIFY database, a total of 108 2-(2-phenylethyl)chromones were identified from all samples (Supplementary Table 1). In order to minimize the error caused by chance factors, we screened the common components of six samples at each sampling time point, and a total of 58 2-(2-phenylethyl)chromones were identified in the samples at different sampling time points (Supplementary Tables 2). No shared 2-(2-phenylethyl)chromones were found in the BM and JXY groups, and 21, 31, 27, 43, 39 and 41 shared 2-(2-phenylethyl)chromones were identified in the 2M, 4M, 6M, 8M, 10M and 12M groups, respectively (Supplementary Tables 3-8).

**Supplementary Table S1.** 108 2-(2-phenylethyl)chromones identified based on UNIFY.

| **t_R_ (min)** | **Proposed compound** | **Formula** | **Molecular mass (Da)** | **Measured** **molecular mass (Da)** | **Mass number error (ppm)** | **The expected fragment ion was found** |
| --- | --- | --- | --- | --- | --- | --- |
| 4.43 | 2-[2-(4'-hydroxy)ethyl]-5,6,7,8-tetrahydroxy-5,6,7,8-tetrahydrochromone | C_17_H_18_O_7_ | 334.1053 | 334.1035 | -5.1 | 4 |
| 4.52 | 2-[2-(3-hydroxy-4-methoxyphenyl)ethyl]-5,6,7,8-tetrahydroxy-5,6,7,8-tetrahydrochromone | C_18_H_20_O_8_ | 364.1158 | 364.1136 | -6 | 2 |
| 4.94 | 2-[2-(4'-hydroxy)ethyl]-5,6,7,8-tetrahydroxy-5,6,7,8-tetrahydrochromone | C_17_H_18_O_7_ | 334.1053 | 334.1032 | -6.1 | 2 |
| 5.02 | 2-[2-(3-hydroxy-4-methoxyphenyl)ethyl]-5,6,7,8-tetrahydroxy-5,6,7,8-tetrahydrochromone | C_18_H_20_O_8_ | 364.1158 | 364.1141 | -4.6 | 6 |
| 5.29 | 2-[2-(4'-hydroxy)ethyl]-5,6,7,8-tetrahydroxy-5,6,7,8-tetrahydrochromone | C_17_H_18_O_7_ | 334.1053 | 334.1031 | -6.3 | 0 |
| 5.52 | 2-[2-(3-hydroxy-4-methoxyphenyl)ethyl]-5,6,7,8-tetrahydroxy-5,6,7,8-tetrahydrochromone | C_18_H_20_O_8_ | 364.1158 | 364.1142 | -4.6 | 5 |
| 5.76 | 2-[2-(4'-hydroxy)ethyl]-5,6,7,8-tetrahydroxy-5,6,7,8-tetrahydrochromone | C_17_H_18_O_7_ | 334.1053 | 334.1033 | -5.8 | 5 |
| 6.26 | 2-[2-(4'-hydroxy)ethyl]-6,7-epoxy-5,8-dihydroxy-5,6,7,8-tetrahydrochromone | C_17_H_16_O_6_ | 316.0947 | 316.0931 | -5 | 5 |
| 6.27 | 2-[2-(4'-hydroxy)ethyl]-5,6,7,8-tetrahydroxy-5,6,7,8-tetrahydrochromone | C_17_H_18_O_7_ | 334.1053 | 334.1034 | -5.5 | 6 |
| 6.5 | 5,6,7-trihydroxy-5,6,7,8-tetrahydro-2-[2-(3'-hydroxyphenyl)ethyl]chromone | C_17_H_18_O_6_ | 318.1103 | 318.1089 | -4.5 | 1 |
| 6.6 | 6,7,8-trihydroxy-5-methoxy-5,6,7,8-tetrahydro-2-[2-(4'-hydroxyphenyl)ethyl]chromone | C_18_H_20_O_7_ | 348.1209 | 348.1190 | -5.4 | 3 |
| 7.69 | 2-[2-(3'-hydroxy-4'-methoxyphenyl)ethyl]-6,7-epoxy-5,8-dihydroxy-5,6,7,8-tetrahydrochromone | C_18_H_18_O_7_ | 346.1053 | 346.1035 | -5.0 | 3 |
| 7.97 | 2-(2-phenylethyl)-5,6,7,8-tetrahydroxy-5,6,7,8-tetrahydrochromone | C_17_H_18_O_6_ | 318.1103 | 318.1086 | -5.4 | 6 |
| 8.02 | 2-[2-(4'-methoxyphenyl)ethyl]-5,6,7,8-tetrahydroxy-5,6,7,8-tetrahydrochromone | C_18_H_20_O_7_ | 348.1209 | 348.1190 | -5.4 | 5 |
| 8.9 | 2-(2-phenylethyl)-5,6,7,8-tetrahydroxy-5,6,7,8-tetrahydrochromone | C_17_H_18_O_6_ | 318.1103 | 318.1087 | -5.2 | 6 |
| 8.93 | 2-[2-(4'-methoxyphenyl)ethyl]-5,6,7,8-tetrahydroxy-5,6,7,8-tetrahydrochromone | C_18_H_20_O_7_ | 348.1209 | 348.1192 | -4.8 | 5 |
| 9.1 | 6,7-dihydroxy-5,6,7,8-tetrahydro-2-[2-(3'-hydroxyphenyl)ethyl]chromone | C_17_H_18_O_5_ | 302.1154 | 302.1135 | -6.3 | 1 |
| 9.2 | 6,7-dihydroxy-5,6,7,8-tetrahydro-2-[2-(3'-methoxyphenyl)ethyl]chromone | C_18_H_20_O_5_ | 316.1311 | 316.1312 | 0.3 | 0 |
| 9.35 | 2-(2-phenylethyl)-5,6,7,8-tetrahydroxy-5,6,7,8-tetrahydrochromone | C_17_H_18_O_6_ | 318.1103 | 318.1088 | -5.0 | 6 |
| 9.36 | 2-[2-(4'-methoxyphenyl)ethyl]-5,6,7,8-tetrahydroxy-5,6,7,8-tetrahydrochromone | C_18_H_20_O_7_ | 348.1209 | 348.1194 | -4.4 | 5 |
| 9.52 | 5,6,7-trihydroxy-5,6,7,8-tetrahydro-2-(2-phenylethyl)chromone | C_17_H_18_O_5_ | 302.1154 | 302.1136 | -5.9 | 3 |
| 9.59 | 8-chloro-5,6,7-trihydroxy-5,6,7,8-tetrahydro-2-[2-(3'-hydroxy-4'-methoxyphenyl)ethyl]chromone | C_18_H_19_ClO_7_ | 382.0819 | 382.0801 | -4.8 | 2 |
| 10.02 | 2-[2-(3'-hydroxy-4'-methoxyphenyl)ethyl]-6,7-epoxy-5,8-dihydroxy-5,6,7,8-tetrahydrochromone | C_18_H_18_O_7_ | 346.1053 | 346.1038 | -4.1 | 5 |
| 10.51 | 2-[2-(4'-methoxyphenyl)ethyl]-6,7-epoxy-5,8-dihydroxy-5,6,7,8-tetrahydrochromone | C_18_H_18_O_6_ | 330.1103 | 330.1088 | -4.6 | 2 |
| 11.1 | 6,7-dihydroxy-5,6,7,8-tetrahydro-2-[2-(3'-hydroxy-4'-methoxyphenyl)ethyl]chromone | C_18_H_20_O_6_ | 332.1260 | 332.1241 | -5.3 | 3 |
| 11.14 | 2-[2-(4'-methoxyphenyl)ethyl]-6,7-epoxy-5,8-dihydroxy-5,6,7,8-tetrahydrochromone | C_18_H_18_O_6_ | 330.1103 | 330.1086 | -5.4 | 4 |
| 11.24 | 5,6,7-trihydroxy-5,6,7,8-tetrahydro-2-(2-phenylethyl)chromone | C_17_H_18_O_5_ | 302.1154 | 302.1138 | -5.3 | 4 |
| 11.44 | 5,6,7,8-diepoxy-2-[2-(4-hydroxy)ethyl]-5,6,7,8-tetrahydrochromone | C_17_H_14_O_5_ | 298.0841 | 298.0826 | -5.1 | 3 |
| 11.82 | 2-[2-(3'-hydroxy-4'-methoxyphenyl)ethyl]-7,8-epoxy-5-methoxy-6-hydroxy-5,6,7,8-tetrahydrochromone | C_19_H_20_O_7_ | 360.1209 | 360.1188 | -5.9 | 3 |
| 11.99 | 5,6,7,8-diepoxy-2-[2-(3-hydroxy-4-methoxyphenyl)ethyl]-5,6,7,8-tetrahydrochromone(Oxidoagarochromone C) | C_18_H_16_O_6_ | 328.0947 | 328.0928 | -5.7 | 2 |
| 12.12 | 2-[2-(4'-hydroxy)ethyl]-6,7-epoxy-5,8-dihydroxy-5,6,7,8-tetrahydrochromone | C_17_H_16_O_6_ | 316.0947 | 316.0933 | -4.5 | 3 |
| 12.64 | 5,6,7,8-diepoxy-2-[2-(3-hydroxy-4-methoxyphenyl)ethyl]-5,6,7,8-tetrahydrochromone(Oxidoagarochromone C) | C_18_H_16_O_6_ | 328.0947 | 328.0928 | -5.7 | 2 |
| 12.86 | 5,6,7,8-diepoxy-2-[2-(3-hydroxy-4-methoxyphenyl)ethyl]-5,6,7,8-tetrahydrochromone(Oxidoagarochromone C) | C_18_H_16_O_6_ | 328.0947 | 328.0933 | -4.2 | 4 |
| 13.78 | 2-[2-(4'-methoxyphenyl)ethyl]-6,7-epoxy-5,8-dihydroxy-5,6,7,8-tetrahydrochromone | C_18_H_18_O_6_ | 330.1103 | 330.1087 | -4.9 | 4 |
| 14.01 | 2-(2-phenylethyl)-6,7-epoxy-5,8-dihydroxy-5,6,7,8-tetrahydrochromone | C_17_H_16_O_5_ | 300.0998 | 300.0984 | -4.7 | 5 |
| 14.58 | 5,6,7,8-diepoxy-2-[2-(3-hydroxy-4-methoxyphenyl)ethyl]-5,6,7,8-tetrahydrochromone(Oxidoagarochromone C) | C_18_H_16_O_6_ | 328.0947 | 328.0931 | -4.7 | 2 |
| 14.68 | 2-[2-(3'-hydroxy-4'-methoxyphenyl)ethyl]-7,8-epoxy-5-methoxy-6-hydroxy-5,6,7,8-tetrahydrochromone | C_19_H_20_O_7_ | 360.1209 | 360.1187 | -6.0 | 2 |
| 15.27 | 5,6,7,8-diepoxy-2-[2-(4-hydroxy)ethyl]-5,6,7,8-tetrahydrochromone | C_17_H_14_O_5_ | 298.0841 | 298.0822 | -6.3 | 3 |
| 15.5 | 6,7-dihydroxy-5,6,7,8-tetrahydro-2-(2-phenylethyl)chromone | C_17_H_18_O_4_ | 286.1205 | 286.1183 | -7.8 | 2 |
| 16.2 | D6-new | C_35_H_28_O_9_ | 592.1733 | 592.1733 | -6.1 | 1 |
| 16.55 | 8-chloro-5,6,7-trihydroxy-5,6,7,8-tetrahydro-2-[2-(4'-methoxyphenyl)ethyl]chromone | C_18_H_19_ClO_6_ | 366.0870 | 366.0853 | -4.8 | 4 |
| 16.58 | DEPECs3 | C_18_H_16_O_5_ | 312.0998 | 312.0982 | -5.0 | 2 |
| 16.99 | 8-chloro-5,6,7-trihydroxy-5,6,7,8-tetrahydro-2-(2-phenylethyl)chromone | C_17_H_17_ClO_5_ | 336.0765 | 336.0749 | -4.5 | 5 |
| 17.9 | 2-[2-(4'-methoxyphenyl)ethyl]-6,7-epoxy-5,8-dihydroxy-5,6,7,8-tetrahydrochromone | C_18_H_18_O_6_ | 330.1103 | 330.1089 | -4.4 | 4 |
| 18.35 | 2-(2-phenylethyl)-6,7-epoxy-5,8-dihydroxy-5,6,7,8-tetrahydrochromone | C_17_H_16_O_5_ | 300.0998 | 300.0986 | -3.9 | 5 |
| 19.01 | 5,6,7,8-diepoxy-2-[2-(4-methoxyphenyl)ethyl]-5,6,7,8-tetrahydrochromone(Oxidoagarochromone B) | C_18_H_16_O_5_ | 312.0998 | 312.0984 | -4.5 | 4 |
| 19.98 | 6-hydroxy-2-[2-(3'-hydroxy-4'-methoxyphenyl)ethyl]chromone | C_18_H_16_O_5_ | 312.0998 | 312.0979 | -5.9 | 2 |
| 20.83 | 5,6,7,8-diepoxy-2-[2-(4-methoxyphenyl)ethyl]-5,6,7,8-tetrahydrochromone(Oxidoagarochromone B) | C_18_H_16_O_5_ | 312.0998 | 312.0983 | -4.8 | 2 |
| 21.21 | - |  |  |  |  |  |
| 21.52 | Oxidoagarochromone A | C_17_H_14_O_4_ | 282.0892 | 282.0874 | -6.4 | 2 |
| 21.46 | (Oxidoagarochromone C) |  |  | 329.1004 |  |  |
| 22.17 | 5,6,7,8-diepoxy-2-[2-(4-methoxyphenyl)ethyl]-5,6,7,8-tetrahydrochromone(Oxidoagarochromone B) | C_18_H_16_O_5_ | 312.0998 | 312.0984 | -4.4 | 4 |
| 22.74 | 5,6,7,8-diepoxy-2-(2-phenylethyl)-5,6,7,8-tetrahydrochromone(Oxidoagarochromone A) | C_17_H_14_O_4_ | 282.0892 | 282.0877 | -5.4 | 4 |
| 23.4 | 6,7-dihydroxy-2-[2-(4'-methoxyphenyl)ethyl]chromone | C_18_H_16_O_5_ | 312.0998 | 312.0983 | -4.6 | 2 |
| 24.23 | 5,6,7,8-diepoxy-2-(2-phenylethyl)-5,6,7,8-tetrahydrochromone(Oxidoagarochromone A) | C_17_H_14_O_4_ | 282.0892 | 282.0874 | -6.6 | 2 |
| 25.06 | 2-[2-hydroxy-2-(2-phenylethyl)]chromone | C_17_H_14_O_3_ | 266.0943 | 266.0924 | -7.0 | 1 |
| 25.43 | 2-[2-(2'-hydroxy-4'-methoxyphenyl)ethyl]chromone | C_18_H_16_O_4_ | 296.1049 | 296.1032 | -5.7 | 2 |
| 25.57 | 6-hydroxy-7-methoxy-2-[2-(4'-methoxyphenyl)ethyl]chromone | C_19_H_18_O_5_ | 326.1154 | 326.1136 | -5.7 | 2 |
| 25.92 | 6-methoxy-2-[2-(4'-hydroxyphenyl)ethyl]chromone | C_18_H_16_O_4_ | 296.1049 | 296.1026 | -7.7 | 2 |
| 25.97 | D9-2 | C_36_H_34_O_11_ | 642.2101 | 642.2086 | -2.4 | 0 |
| 26.13 | 6-hydroxy-2-(2-phenylethyl)chromone | C_17_H_14_O_3_ | 266.0943 | 266.0922 | -7.7 | 5 |
| 26.36 | S1-2 | C_36_H_32_O_11_ | 640.1945 | 640.1950 | 0.9 | 1 |
| 27.01 | S1-1 | C_35_H_30_O_10_ | 610.1839 | 610.1841 | 0.3 | 1 |
| 27.44 | 6-hydroxy-7-methoxy-2-[2-(4'-methoxyphenyl)ethyl]chromone | C_19_H_18_O_5_ | 326.1154 | 326.1140 | -4.4 | 2 |
| 27.44 | 6-methoxy-2-[2-(4'-hydroxyphenyl)ethyl]chromone | C_18_H_16_O_4_ | 296.1049 | 296.1032 | -5.6 | 3 |
| 27.83 | 6-methoxy-2-[2-(3'-hydroxy-4'-methoxyphenyl)ethyl]chromone | C_19_H_18_O_5_ | 326.1154 | 326.1142 | -3.6 | 3 |
| 28.45 | 6-hydroxy-7-methoxy-2-(2-phenylethyl)chromone | C_18_H_16_O_4_ | 296.1049 | 296.1034 | -5.0 | 5 |
| 28.88 | 6-methoxy-2-[2-(3'-hydroxy-4'-methoxyphenyl)ethyl]chromone | C_19_H_18_O_5_ | 326.1154 | 326.1138 | -5.1 | 2 |
| 29.27 | S1-2 | C_36_H_32_O_11_ | 640.1945 | 640.1944 | 0.0 | 1 |
| 29.67 | S1-1 | C_35_H_30_O_10_ | 610.1839 | 610.1835 | -0.7 | 1 |
| 30.15 | 6-hydroxy-2-[2-(4'-methoxyphenyl)ethyl]chromone | C_18_H_16_O_4_ | 296.1049 | 296.1033 | -5.4 | 2 |
| 30.67 | S1-2 | C_36_H_32_O_11_ | 640.1945 | 640.1943 | -0.3 | 1 |
| 31.04 | 5-hydroxy-6,7-dimethoxy-2-[2-(4'-hydroxyphenyl)ethyl]chromone | C_19_H_18_O_6_ | 342.1103 | 342.1085 | -5.4 | 1 |
| 31.27 | 6-hydroxy-2-(2-phenylethyl)chromone | C_17_H_14_O_3_ | 266.0943 | 266.0926 | -6.4 | 5 |
| 33.29 | 6,7-dimethoxy-2-[2-(4'-methoxyphenyl)ethyl]chromone | C_20_H_20_O_5_ | 340.1311 | 340.1298 | -3.8 | 2 |
| 34.42 | 6,7-dimethoxy-2-(2-phenylethyl)chromone | C_19_H_18_O_4_ | 310.1205 | 310.1190 | -4.8 | 4 |
| 34.42 | 6,7-dimethoxy-2-[2-(3'-hydroxyphenyl)ethyl]chromone | C_19_H_18_O_5_ | 326.1154 | 326.1132 | -6.9 | 2 |
| 35.13 | S8-1 | C_34_H_28_O_8_ | 564.1784 | 564.1786 | 0.4 | 0 |
| 35.37 | S8-2 | C_35_H_30_O_9_ | 594.1890 | 594.1892 | 0.4 | 0 |
| 35.74 | S4-6 | C_36_H_32_O_10_ | 624.1996 | 624.1990 | -0.8 | 0 |
| 35.85 | S8-2 | C_35_H_30_O_9_ | 594.1890 | 594.1886 | -0.6 | 0 |
| 36.05 | D1-4 | C_34_H_30_O_8_ | 566.1941 | 566.1934 | -1.1 | 0 |
| 36.29 | S-1 | C_34_H_28_O_8_ | 564.1784 | 564.1779 | -0.9 | 0 |
| 36.29 | S7-1 | C_34_H_28_O_8_ | 564.1784 | 564.1779 | -0.9 | 1 |
| 36.43 | D1-3 | C_35_H_32_O_9_ | 596.2046 | 596.2036 | -1.7 | 0 |
| 36.5 | S-2 | C_35_H_30_O_8_ | 578.1941 | 578.1936 | -0.8 | 1 |
| 36.9 | S8-2 | C_35_H_30_O_9_ | 594.1890 | 594.1891 | 0.2 | 0 |
| 37.32 | S7-1 | C_34_H_28_O_8_ | 564.1784 | 564.1779 | -0.9 | 1 |
| 37.46 | D1-4 | C_34_H_30_O_8_ | 566.1941 | 566.1930 | -1.9 | 1 |
| 37.93 | S8-2 | C_35_H_30_O_9_ | 594.1890 | 594.1895 | 0.8 | 1 |
| 38.25 | 2-[2-(4'-methoxyphenyl)ethyl]chromone | C_18_H_16_O_3_ | 280.1099 | 280.1083 | -5.9 | 2 |
| 38.37 | S8-2 | C_35_H_30_O_9_ | 594.1890 | 594.1891 | 0.2 | 1 |
| 39.06 | 5-chloro-6-hydroxy-2-[2-(4'-methoxyphenyl)ethyl]chromone | C_18_H_15_ClO_4_ | 330.0659 | 330.0641 | -5.3 | 0 |
| 39.12 | S-2 | C_35_H_30_O_8_ | 578.1941 | 578.1925 | -2.7 | 1 |
| 39.56 | 2-(2-phenylethyl)chromone | C_35_H_30_O_5_ | 250.1056 | 251.0056 | -2.9 | 1 |
| 39.57 | 6-methoxy-2-(2-phenylethyl)chromone | C_18_H_16_O_3_ | 280.1099 | 280.1082 | -6.4 | 5 |
| 40.68 | 6-methoxy-2-[2-(4'-methoxyphenyl)ethyl]chromone | C_19_H_18_O_4_ | 310.1205 | 310.1191 | -4.6 | 5 |
| 41.87 | 6-methoxy-2-(2-phenylethyl)chromone | C_18_H_16_O_3_ | 280.1099 | 280.1084 | -5.4 | 5 |
| 42 | S3-2 | C_34_H_28_O_7_ | 548.1835 | 548.1832 | -0.5 | 1 |
| 43.17 | S6-2 | C_36_H_32_O_9_ | 608.2046 | 608.2036 | -1.7 | 1 |
| 43.65 | 6-hydroxy-7-methoxy-2-[2-(4'-methoxyphenyl)ethyl]chromone | C_19_H_18_O_5_ | 326.1154 | 326.1136 | -5.6 | 2 |
| 45.17 | 6-hydroxy-7-methoxy-2-(2-phenylethyl)chromone | C_18_H_16_O_4_ | 296.1049 | 296.1032 | -5.7 | 4 |
| 45.22 | S-2 | C_35_H_30_O_8_ | 578.1941 | 578.1937 | -0.6 | 1 |
| 46.14 | S6-2 | C_36_H_32_O_9_ | 608.2046 | 608.2042 | -0.7 | 1 |
| 46.26 | S3-2 | C_34_H_28_O_7_ | 548.1835 | 548.1830 | -0.9 | 1 |
| 47.13 | 5,6,8-trimethoxy-2-(2-phenylethyl)chromone | C_17_H_14_O_5_ | 298.0841 | 298.0823 | -6.2 | 1 |
| 47.43 | S-2 | C_35_H_30_O_8_ | 578.1941 | 578.1941 | 0.0 | 1 |
| 47.89 | D4-4 | C_34_H_28_O_7_ | 548.1835 | 548.1828 | -1.4 | 0 |

**Supplementary Table S2.** 58 2-(2-Phenylethyl)chromone compounds in agarwood during different resin induction periods identified based on UNIFY.

| **t_R_ (min)** | **Proposed compound** | **Formula** | **Molecular mass (Da)** | **Measured molecular mass (Da)** | **Mass number error (ppm)** | **The expected fragment ion was found** | **2M** | **4M** | **6M** | **8M** | **10M** | **12M** |
| --- | --- | --- | --- | --- | --- | --- | --- | --- | --- | --- | --- | --- |
| 5.02 | 2-[2-(3-hydroxy-4-methoxyphenyl)ethyl]-5,6,7,8-tetrahydroxy-5,6,7,8-tetrahydrochromone | C_18_H_20_O_8_ | 364.1158 | 364.1141 | -4.6 | 6 |  |  | √ | √ | √ | √ |
| 5.03 | 6.7.8-trihydroxy-5-methoxy-5.6.7.8-tetrahydro-2-[2-(4'-hydroxy-3'-methoxyphenyl)ethyl]chromone | C_18_H_20_O_8_ | 364.1158 | 364.1146 | -3.4 | 1 |  |  |  |  | √ |  |
| 5.52 | 2-[2-(3-hydroxy-4-methoxyphenyl)ethyl]-5,6,7,8-tetrahydroxy-5,6,7,8-tetrahydrochromone | C_18_H_20_O_8_ | 364.1158 | 364.1142 | -4.6 | 5 |  |  | √ | √ | √ | √ |
| 6.27 | 2-[2-(4'-hydroxy)ethyl]-5,6,7,8-tetrahydroxy-5,6,7,8-tetrahydrochromone | C_17_H_18_O_7_ | 334.1053 | 334.1034 | -5.5 | 6 |  | √ |  |  |  |  |
| 7.69 | 2-[2-(3'-hydroxy-4'-methoxyphenyl)ethyl]-6,7-epoxy-5,8-dihydroxy-5,6,7,8-tetrahydrochromone | C_18_H_18_O_7_ | 346.1053 | 346.1035 | -5.0 | 3 |  |  | √ | √ | √ | √ |
| 7.97 | 2-(2-phenylethyl)-5,6,7,8-tetrahydroxy-5,6,7,8-tetrahydrochromone | C_17_H_18_O_6_ | 318.1103 | 318.1086 | -5.4 | 6 | √ | √ | √ | √ | √ | √ |
| 8.53 | 5.6.7-trihydroxy-5.6.7.8-tetrahydro-2-[2-(3'-hydroxyphenyl)ethyl]chromone | C_17_H_18_O_6_ | 318.1103 | 318.1090 | -4.3 | 3 |  |  |  | √ | √ | √ |
| 8.9 | 2-(2-phenylethyl)-5,6,7,8-tetrahydroxy-5,6,7,8-tetrahydrochromone | C_17_H_18_O_6_ | 318.1103 | 318.1087 | -5.2 | 6 | √ | √ | √ | √ | √ | √ |
| 9.35 | 2-(2-phenylethyl)-5,6,7,8-tetrahydroxy-5,6,7,8-tetrahydrochromone | C_17_H_18_O_6_ | 318.1103 | 318.1088 | -5.0 | 6 | √ | √ | √ | √ | √ | √ |
| 10.02 | 2-[2-(3'-hydroxy-4'-methoxyphenyl)ethyl]-6,7-epoxy-5,8-dihydroxy-5,6,7,8-tetrahydrochromone | C_18_H_18_O_7_ | 346.1053 | 346.1038 | -4.1 | 5 |  |  | √ | √ | √ | √ |
| 10.51 | 2-[2-(4'-methoxyphenyl)ethyl]-6,7-epoxy-5,8-dihydroxy-5,6,7,8-tetrahydrochromone | C_18_H_18_O_6_ | 330.1103 | 330.1088 | -4.6 | 2 |  |  | √ |  | √ | √ |
| 11.44 | 5,6,7,8-diepoxy-2-[2-(4-hydroxy)ethyl]-5,6,7,8-tetrahydrochromone | C_17_H_14_O_5_ | 298.0841 | 298.0826 | -5.1 | 3 |  | √ | √ | √ | √ | √ |
| 11.99 | 5,6,7,8-diepoxy-2-[2-(3-hydroxy-4-methoxyphenyl)ethyl]-5,6,7,8-tetrahydrochromone(Oxidoagarochromone C) | C_18_H_16_O_6_ | 328.0947 | 328.0928 | -5.7 | 2 |  | √ |  | √ | √ | √ |
| 12.12 | 2-[2-(4'-hydroxy)ethyl]-6,7-epoxy-5,8-dihydroxy-5,6,7,8-tetrahydrochromone | C_17_H_16_O_6_ | 316.0947 | 316.0933 | -4.5 | 3 |  |  | √ |  |  |  |
| 12.86 | 5,6,7,8-diepoxy-2-[2-(3-hydroxy-4-methoxyphenyl)ethyl]-5,6,7,8-tetrahydrochromone(Oxidoagarochromone C) | C_18_H_16_O_6_ | 328.0947 | 328.0933 | -4.2 | 4 |  | √ | √ | √ | √ | √ |
| 14.01 | 2-(2-phenylethyl)-6,7-epoxy-5,8-dihydroxy-5,6,7,8-tetrahydrochromone | C_17_H_16_O_5_ | 300.0998 | 300.0984 | -4.7 | 5 | √ |  | √ | √ | √ | √ |
| 14.58 | 5,6,7,8-diepoxy-2-[2-(3-hydroxy-4-methoxyphenyl)ethyl]-5,6,7,8-tetrahydrochromone(Oxidoagarochromone C) | C_18_H_16_O_6_ | 328.0947 | 328.0931 | -4.7 | 2 |  |  | √ |  |  |  |
| 14.68 | 2-[2-(3'-hydroxy-4'-methoxyphenyl)ethyl]-7,8-epoxy-5-methoxy-6-hydroxy-5,6,7,8-tetrahydrochromone | C_19_H_20_O_7_ | 360.1209 | 360.1187 | -6.0 | 2 | √ |  |  | √ | √ | √ |
| 16.55 | 8-chloro-5,6,7-trihydroxy-5,6,7,8-tetrahydro-2-[2-(4'-methoxyphenyl)ethyl]chromone | C_18_H_19_ClO_6_ | 366.0870 | 366.0853 | -4.8 | 4 |  | √ | √ | √ | √ | √ |
| 16.56 | 2-[2-(4'-methoxyphenyl)ethyl]-6,7-epoxy-5,8-dihydroxy-5,6,7,8-tetrahydrochromone(isomer) | C_18_H_18_O_6_ | 330.11034 | 330.1082 | -6.4 | 4 |  |  |  | √ |  |  |
| 16.99 | 8-chloro-5,6,7-trihydroxy-5,6,7,8-tetrahydro-2-(2-phenylethyl)chromone | C_17_H_17_ClO_5_ | 336.0765 | 336.0749 | -4.5 | 5 |  |  |  |  | √ | √ |
| 17.9 | 2-[2-(4'-methoxyphenyl)ethyl]-6,7-epoxy-5,8-dihydroxy-5,6,7,8-tetrahydrochromone | C_18_H_18_O_6_ | 330.1103 | 330.1089 | -4.4 | 4 |  | √ | √ | √ | √ | √ |
| 18.35 | 2-(2-phenylethyl)-6,7-epoxy-5,8-dihydroxy-5,6,7,8-tetrahydrochromone | C_17_H_16_O_5_ | 300.0998 | 300.0986 | -3.9 | 5 | √ | √ | √ | √ | √ | √ |
| 19.01 | 5,6,7,8-diepoxy-2-[2-(4-methoxyphenyl)ethyl]-5,6,7,8-tetrahydrochromone(Oxidoagarochromone B) | C_18_H_16_O_5_ | 312.0998 | 312.0984 | -4.5 | 4 |  |  | √ | √ | √ | √ |
| 19.98 | 6-hydroxy-2-[2-(3'-hydroxy-4'-methoxyphenyl)ethyl]chromone | C_18_H_16_O_5_ | 312.0998 | 312.0979 | -5.9 | 2 |  |  |  |  | √ |  |
| 21.58 | 2-[2-hydroxy--2-(4'-hydroxyphenyl)ethyl]chromone | C_17_H_14_O_4_ | 282.0892 | 282.0877 | -5.2 | 1 |  | √ | √ | √ | √ | √ |
| 22.17 | 5,6,7,8-diepoxy-2-[2-(4-methoxyphenyl)ethyl]-5,6,7,8-tetrahydrochromone(Oxidoagarochromone B) | C_18_H_16_O_5_ | 312.0998 | 312.0984 | -4.4 | 4 | √ | √ | √ | √ | √ | √ |
| 22.74 | 5,6,7,8-diepoxy-2-(2-phenylethyl)-5,6,7,8-tetrahydrochromone(Oxidoagarochromone A) | C_17_H_14_O_4_ | 282.0892 | 282.0877 | -5.4 | 4 | √ | √ | √ | √ | √ | √ |
| 23.4 | 6,7-dihydroxy-2-[2-(4'-methoxyphenyl)ethyl]chromone | C_18_H_16_O_5_ | 312.0998 | 312.0983 | -4.6 | 2 |  |  | √ |  |  | √ |
| 24.23 | 5,6,7,8-diepoxy-2-(2-phenylethyl)-5,6,7,8-tetrahydrochromone(Oxidoagarochromone A) | C_17_H_14_O_4_ | 282.0892 | 282.0874 | -6.6 | 2 | √ | √ |  | √ | √ | √ |
| 27.01 | S1-1 | C_35_H_30_O_10_ | 610.1839 | 610.1841 | 0.3 | 1 |  |  |  | √ | √ | √ |
| 27.44 | 6-hydroxy-7-methoxy-2-[2-(4'-methoxyphenyl)ethyl]chromone | C_19_H_18_O_5_ | 326.1154 | 326.1140 | -4.4 | 2 |  | √ |  |  | √ |  |
| 27.55 | D5-3 | C_34_H_30_O_9_ | 582.1889 | 582.1887 | -0.5 | 0 |  |  |  | √ |  |  |
| 27.83 | 6-methoxy-2-[2-(3'-hydroxy-4'-methoxyphenyl)ethyl]chromone | C_19_H_18_O_5_ | 326.1154 | 326.1142 | -3.6 | 3 |  |  |  | √ | √ | √ |
| 28.45 | 6-hydroxy-7-methoxy-2-(2-phenylethyl)chromone | C_18_H_16_O_4_ | 296.1049 | 296.1034 | -5.0 | 5 |  |  |  | √ | √ | √ |
| 29.47 | 6,7-dihydroxy-2-[2-(4'-hydroxy-3'-methoxyphenyl)ethyl]chromone(isomer) | C_18_H_16_O_6_ | 328.0947 | 328.0927 | -1.3 | 0 |  | √ |  |  | √ | √ |
| 30.15 | 6-hydroxy-2-[2-(4'-methoxyphenyl)ethyl]chromone | C_18_H_16_O_4_ | 296.1049 | 296.1033 | -5.4 | 2 |  | √ |  | √ | √ | √ |
| 31.27 | 6-hydroxy-2-(2-phenylethyl)chromone | C_17_H_14_O_3_ | 266.0943 | 266.0926 | -6.4 | 5 | √ | √ |  | √ | √ | √ |
| 33.29 | 6,7-dimethoxy-2-[2-(4'-methoxyphenyl)ethyl]chromone | C_20_H_20_O_5_ | 340.1311 | 340.1298 | -3.8 | 2 | √ | √ | √ | √ | √ | √ |
| 34.42 | 6,7-dimethoxy-2-(2-phenylethyl)chromone | C_19_H_18_O_4_ | 310.1205 | 310.1190 | -4.8 | 4 | √ | √ | √ | √ | √ | √ |
| 35.13 | S8-1 | C_34_H_28_O_8_ | 564.1784 | 564.1786 | 0.4 | 0 | √ |  |  |  |  |  |
| 35.37 | S8-2 | C_35_H_30_O_9_ | 594.1890 | 594.1892 | 0.4 | 0 |  | √ |  | √ |  | √ |
| 36.05 | D1-4 | C_34_H_30_O_8_ | 566.1941 | 566.1934 | -1.1 | 0 |  | √ |  | √ | √ | √ |
| 36.43 | D1-3 | C_35_H_32_O_9_ | 596.2046 | 596.2036 | -1.7 | 0 |  | √ |  | √ |  | √ |
| 37.46 | D1-4 | C_34_H_30_O_8_ | 566.1941 | 566.1930 | -1.9 | 1 |  | √ |  | √ | √ |  |
| 38.25 | 2-[2-(4'-methoxyphenyl)ethyl]chromone | C_18_H_16_O_3_ | 280.1099 | 280.1083 | -5.9 | 2 | √ |  | √ | √ |  |  |
| 39.56 | 2-(2-phenylethyl)chromone | C_17_H_14_O_2_ | 250.1056 | 251.0056 | -2.9 | 1 | √ | √ |  | √ | √ | √ |
| 40.58 | 5-chloro-6-hydroxy-2-(2-phenylethyl)chromone | C_17_H_13_ClO_3_ | 300.0553 | 300.0536 | -5.7 | 0 |  |  |  | √ |  |  |
| 40.68 | 6-methoxy-2-[2-(4'-methoxyphenyl)ethyl]chromone | C_19_H_18_O_4_ | 310.1205 | 310.1191 | -4.6 | 5 | √ | √ |  | √ | √ |  |
| 41.87 | 6-methoxy-2-(2-phenylethyl)chromone | C_18_H_16_O_3_ | 280.1099 | 280.1084 | -5.4 | 5 | √ | √ | √ | √ | √ |  |
| 43.65 | 6-hydroxy-7-methoxy-2-[2-(4'-methoxyphenyl)ethyl]chromone | C_19_H_18_O_5_ | 326.1154 | 326.1136 | -5.6 | 2 | √ | √ | √ |  |  |  |
| 45.17 | 6-hydroxy-7-methoxy-2-(2-phenylethyl)chromone | C_18_H_16_O_4_ | 296.1049 | 296.1032 | -5.7 | 4 |  |  |  | √ |  | √ |
| 46.26 | S3-2 | C_34_H_28_O_7_ | 548.1835 | 548.1830 | -0.9 | 1 | √ | √ |  | √ |  | √ |
| 47.43 | S-2 | C_35_H_30_O_8_ | 578.1941 | 578.1941 | 0.0 | 1 | √ | √ |  | √ |  | √ |
| 47.89 | D4-4 | C_34_H_28_O_7_ | 548.1835 | 548.1828 | -1.4 | 0 | √ | √ | √ | √ |  | √ |
| 62.08 | 5-hydroxy-6.7-dimethoxy-2-[2-(3'-hydroxy-4-methoxyphenyl)ethyl]chromoe | C_20_H_20_O_7_ | 372.1209 | 372.1191 | -4.9 | 0 |  |  |  |  |  | √ |

**Supplementary Table S3****.** Information of common peaks of 2M

| **NO.** | **t_R_ (min)** | **Formula** | **λ_max_** | **[M+H]^+^**  **measured** | **MS/MS (*m/z*)** | **A ring** | **Bring** | **Type** | **Proposed compound** |
| --- | --- | --- | --- | --- | --- | --- | --- | --- | --- |
| 1 | 7.94 | C_17_H_18_O_6_ | 253.2 | 319.1187 | 301.1062,283.0950,255.1000,227.1050,91.0570,164.0452 | 4OH |  | THPECs | Agarotetrol |
| 2 | 8.88 | C_17_H_18_O_6_ | 253.2 | 319.1187 | 301.1062,283.0950,255.0997,227.1050,91.0536,164.0454 | 4OH |  | THPECs | Agaroretrol (isomer) |
| 3 | 9.33 | C_17_H_18_O_6_ | 253.2 | 319.1161 | 301.1064,283.0951,255.1000,227.1052,91.0536,164.0454 | 4OH |  | THPECs | Agaroretrol (isomer) |
| 4 | 14.07 | C_17_H_16_O_5_ | 252 | 301.1081 | 283.0954,255.1001,227.1053,91.0530,164.0454 | -O-,2OH |  | EPECs | 2-(2-phenylethyl)-6,7-epoxy-5,8-dihydroxy-5,6,7,8-tetrahydrochromone(isomer) |
| 5 | 14.64 | C_19_H_20_O_7_ | 252 | 361.1284 | 224.1241,317.1006,137.0587 | -O-,OH，OCH_3_ | OH,OCH_3_ | EPECs | 2-[2-(3'-hydroxy-4'-methoxyphenyl)ethyl]-7,8-epoxy-5-methoxy-6-hydroxy-5,6,7,8-tetrahydrochromone(isomer) |
| 6 | 18.34 | C_17_H_16_O_5_ | 253.2 | 301.1060 | 283.0954,255.1001,192.0399,227.1053,91.0530,164.0454 | -O-,2OH |  | EPECs | 2-(2-phenylethyl)-6,7-epoxy-5,8-dihydroxy-5,6,7,8-tetrahydrochromone(isomer) |
| 7 | 22.13 | C_18_H_16_O_5_ | 224,8,253.2 | 313.1069 | 285.1104,192.0401,121.0635,91.0530 | 2-O- | OCH_3_ | DEPECs | oxidoagarochromone B |
| 8 | 22.72 | C_17_H_14_O_4_ | 222.4,253.2 | 283.0970 | 255.0970,227.1056,192.0406,91.0533 | 2-O- |  | DEPECs | oxidoagarochromone A |
| 9 | 24.20 | C_17_H_14_O_4_ | 235.4,284.1 | 283.0970 | 255.1000,192.0403,91.0530 | 2-O- |  | DEPECs | oxidoagarochromone A(isomer) |
| 10 | 31.26 | C_17_H_14_O_3_ | 239.0,327.0 | 267.1009 | 176.0457,137.0221,91.0533 | OH |  | FTPECs | 6-hydroxy-2-(2-phenylethyl)chromone(isomer) |
| 11 | 33.27 | C_20_H_20_O_5_ | 241.3,317.5 | 341.1393 | 220.0176,121.0637 | 2OCH_3_ | OCH_3_ | FTPECs | 6,7-dimethoxy-2-[2-(4'-methoxyphenyl)ethyl]chromone(isomer) |
| 12 | 34.40 | C_19_H_18_O_4_ | 236.6,279.3 | 311.1284 | 220.0720,205.0485,91.0532 | 2OCH_3_ |  | FTPECs | 6,7-dimethoxy-2-(2-phenylethyl)chromone(isomer) |
| 13 | 35.28 | C_35_H_30_O_9_ | 243.7 | 565.1860 | 547.1750,474.1313,283.0970,91.0530 | FTPECs | FTPECs | S-Dimers | S8-2 |
| 14 | 38.27 | C_18_H_16_O_3_ | 243.7 | 281.1174 | 121.0634 |  | OCH_3_ | FTPECs | 2-[2-(4'-methoxyphenyl)ethyl]chromone(isomer) |
| 15 | 39.53 | C_17_H_14_O_2_ | 229.5,297.2 | 251.1070 | 160.0504,173.0582,121.0270,91.0531 |  |  | FTPECs | 2-(2-phenylethyl)chromone |
| 16 | 40.68 | C_19_H_18_O_4_ | 241.3,327.0 | 311.1280 | 190.0614,121.0645,91.0536 | OCH_3_ | OCH_3_ | FTPECs | 6-methoxy-2-[2-(4'-methoxyphenyl)ethyl]chromone(isomer) |
| 17 | 41.81 | C_18_H_16_O_3_ | 240.1,321.1 | 281.1174 | 190.0614,151.0377,91.0536, | OCH_3_ |  | FTPECs | 6-methoxy-2-(2-phenylethyl)chromone(isomer) |
| 18 | 42.94 |  | 253.2,344.6 | 255.1324 |  |  |  |  | - |
| 19 | 43.58 | C_19_H_18_O_5_ | 242.5,336.5 | 327.1212 | 121.063 | OH,OCH_3_ | OCH_3_ | FTPECs | 6-hydroxy-7-methoxy-2-[2-(4'-methoxyphenyl)ethyl]chromone(isomer) |
| 20 | 46.24 | C_34_H_28_O_7_ | 249.6,344.6 | 549.1886 | 531.1799,267.1002,91.0530 | FTPECs | FTPECs | S-Dimers | D4-4 |
| 21 | 47.40 | C_35_H_30_O_8_ | 247.3,331.8 | 579.2021 | 488.1468,458.1468,121.0633,91.0529 | FTPECs | EPECs | D-Dimers | S-2 |
| 22 | 48.86 | C_34_H_28_O_7_ | 244.9,329.4 | 549.1913 | 531.1796,458.1357,283.0950 | FTPECs | FTPECs | S-Dimers | S3-2 |

**Supplementary Table S4.** Information of common peaks of 4M

| **NO.** | **t_R_ (min)** | **Formula** | **λ_max_** | **[M+H]^+^**  **measured** | **MS/MS (*m/z*)** | **A ring** | **Bring** | **Type** | **Proposed compound** |
| --- | --- | --- | --- | --- | --- | --- | --- | --- | --- |
| 1 | 6.26 | C_17_H_18_O_7_ | 250.8,350.4 | 335.1115 | 301.1060,271.0970,243.1021,107.0487 | 4OH | OH | THPECs | 2-[2-(4'-hydroxy)ethyl]-5,6,7,8-tetrahydroxy-5,6,7,8-tetrahydrochromone(isomer) |
| 2 | 7.94 | C_17_H_18_O_6_ | 210.6,250.8 | 319.1187 | 301.1062,283.0950,255.1000,227.1050,91.0570,164.0452 | 4OH |  | THPECs | Agarotetrol |
| 3 | 8.88 | C_17_H_18_O_6_ | 253.2,325.8 | 319.1187 | 301.1062,283.0950,255.0997,227.1050,91.0536,164.0454 | 4OH |  | THPECs | Agaroretrol(isomer) |
| 4 | 9.33 | C_17_H_18_O_6_ | 250.8,325.8 | 319.1161 | 301.1064,283.0951,255.1000,227.1052,91.0536,164.0454 | 4OH |  | THPECs | Agaroretrol(isomer) |
| 5 | 11.51 | C_17_H_14_O_5_ | 250.8,325.8 | 299.0896 | 271.0973,192.0404,107.0481 | 2-O- | OH | DEPECs | 5,6,7,8-diepoxy-2-[2-(4-hydroxy)ethyl]-5,6,7,8-tetrahydrochromone(isomer) |
| 6 | 12.05 | C_18_H_16_O_6_ | 310.3,325.8 | 329.1012 | 301.1397,137.0585 | 2-O- | OH,OCH_3_ | DEPECs | Oxidoagarochromone C(isomer) |
| 7 | 12.89 | C_18_H_16_O_6_ | 220.1,255.5 | 329.1016 | 301.1016,192.0407,137.0589 | 2-O- | OH,OCH_3_ | DEPECs | Oxidoagarochromone C(isomer) |
| 8 | 16.56 | C_18_H_19_ClO_6_ | 253.2,317.5 | 367.0927 | 349.0841,331.0737,313.1058,121.0630 | 3OH,Cl | OCH_3_ | THPECs | 8-chloro-5,6,7-trihydroxy-5,6,7,8-tetrahydro-2-[2-(4'-methoxyphenyl)ethyl]chromone(isomer) |
| 9 | 17.91 | C_18_H_18_O_6_ | 228.3,249.6 | 331.1192 | 331.1192,313.1075,121.0639,91.0533 | -O-,2OH |  | EPECs | 2-[2-(4'-methoxyphenyl)ethyl]-6,7-epoxy-5,8-dihydroxy-5,6,7,8-tetrahydrochromone(isomer) |
| 10 | 18.34 | C_17_H_16_O_5_ | 210.6,253.2 | 301.1060 | 283.0954,255.1001,192.0399,227.1053,91.0530,164.0454 | -O-,2OH |  | EPECs | 2-(2-phenylethyl)-6,7-epoxy-5,8-dihydroxy-5,6,7,8-tetrahydrochromone(isomer) |
| 11 | 21.58 | C_17_H_14_O_4_ | 222.4,253.2 | 283.0970 | 255.0970,227.1056,192.0406,91.0533 | 2-O- |  | DEPECs | 2-[2-hydroxy--2-(4'-hydroxyphenyl)ethyl]chromone |
| 12 | 22.13 | C_18_H_16_O_5_ | 226.0,254.4 | 313.1069 | 285.1104,192.0401,121.0635,91.0530 | 2-O- | OCH_3_ | DEPECs | Oxidoagarochromone B |
| 13 | 22.72 | C_17_H_14_O_4_ | 226.0,250.8 | 283.0970 | 255.0970,227.1056,192.0406,91.0533 | 2-O- |  | DEPECs | Oxidoagarochromone A |
| 14 | 24.20 | C_17_H_14_O_4_ | 236.6,282.9 | 283.0970 | 255.1000,192.0403,91.0530 | 2-O- |  | DEPECs | Oxidoagarochromone A(isomer) |
| 15 | 27.44 | C_19_H_18_O_5_ | 236.6,319.9 | 327.1212 | 121.0638,91.0533 | OH,OCH_3_ | OCH_3_ | FTPECs | 6-hydroxy-7-methoxy-2-[2-(4'-methoxyphenyl)ethyl]chromone(isomer) |
| 16 | 29.27 | C_18_H_16_O_6_ | 247.3,380.5 | 329.1042 | 137.0583 | 2OH | OH,OCH_3_ | FTPECS | 6,7-dihydroxy-2-[2-(4'-hydroxy-3'-methoxyphenyl)ethyl]chromone(isomer) |
| 17 | 30.10 | C_18_H_16_O_4_ | 240.1,325.8 | 297.1107 | 121.0638 | OH | OCH_3_ | FTPECs | 6-hydroxy-2-[2-(4'-methoxyphenyl)ethyl]chromone(isomer) |
| 18 | 31.26 | C_17_H_14_O_3_ | 240.1,325.8 | 267.1009 | 176.0457,137.0221,91.0533 | OH |  | FTPECs | 6-hydroxy-2-(2-phenylethyl)chromone(isomer) |
| 19 | 33.27 | C_20_H_20_O_5_ | 239.0,319.9 | 341.1393 | 220.0176,121.0637 | 2OCH_3_ | OCH_3_ | FTPECs | 6,7-dimethoxy-2-[2-(4'-methoxyphenyl)ethyl]chromone(isomer) |
| 20 | 34.40 | C_19_H_18_O_4_ | 235.4,279.3 | 311.1284 | 220.0720,205.0485,91.0532 | 2OCH_3_ |  | FTPECs | 6,7-dimethoxy-2-(2-phenylethyl)chromone(isomer) |
| 21 | 35.28 | C_35_H_30_O_9_ | 242.5,319.9 | 595.1969 | 565.1846,547.1718,313.1053,283.0935,121.0622,91.0536 | FTPECs | FTPECs | S-Dimers | S8-2 |
| 22 | 36.01 | C_34_H_30_O_8_ | 243.7,321.1 | 567.2018 | 549.1906,531.1809,283.0976,91.0532 | FTPECs | FTPEC | S-Dimers | S4-1 |
| 23 | 36.32 | C_35_H_32_O_9_ | 243.7,318.7 | 597.2097 | 579.2006,121.0622 | THPECs | FTPECs | D-Dimers | D1-3 |
| 24 | 38.30 | C_34_H_30_O_8_ | 242.5,319.9 | 595.1962 | 547.1736,121.0634,283.0941,121.0630,91.0529 | FTPECs | FTPECs | S-Dimers | D1-4 |
| 25 | 39.45 | C_17_H_14_O_2_ | 229.5,296.0 | 251.1056 | 160.0504,173.0582,121.0270,91.0531 |  |  | FTPECs | 2-(2-phenylethyl)chromone |
| 26 | 40.60 | C_19_H_18_O_4_ | 240.1,322.3 | 311.1280 | 190.0614,121.0645,91.0536 | OCH_3_ | OCH_3_ | FTPECs | 6-methoxy-2-[2-(4'-methoxyphenyl)ethyl]chromone(isomer) |
| 27 | 41.78 | C_18_H_16_O_3_ | 240.1,321.1 | 281.1174 | 190.0614,151.0377,91.0536 | OCH_3_ |  | FTPECs | 6-methoxy-2-(2-phenylethyl)chromone(isomer) |
| 28 | 43.00 |  | 252.0,318.7 | 255.1324 |  |  |  | 未鉴定 |  |
| 29 | 43.58 | C_19_H_18_O_5_ | 239.0,319.9 | 327.1212 | 121.063 | OH,OCH_3_ | OCH_3_ | FTPECs | 6-hydroxy-7-methoxy-2-[2-(4'-methoxyphenyl)ethyl]chromone(isomer) |
| 30 | 46.20 | C_34_H_28_O_7_ | 248.4,344.6 | 549.1886 | 531.1799,267.1002,91.0530 | FTPECs | FTPECs | S-Dimers | D4-4 |
| 31 | 47.40 | C_35_H_30_O_8_ | 246.1,333.0 | 579.2021 | 488.1468,458.1468,121.0633,91.0529 | FTPECs | EPECs | D-Dimers | S-2 |
| 32 | 48.84 | C_34_H_28_O_7_ | 246.1,325.8 | 549.1913 | 531.1796,458.1357,283.0950 | FTPECs | FTPECs | S-Dimers | S3-2 |
| 33 | 49.95 |  | 252.0,325.8 | 304.2994 |  |  |  |  | - |

**Supplementary Table S5.** Information of common peaks of 6M

| **NO.** | **t_R_ (min)** | **Formula** | **λ_max_** | **[M+H]^+^**  **measured** | **MS/MS (*m/z*)** | **A ring** | **Bring** | **Type** | **Proposed compound** |
| --- | --- | --- | --- | --- | --- | --- | --- | --- | --- |
| 1 | 4.99 | C_18_H_20_O_8_ | 211.8,354.0 | 365.1222 | 347.1115,329.1010,301.1034,137.0581 | 4OH | OH,OCH_3_ | THPECs | 2-[2-(3-hydroxy-4-methoxyphenyl)ethyl]-5,6,7,8-tetrahydroxy-5,6,7,8-tetrahydrochromone (isomer) |
| 2 | 5.35 | C_18_H_20_O_8_ | 210.6,329.4 | 365.1222 | 347.1100,329.1010,301.1034,137.0580 | 4OH | OH,OCH_3_ | THPECs | 2-[2-(3-hydroxy-4-methoxyphenyl)ethyl]-5,6,7,8-tetrahydroxy-5,6,7,8-tetrahydrochromone (isomer) |
| 3 | 7.67 | C_18_H_18_O_7_ | 211.8,253.2 | 347.1117 | 329.1010,301.1056,122.0346,173.0851 | O,2OH | OH,OCH_3_ | EPECs | 2-[2-(3'-hydroxy-4'-methoxyphenyl)ethyl]-6,7-epoxy-5,8-dihydroxy-5,6,7,8-tetrahydrochromone (isomer) |
| 4 | 7.94 | C_17_H_18_O_6_ | 252.0,329.4 | 319.1187 | 301.1062,283.0950,255.1000,227.1050,91.0570,164.0452 | 4OH |  | THPECs | Agarotetrol |
| 5 | 8.88 | C_17_H_18_O_6_ | 211.8,253.2 | 319.1187 | 301.1062,283.0950,255.0997,227.1050,91.0536,164.0454 | 4OH |  | THPECs | Agarotetrol (isomer) |
| 6 | 9.33 | C_17_H_18_O_6_ | 211.8,253.2 | 319.1161 | 301.1064,283.0951,255.1000,227.1052,91.0536,164.0454 | 4OH |  | THPECs | Agarotetrol (isomer) |
| 7 | 9.99 | C_18_H_18_O_7_ | 210.6,254.4 | 347.1118 | 329.1011,301.1053,137.0580,122.0354 | -O-,2OH | OH,OCH_3_ | EPECs | 2-[2-(3'-hydroxy-4'-methoxyphenyl)ethyl]-6,7-epoxy-5,8-dihydroxy-5,6,7,8-tetrahydrochromone (isomer) |
| 8 | 10.46 | C_18_H_18_O_6_ | 214.2,254.4 | 331.1146 | 313.1053,137.0597,122.0346 | -O-,OH | OH,OCH_3_ | EPECs | 2-[2-(4'-methoxyphenyl)ethyl]-6,7-epoxy-5,8-dihydroxy-5,6,7,8-tetrahydrochromone |
| 9 | 11.51 | C_17_H_14_O_5_ | 216.5,253.2 | 299.0896 | 271.0973,192.0404,107.0481 | 2-O- | OH | DEPECs | 5,6,7,8-diepoxy-2-[2-(4-hydroxy)ethyl]-5,6,7,8-tetrahydrochromone (isomer) |
| 10 | 12.05 | C_18_H_16_O_6_ | 216.5,253.2 | 329.1012 | 301.1397,137.0585 | 2-O- | OH,OCH_3_ | DEPECs | Oxidoagarochromone C(isomer) |
| 11 | 12.89 | C_18_H_16_O_6_ | 226.0,255.5 | 329.1016 | 301.1016,192.0407,137.0589 | 2-O- | OH,OCH_3_ | DEPECs | Oxidoagarochromone C(isomer) |
| 12 | 13.99 | C_17_H_16_O_5_ | 222.4,253.2, | 301.1081 | 283.0954,255.1001,227.1053,91.0530,164.0454 | -O-,2OH |  | EPECs | 2-(2-phenylethyl)-6,7-epoxy-5,8-dihydroxy-5,6,7,8-tetrahydrochromone |
| 13 | 14.56 | C_18_H_16_O_6_ | 229.5,281.7 | 329.1004 | 301.1407,192.0399,164.0444,137.0580 | 2-O- | OCH_3_ | DEPECs | (Oxidoagarochromone C) (isomer) |
| 14 | 16.56 | C_18_H_19_ClO_6_ | 237.8,252.0,318.7 | 367.0927 | 349.0841,331.0737,313.1058,121.0630 | 3OH,Cl | OCH_3_ | THPECs | 8-chloro-5,6,7-trihydroxy-5,6,7,8-tetrahydro-2-[2-(4'-methoxyphenyl)ethyl]chromone(isomer) |
| 15 | 17.91 | C_18_H_18_O_6_ | 226.0,252.0 | 331.1192 | 331.1192,313.1075,121.0639,91.0533 | -O-,2OH |  | EPECs | 2-[2-(4'-methoxyphenyl)ethyl]-6,7-epoxy-5,8-dihydroxy-5,6,7,8-tetrahydrochromone (isomer) |
| 16 | 18.34 | C_17_H_16_O_5_ | 252.0,385.3 | 301.1060 | 283.0954,255.1001,192.0399,227.1053,91.0530,164.0454 | -O-,2OH |  | EPECs | 2-(2-phenylethyl)-6,7-epoxy-5,8-dihydroxy-5,6,7,8-tetrahydrochromone(isomer) |
| 17 | 19.03 | C_18_H_16_O_5_ | 226.0,319.9 | 313.1053 | 285.1111,122.0345,121.0632 | -O-,2OH | OCH_3_ | EPECs | 2-[2-(4'-methoxyphenyl)ethyl]-6,7-epoxy-5,8-dihydroxy-5,6,7,8-tetrahydrochromone(isomer) |
| 18 | 21.58 | C_17_H_14_O_4_ | 222.4,253.2 | 283.0970 | 255.0970,227.1056,192.0406,91.0533 | 2-O- |  | DEPECs | 2-[2-hydroxy--2-(4'-hydroxyphenyl)ethyl]chromone |
| 19 | 22.13 | C_18_H_16_O_5_ | 224.8,255.5 | 313.1069 | 285.1104,192.0401,121.0635,91.0530 | 2-O- | OCH_3_ | DEPECs | Oxidoagarochromone B |
| 20 | 22.72 | C_17_H_14_O_4_ | 217.7,253.2 | 283.0970 | 255.0970,227.1056,192.0406,91.0533 | 2-O- |  | DEPECs | Oxidoagarochromone A |
| 21 | 23.36 | C_18_H_16_O_5_ | 233.1,322.3 | 313.1076 | 285.1092，121.0633,192.0633,91.0528 | 2-O- | OCH_3_ | DEPECs | Oxidoagarochromone B(isomer) |
| 22 | 33.27 | C_20_H_20_O_5_ | 239.0,316.3 | 341.1393 | 220.0176,121.0637 | 2OCH3 | OCH_3_ | FTPECs | 6,7-dimethoxy-2-[2-(4'-methoxyphenyl)ethyl]chromone(isomer) |
| 23 | 34.40 | C_19_H_18_O_4_ | 236.6,278.1 | 311.1284 | 220.0720,205.0485,91.0532 | 2OCH3 |  | FTPECs | 6,7-dimethoxy-2-(2-phenylethyl)chromone(isomer) |
| 24 | 38.15 | C_18_H_16_O_3_ | 242.5,329.4 | 281.1155 | 121.0630 |  |  | FTPECs | 2-[2-(4'-methoxyphenyl)ethyl]chromone(isomer) |
| 25 | 41.78 | C_18_H_16_O_3_ | 241.3,321.1 | 281.1174 | 190.0614,151.0377,91.0536 | OH |  | FTPECs | 6-methoxy-2-(2-phenylethyl)chromone(isomer) |
| 26 | 43.00 |  | 253.2,317.5 | 255.1324 |  |  |  |  | - |
| 27 | 43.58 | C_19_H_18_O_5_ | 236.6,329.4 | 327.1212 | 121.063 | OH, OCH_3_ | OCH_3_ | FTPECs | 6-hydroxy-7-methoxy-2-[2-(4'-methoxyphenyl)ethyl]chromone(isomer) |
| 28 | 48.84 | C_34_H_28_O_7_ | 248.4,333.0 | 549.1913 | 531.1796,458.1357,283.0950 | FTPECs | FTPECs | S-Dimers | S3-2 |
| 29 | 49.95 |  | 252.0,317.5 | 304.2994 |  |  |  |  | - |

**Supplementary Table S6.** Information of common peaks of 8M

| **NO.** | **t_R_ (min)** | **Formula** | **λ_max_** | **[M+H]^+^**  **measured** | **MS/MS (*m/z*)** | **A ring** | **Bring** | **Type** | **Proposed compound** |
| --- | --- | --- | --- | --- | --- | --- | --- | --- | --- |
| 1 | 4.99 | C_18_H_20_O_8_ | 253.2,315.1 | 365.1222 | 347.1115,329.1010,301.1034,137.0581 | 4OH | OH,OCH_3_ | THPECs | 2-[2-(3-hydroxy-4-methoxyphenyl)ethyl]-5,6,7,8-tetrahydroxy-5,6,7,8-tetrahydrochromone(isomer) |
| 2 | 5.35 | C_18_H_20_O_8_ | 324.6,350.0 | 365.1222 | 347.1100,329.1010,301.1034,137.0580 | 4OH | OH,OCH_3_ | THPECs | 2-[2-(3-hydroxy-4-methoxyphenyl)ethyl]-5,6,7,8-tetrahydroxy-5,6,7,8-tetrahydrochromone(isomer) |
| 3 | 7.67 | C_18_H_18_O_7_ | 323.5,342.3 | 347.1117 | 329.1010,301.1056,122.0346,173.0851 | O,2OH | OH,OCH_3_ | EPECs | 2-[2-(3'-hydroxy-4'-methoxyphenyl)ethyl]-6,7-epoxy-5,8-dihydroxy-5,6,7,8-tetrahydrochromone(isomer) |
| 4 | 7.94 | C_17_H_18_O_6_ | 210.6,252.0 | 319.1187 | 301.1062,283.0950,255.1000,227.1050,91.0570,164.0452 | 4OH |  | THPECs | Agarotetrol |
| 5 | 8.50 | C_17_H_18_O_6_ | 315.1,350.4 | 319.1187 | 301.1080,283.0970,255.1030,227.1049,164.0452,91.0542 | 4OH |  | THPECs | 5.6.7-trihydroxy-5.6.7.8-tetrahydro-2-[2-(3'-hydroxyphenyl)ethyl]chromone |
| 6 | 8.88 | C_17_H_18_O_6_ | 253.2,331.8 | 319.1187 | 301.1062,283.0950,255.0997,227.1050,91.0536,164.0454 | 4OH |  | THPECs | Agarotetrol(isomer) |
| 7 | 9.33 | C_17_H_18_O_6_ | 211.0,253. | 319.1161 | 301.1064,283.0951,255.1000,227.1052,91.0536,164.0454 | 4OH |  | THPECs | Agarotetrol(isomer) |
| 8 | 9.99 | C_18_H_18_O_7_ | 253.2,315.1 | 347.1118 | 329.1011,301.1053,137.0580,122.0354 | O,2OH | OH,OCH_3_ | EPECs | 2-[2-(3'-hydroxy-4'-methoxyphenyl)ethyl]-6,7-epoxy-5,8-dihydroxy-5,6,7,8-tetrahydrochromone(isomer) |
| 9 | 11.51 | C_17_H_14_O_5_ | 250.8,335.4 | 299.0896 | 271.0973,192.0404,107.0481 | 2O | OH | DEPECs | 5,6,7,8-diepoxy-2-[2-(4-hydroxy)ethyl]-5,6,7,8-tetrahydrochromone(isomer) |
| 10 | 12.05 | C_18_H_16_O_6_ | 317.5,338.8 | 329.1012 | 301.1397,137.0585 | 2O | OH,OCH_3_ | DEPECs | Oxidoagarochromone C(isomer) |
| 11 | 12.89 | C_18_H_16_O_6_ | 220.1,253.2 | 329.1016 | 301.1016,192.0407,137.0589 | 2O | OH,OCH_3_ | DEPECs | Oxidoagarochromone C(isomer) |
| 12 | 13.99 | C_17_H_16_O_5_ | 211.8,249.6, | 301.1081 | 283.0954,255.1001,227.1053,91.0530,164.0454 | O,2OH |  | EPECs | 2-(2-phenylethyl)-6,7-epoxy-5,8-dihydroxy-5,6,7,8-tetrahydrochromone(isomer) |
| 13 | 14.64 | C_19_H_20_O_7_ | 323.5,364.9 | 361.1284 | 224.1241,317.1006,137.0587 | O,OH,OCH_3_ | OH,OCH_3_ | EPECs | 2-[2-(3'-hydroxy-4'-methoxyphenyl)ethyl]-7,8-epoxy-5-methoxy-6-hydroxy-5,6,7,8-tetrahydrochromone(isomer) |
| 14 | 16.55 | C_18_H_19_ClO_6_ | 215.3,253.2 | 367.0927 | 349.0841,331.0737,313.1058,121.0630 | 3OH,Cl | OCH_3_ | THPECs | 8-chloro-5,6,7-trihydroxy-5,6,7,8-tetrahydro-2-[2-(4'-methoxyphenyl)ethyl]chromone(isomer) |
| 15 | 16.56 | C_18_H_18_O_6_ | 224.8,317.5 | 331.1192 | 285.1091,192.0399,121.0622,91.0529 | O,2OH | OCH_3_ | EPECs | 2-[2-(4'-methoxyphenyl)ethyl]-6,7-epoxy-5,8-dihydroxy-5,6,7,8-tetrahydrochromone(isomer) |
| 16 | 17.91 | C_18_H_18_O_6_ | 224.8,255.5 | 331.1192 | 331.1192,313.1075,121.0639,91.0533 | O,2OH |  | EPECs | 2-[2-(4'-methoxyphenyl)ethyl]-6,7-epoxy-5,8-dihydroxy-5,6,7,8-tetrahydrochromone(isomer) |
| 17 | 18.34 | C_17_H_16_O_5_ | 253.2 | 301.1060 | 283.0954,255.1001,192.0399,227.1053,91.0530,164.0454 | O,2OH |  | EPECs | 2-(2-phenylethyl)-6,7-epoxy-5,8-dihydroxy-5,6,7,8-tetrahydrochromone(isomer) |
| 18 | 19.03 | C_18_H_16_O_5_ | 227.1,324.6 | 313.1053 | 285.1111,122.0345,121.0632 | O,2OH | OCH_3_ | EPECs | 2-[2-(4'-methoxyphenyl)ethyl]-6,7-epoxy-5,8-dihydroxy-5,6,7,8-tetrahydrochromone(isomer) |
| 19 | 21.17 |  | 242.5,328.2 | 359.1479 | 121.0634 |  |  |  | - |
| 20 | 21.58 | C_17_H_14_O_4_ | 222.4,253.2 | 283.0970 | 255.0970,227.1056,192.0406,91.0533 | 2-O- |  | DEPECs | 2-[2-hydroxy--2-(4'-hydroxyphenyl)ethyl]chromone |
| 21 | 22.13 | C_18_H_16_O_5_ | 226.0,254.4 | 313.1069 | 285.1104,192.0401,121.0635,91.0530 | 2O | OCH_3_ | DEPECs | Oxidoagarochromone B |
| 22 | 22.72 | C_17_H_14_O_4_ | 222.4,254.4 | 283.0970 | 255.0970,227.1056,192.0406,91.0533 | 2O |  | DEPECs | Oxidoagarochromone A |
| 23 | 24.20 | C_17_H_14_O_4_ | 234.2,323.5 | 283.0970 | 255.1000,192.0403,91.0530 | 2O |  | DEPECs | Oxidoagarochromone A(isomer) |
| 24 | 26.99 | C_35_H_30_O_10_ | 233.1,366.1 | 611.1924 | 329.1003,283.0947,419.1483 |  |  | S-Dimers | S1-1 |
| 25 | 27.55 | C_34_H_30_O_9_ | 243.7,318.7 | 582.1889 | 585.168 | FTPECs | FTPECs | S-Dimers | D5-3 |
| 26 | 27.81 | C_19_H_18_O_5_ | 236.6,317.5, | 327.1217 | 191.0697,137.0584， | OCH_3_ | OH,OCH_3_ | FTPECs | 6-methoxy-2-[2-(3'-hydroxy-4'-methoxyphenyl)ethyl]chromone(isomer) |
| 27 | 28.46 | C_18_H_16_O_4_ | 236.6,322.3 | 297.1111 | 206.0579,191.0322,167.0351,91.0529 | OH,OCH_3_ |  | FTPECs | 6-hydroxy-7-methoxy-2-(2-phenylethyl)chromone(isomer) |
| 28 | 30.17 | C_18_H_16_O_4_ | 239.0,328.2 | 297.1107 | 121.0638 | OH | OCH_3_ | FTPECs | 6-hydroxy-2-[2-(4'-methoxyphenyl)ethyl]chromone(isomer) |
| 29 | 31.26 | C_17_H_14_O_3_ | 239.0,324.6, | 267.1009 | 176.0457,137.0221,91.0533 | OH |  | FTPECs | 6-hydroxy-2-(2-phenylethyl)chromone(isomer) |
| 30 | 33.27 | C_20_H_20_O_5_ | 237.8,315.1, | 341.1393 | 220.0176,121.0637 | 2OCH_3_ | OCH_3_ | FTPECs | 6,7-dimethoxy-2-[2-(4'-methoxyphenyl)ethyl]chromone(isomer) |
| 31 | 34.40 | C_19_H_18_O_4_ | 236.6,278.1 | 311.1284 | 220.0720,205.0485,91.0532 | 2OCH_3_ |  | FTPECs | 6,7-dimethoxy-2-(2-phenylethyl)chromone(isomer) |
| 32 | 35.21 | C_35_H_30_O_9_ | 243.7,315.1, | 595.1969 | 565.1846,547.1718,313.1053,283.0935,121.0622,91.0536 | FTPECs | FTPECs | S-Dimers | S8-2 |
| 33 | 36.05 | C_34_H_30_O_8_ | 242.5,318.7 | 567.2003 | 549.1886,531.1179,283.0970,313.1053,121.0622,91.0536 | FTPECs | FTPECs | S-Dimers | S4-1 |
| 34 | 36.40 | C_35_H_32_O_9_ | 242.5,317.5, | 597.2097 | 579.2006,121.0622 | THPECs | FTPECs | D-Dimers | D1-3 |
| 35 | 37.49 | C_34_H_30_O_8_ | 243.7,317.5 | 567.2031 | 549.1906,267.1006,91.0532 | FTPECs | FTPECs | S-Dimers | D1-4 |
| 36 | 38.15 | C_18_H_16_O_3_ | 242.5,331.8 | 281.1160 | 121.0630 |  | OCH_3_ | FTPECs | 2-[2-(4'-methoxyphenyl)ethyl]chromone(isomer) |
| 37 | 39.53 | C_17_H_14_O_2_ | 244.9,296.0 | 251.1056 | 160.0504,173.0582,121.0270,91.0531 |  |  | FTPECs | 2-(2-phenylethyl)chromone |
| 38 | 40.58 | C_17_H_13_ClO_3_ | 252.0,324.6 | 300.0553 | 303.0788 | 3OH,Cl |  | THPECs | 5-chloro-6-hydroxy-2-(2-phenylethyl)chromone |
| 39 | 40.68 | C_19_H_18_O_4_ | 240.1,318.7 | 311.1280 | 190.0609,151.0373,121.0635,91.0524 | OCH_3_ | OCH_3_ | FTPECs | 6-methoxy-2-[2-(4'-methoxyphenyl)ethyl]chromone(isomer) |
| 40 | 42.08 | C_18_H_16_O_3_ | 253.2,321.1 | 281.1174 | 190.0614,151.0377,91.0536 | OCH_3_ |  | FTPECs | 6-methoxy-2-(2-phenylethyl)chromone(isomer) |
| 41 | 43.00 |  | 236.6,331.8 | 255.1324 |  |  |  |  | - |
| 42 | 45.17 | C_19_H_18_O_5_ | 246.1,321.1 | 297.1128 | 121.0622,91.0536 | OH | OCH_3_ | FTPECs | 6-hydroxy-2-[2-(4'-methoxyphenyl)ethyl]chromone(isomer) |
| 43 | 46.20 | C_34_H_28_O_7_ | 249.6,345.7 | 549.1886 | 531.1799,267.1002,91.0530 | FTPECs | FTPECs | S-Dimers | D4-4 |
| 44 | 47.40 | C_35_H_30_O_8_ | 246.1,330.6 | 579.2021 | 488.1468,458.1468,121.0633,91.0529 | FTPECs | EPECs | D-Dimers | S-2 |
| 45 | 48.84 | C_34_H_28_O_7_ | 244.9,334.2 | 549.1913 | 531.1796,458.1357,283.0950 | FTPECs | FTPECs | S-Dimers | S3-2 |

**Supplementary Table S7.** Information of common peaks of 10M

| **NO.** | **t_R_ (min)** | **Formula** | **λ_max_** | **[M+H]^+^**  **measured** | **MS/MS (*m/z*)** | **A ring** | **Bring** | **Type** | **Proposed compound** |
| --- | --- | --- | --- | --- | --- | --- | --- | --- | --- |
| 1 | 4.99 | C_18_H_20_O_8_ | 250.8,324.6, | 365.1222 | 347.1115,329.1010,301.1034,137.0581 | 4OH | OH,OCH_3_ | THPECs | 2-[2-(3-hydroxy-4-methoxyphenyl)ethyl]-5,6,7,8-tetrahydroxy-5,6,7,8-tetrahydrochromone(isomer) |
| 2 | 5.35 | C_18_H_20_O_8_ | 324.6,349.2, | 365.1222 | 347.1100,329.1010//,301.1034,137.0580 | 4OH | OH,OCH_3_ | THPECs | 6.7.8-trihydroxy-5-methoxy-5.6.7.8-tetrahydro-2-[2-(4'-hydroxy-3'-methoxyphenyl)ethyl]chromone |
| 3 | 5.56 | C_18_H_20_O_8_ | 250.8,313.9, | 365.1222 | 347.1100,329.1010,301.1034,137.0580 | 4OH | OH,OCH_3_ | THPECs | 2-[2-(3-hydroxy-4-methoxyphenyl)ethyl]-5,6,7,8-tetrahydroxy-5,6,7,8-tetrahydrochromone(isomer) |
| 4 | 7.67 | C_18_H_18_O_7_ | 250.8,385.3 | 347.1117 | 329.1010,301.1056,122.0346,173.0851 | O,2OH | OH,OCH_3_ | EPECs | 2-[2-(3'-hydroxy-4'-methoxyphenyl)ethyl]-6,7-epoxy-5,8-dihydroxy-5,6,7,8-tetrahydrochromone(isomer) |
| 5 | 7.94 | C_17_H_18_O_6_ | 252.0,330.6, | 319.1187 | 301.1062,283.0950,255.1000,227.1050,91.0570,164.0452 | 4OH |  | THPECs | Agarotetrol |
| 6 | 8.50 | C_17_H_18_O_6_ | 210.6,313.9, | 319.1187 | 301.1080,283.0970,255.1030,227.1049,164.0452,91.0542 | 4OH |  | THPECs | 5.6.7-trihydroxy-5.6.7.8-tetrahydro-2-[2-(3'-hydroxyphenyl)ethyl]chromone |
| 7 | 8.88 | C_17_H_18_O_6_ | 210.6,252.0 | 319.1187 | 301.1062,283.0950,255.0997,227.1050,91.0536,164.0454 | 4OH |  | THPECs | Agarotetrol(isomer) |
| 8 | 9.33 | C_17_H_18_O_6_ | 214.2,252.0 | 319.1161 | 301.1064,283.0951,255.1000,227.1052,91.0536,164.0454 | 4OH |  | THPECs | Agarotetrol(isomer) |
| 9 | 9.99 | C_18_H_18_O_7_ | 256.7,386.5 | 347.1118 | 329.1011,301.1053,137.0580,122.0354 | -O-,2OH | OH,OCH_3_ | EPECs | 2-[2-(3'-hydroxy-4'-methoxyphenyl)ethyl]-6,7-epoxy-5,8-dihydroxy-5,6,7,8-tetrahydrochromone(isomer) |
| 10 | 10.46 | C_18_H_18_O_6_ | 256.7,324.6, | 331.1146 | 313.1053,137.0597,122.0346 | -O-,OH | OH,OCH_3_ | EPECs | 2-[2-(4'-methoxyphenyl)ethyl]-6,7-epoxy-5,8-dihydroxy-5,6,7,8-tetrahydrochromone |
| 11 | 11.51 | C_17_H_14_O_5_ | 210.6,250.8, | 299.0896 | 271.0973,192.0404,107.0481 | 2-O- | OH | DEPECs | 5,6,7,8-diepoxy-2-[2-(4-hydroxy)ethyl]-5,6,7,8-tetrahydrochromone(isomer) |
| 12 | 12.05 | C_18_H_16_O_6_ | 315.1,356.4, | 329.1012 | 301.1397,137.0585 | 2-O- | OH,OCH_3_ | DEPECs | (Oxidoagarochromone C) (isomer) |
| 13 | 12.89 | C_18_H_16_O_6_ | 223.6,253.2, | 329.1016 | 301.1016,192.0407,137.0589 | 2-O- | OH,OCH_3_ | DEPECs | (Oxidoagarochromone C) (isomer) |
| 14 | 13.99 | C_17_H_16_O_5_ | 222.4,252.0, | 301.1081 | 283.0954,255.1001,227.1053,91.0530,164.0454 | -O-,2OH |  | EPECs | 2-(2-phenylethyl)-6,7-epoxy-5,8-dihydroxy-5,6,7,8-tetrahydrochromone(isomer) |
| 15 | 14.64 | C_19_H_20_O_7_ | 226.0,282,9 | 361.1284 | 224.1241,317.1006,137.0587 | -O-,OH,OCH_3_ | OH,OCH_3_ | EPECs | 2-[2-(3'-hydroxy-4'-methoxyphenyl)ethyl]-7,8-epoxy-5-methoxy-6-hydroxy-5,6,7,8-tetrahydrochromone(isomer) |
| 16 | 16.55 | C_18_H_18_O_6_ | 229.5,319.9, | 313.1105 | 285.1091,192.0399,121.0622,91.0529 | -O-,2OH | OCH_3_ | EPECs | 2-[2-(4'-methoxyphenyl)ethyl]-6,7-epoxy-5,8-dihydroxy-5,6,7,8-tetrahydrochromone(isomer) |
| 17 | 16.99 | C_17_H_17_ClO_5_ | 252.0,324.6 | 337.0849 | 319.0725,301.1057,283.0976,91.0529 | 3OH,Cl |  | THPECs | 8-chloro-5,6,7-trihydroxy-5,6,7,8-tetrahydro-2-(2-phenylethyl)chromone(isomer) |
| 18 | 17.91 | C_18_H_18_O_6_ | 223.6,254.4, | 331.1192 | 331.1192,313.1075,121.0639,91.0533 | -O-,2OH |  | EPECs | 2-[2-(4'-methoxyphenyl)ethyl]-6,7-epoxy-5,8-dihydroxy-5,6,7,8-tetrahydrochromone(isomer) |
| 19 | 18.34 | C_17_H_16_O_5_ | 210.6,253.2, | 301.1060 | 283.0954,255.1001,192.0399,227.1053,91.0530,164.0454 | -O-,2OH |  | EPECs | 2-(2-phenylethyl)-6,7-epoxy-5,8-dihydroxy-5,6,7,8-tetrahydrochromone(isomer) |
| 20 | 19.03 | C_18_H_16_O_5_ | 228.3，324.6 | 313.1053 | 285.1111,122.0345,121.0632 | -O-,2OH | OCH_3_ | EPECs | 2-[2-(4'-methoxyphenyl)ethyl]-6,7-epoxy-5,8-dihydroxy-5,6,7,8-tetrahydrochromone(isomer) |
| 21 | 19.98 | C_18_H_16_O_5_ | 231.9,324.6 | 313.1059 | 192.0450,121.0632,91.0529 | -O-,2OH | OCH_3_ | EPECs | 2-[2-(4'-methoxyphenyl)ethyl]-6,7-epoxy-5,8-dihydroxy-5,6,7,8-tetrahydrochromone(isomer) |
| 22 | 21.17 |  | 242.5,331.8 | 359.1479 | 121.0634 |  |  |  | - |
| 23 | 21.58 | C_17_H_14_O_4_ | 222.4,253.2 | 283.0970 | 255.0970,227.1056,192.0406,91.0533 | 2-O- |  | DEPECs | 2-[2-hydroxy--2-(4'-hydroxyphenyl)ethyl]chromone |
| 24 | 22.13 | C_18_H_16_O_5_ | 226.0,253.2 | 313.1069 | 285.1104,192.0401,121.0635,91.0530 | 2-O- | OCH_3_ | DEPECs | Oxidoagarochromone B |
| 25 | 22.72 | C_17_H_14_O_4_ | 222.4,253.2, | 283.0970 | 255.0970,227.1056,192.0406,91.0533 | 2-O- |  | DEPECs | Oxidoagarochromone A |
| 26 | 24.20 | C_17_H_14_O_4_ | 234.2,318.7, | 283.0970 | 255.1000,192.0403,91.0530 | 2-O- |  | DEPECs | (Oxidoagarochromone A) (isomer) |
| 27 | 26.99 | C_36_H_32_O_11_ | 234.2,319.9 | 611.1924 | 329.1003,283.0947,419.1483 |  |  | S-Dimers | S1-1 |
| 28 | 27.55 | C_19_H_18_O_5_ | 242.5,372.1 | 327.1212 | 121.0638,91.0533 | OH,OCH_3_ | OCH_3_ | FTPECs | 6-hydroxy-7-methoxy-2-[2-(4'-methoxyphenyl)ethyl]chromone(isomer) |
| 29 | 27.81 | C_19_H_18_O_5_ | 236.6,324.6 | 327.1217 | 191.0697,137.0584 | OCH3 | OH,OCH_3_ | FTPECs | 6-methoxy-2-[2-(3'-hydroxy-4'-methoxyphenyl)ethyl]chromone(isomer) |
| 30 | 28.46 | C_18_H_16_O_4_ | 236.6,319.9 | 297.1111 | 206.0579,191.0322,167.0351,91.0529 | OH,OCH_3_ |  | FTPECs | 6-hydroxy-7-methoxy-2-(2-phenylethyl)chromone(isomer) |
| 31 | 29.27 | C_18_H_16_O_6_ | 247.3,380.5 | 329.1042 | 137.0583 | 2OH | OH,OCH_3_ | FTPECS | 6,7-dihydroxy-2-[2-(4'-hydroxy-3'-methoxyphenyl)ethyl]chromone(isomer) |
| 32 | 30.17 | C_18_H_16_O_4_ | 239.0,324.6 | 297.1107 | 121.0638 | OH | OCH_3_ | FTPECs | 6-hydroxy-2-[2-(4'-methoxyphenyl)ethyl]chromone(isomer) |
| 33 | 31.26 | C_17_H_14_O_3_ | 239.0,325.8 | 267.1009 | 176.0457,137.0221,91.0533 | OH |  | FTPECs | 6-hydroxy-2-(2-phenylethyl)chromone(isomer) |
| 34 | 33.27 | C_20_H_20_O_5_ | 236.6,316.3 | 341.1393 | 220.0176,121.0637 | 2OCH_3_ | OCH_3_ | FTPECs | 6,7-dimethoxy-2-[2-(4'-methoxyphenyl)ethyl]chromone(isomer) |
| 35 | 34.40 | C_19_H_18_O_4_ | 236.6,279.3 | 311.1284 | 220.0720,205.0485,91.0532 | 2OCH_3_ |  | FTPECs | 6,7-dimethoxy-2-(2-phenylethyl)chromone(isomer) |
| 36 | 36.05 | C_34_H_30_O_8_ | 242.5,318.7 | 567.2003 | 549.1886,531.1179,283.0970,313.1053,121.0622,91.0536 | FTPECs | FTPECs | S-Dimers | S4-1 |
| 37 | 37.49 | C_34_H_30_O_8_ | 243.7,318.7 | 567.2031 | 549.1906,267.1006,91.0532 | FTPECs | FTPECs | S-Dimers | D1-4 |
| 38 | 39.53 | C_17_H_14_O_2_ | 247.3,296.0 | 251.1056 | 160.0504,173.0582,121.0270,91.0531 |  |  | FTPECs | 2-(2-phenylethyl)chromone |
| 39 | 40.68 | C_19_H_18_O_4_ | 241.3,319.9, | 311.1280 | 190.0609,151.0373,121.0635,91.0524 | OCH_3_ | OCH_3_ | FTPECs | 6-methoxy-2-[2-(4'-methoxyphenyl)ethyl]chromone(isomer) |
| 40 | 42.08 | C_18_H_16_O_3_ | 252.0,325.8, | 281.1174 | 190.0614,151.0377,91.0536 | OCH_3_ |  | FTPECs | 6-methoxy-2-(2-phenylethyl)chromone(isomer) |
| 41 | 43.00 | C_18_H_20_O_8_ | 234.2,324.6 | 255.1324 | 347.1115,329.1010,301.1034,137.0581 |  |  | 未鉴定 |  |

**Supplementary Table S8.** Information of common peaks of 12M

| **NO.** | **t_R_ (min)** | **Formula** | **λ_max_** | **[M+H]^+^**  **measured** | **MS/MS (*m/z*)** | **A ring** | **Bring** | **Type** | **Proposed compound** |
| --- | --- | --- | --- | --- | --- | --- | --- | --- | --- |
| 1 | 4.99 | C_18_H_20_O_8_ | 250.8,343.4 | 365.1222 | 347.1115,329.1010,301.1034,137.0581 | 4OH | OH,OCH_3_ | THPECs | 2-[2-(3-hydroxy-4-methoxyphenyl)ethyl]-5,6,7,8-tetrahydroxy-5,6,7,8-tetrahydrochromone(isomer) |
| 2 | 5.56 | C_18_H_20_O_8_ | 252.0,318.7 | 365.1222 | 347.1100,329.1010,301.1034,137.0580 | 4OH | OH,OCH_3_ | THPECs | 2-[2-(3-hydroxy-4-methoxyphenyl)ethyl]-5,6,7,8-tetrahydroxy-5,6,7,8-tetrahydrochromone(isomer) |
| 3 | 7.67 | C_18_H_18_O_7_ | 252.0,343.4 | 347.1117 | 329.1010,301.1056,122.0346,173.0851 | -O-,2OH | OH,OCH_3_ | EPECs | 2-[2-(3'-hydroxy-4'-methoxyphenyl)ethyl]-6,7-epoxy-5,8-dihydroxy-5,6,7,8-tetrahydrochromone(isomer) |
| 4 | 7.94 | C_17_H_18_O_6_ | 210.6,252.0 | 319.1187 | 301.1062,283.0950,255.1000,227.1050,91.0570,164.0452 | 4OH |  | THPECs | Agarotetrol |
| 5 | 8.50 | C_17_H_18_O_6_ | 341.1,368.5 | 319.1187 | 301.1080,283.0970,255.1030,227.1049,164.0452,91.0542 | 4OH |  | THPECs | 5.6.7-trihydroxy-5.6.7.8-tetrahydro-2-[2-(3'-hydroxyphenyl)ethyl]chromone |
| 6 | 8.88 | C_17_H_18_O_6_ | 253.2,330.6 | 319.1187 | 301.1062,283.0950,255.0997,227.1050,91.0536,164.0454 | 4OH |  | THPECs | Agarotetrol(isomer) |
| 7 | 9.33 | C_17_H_18_O_6_ | 210.6,253.2 | 319.1161 | 301.1064,283.0951,255.1000,227.1052,91.0536,164.0454 | 4OH |  | THPECs | Agarotetrol(isomer) |
| 8 | 9.99 | C_18_H_18_O_7_ | 253.20 | 347.1118 | 329.1011,301.1053,137.0580,122.0354 | -O-,2OH | OH,OCH_3_ | EPECs | 2-[2-(3'-hydroxy-4'-methoxyphenyl)ethyl]-6,7-epoxy-5,8-dihydroxy-5,6,7,8-tetrahydrochromone(isomer) |
| 9 | 10.46 | C_18_H_18_O_6_ | 210.6,340.0 | 331.1146 | 313.1053,137.0597,122.0346 | -O-,OH | OH,OCH_3_ | EPECs | 2-[2-(4'-methoxyphenyl)ethyl]-6,7-epoxy-5,8-dihydroxy-5,6,7,8-tetrahydrochromone |
| 10 | 11.51 | C_17_H_14_O_5_ | 210.6,250.8, | 299.0896 | 271.0973,192.0404,107.0481 | 2-O- | OH | DEPECs | 5,6,7,8-diepoxy-2-[2-(4-hydroxy)ethyl]-5,6,7,8-tetrahydrochromone(isomer) |
| 11 | 12.05 | C_18_H_16_O_6_ | 254.4,324.6 | 329.1012 | 301.1397,137.0585 | 2-O- | OH,OCH_3_ | DEPECs | (Oxidoagarochromone C) (isomer) |
| 12 | 12.89 | C_18_H_16_O_6_ | 222.4,324.6 | 329.1016 | 301.1016,192.0407,137.0589 | 2-O- | OH,OCH_3_ | DEPECs | Oxidoagarochromone C (isomer) |
| 13 | 13.99 | C_17_H_16_O_5_ | 221.2,252.0 | 301.1081 | 283.0954,255.1001,227.1053,91.0530,164.0454 | -O-,2OH |  | EPECs | 2-[2-(4'-hydroxy)ethyl]-6,7-epoxy-5,8-dihydroxy-5,6,7,8-tetrahydrochromone(isomer) |
| 14 | 14.64 | C_19_H_20_O_7_ | 281.7,322.3 | 361.1284 | 224.1241,317.1006,137.0587 | -O-,OH,OCH_3_ | OH,OCH_3_ | EPECs | 2-[2-(3'-hydroxy-4'-methoxyphenyl)ethyl]-7,8-epoxy-5-methoxy-6-hydroxy-5,6,7,8-tetrahydrochromone(isomer) |
| 15 | 16.55 | C_18_H_18_O_6_ | 227.1,321.1 | 313.1105 | 285.1091,192.0399,121.0622,91.0529 | -O-,2OH | OCH_3_ | EPECs | 2-[2-(4'-methoxyphenyl)ethyl]-6,7-epoxy-5,8-dihydroxy-5,6,7,8-tetrahydrochromone(isomer) |
| 16 | 16.99 | C_17_H_17_ClO_5_ | 215.3,250.8 | 337.0849 | 319.0725,301.1057,283.0976,91.0529 | 3OH,Cl |  | THPECs | 8-chloro-5,6,7-trihydroxy-5,6,7,8-tetrahydro-2-(2-phenylethy)chromone(isomer) |
| 17 | 17.91 | C_18_H_18_O_6_ | 224.8,254.4 | 331.1192 | 331.1192,313.1075,121.0639,91.0533 | -O-,2OH | OCH_3_ | EPECs | 2-[2-(4'-methoxyphenyl)ethyl]-6,7-epoxy-5,8-dihydroxy-5,6,7,8-tetrahydrochromone(isomer) |
| 18 | 18.34 | C_17_H_16_O_5_ | 210.6,253.2 | 301.1060 | 283.0954,255.1001,192.0399,227.1053,91.0530,164.0454 | -O-,2OH |  | EPECs | 2-(2-phenylethyl)-6,7-epoxy-5,8-dihydroxy-5,6,7,8-tetrahydrochromone(isomer) |
| 19 | 19.03 | C_18_H_16_O_5_ | 227.1,327.0 | 313.1053 | 285.1111,122.0345,121.0632 | -O-,2OH | OCH_3_ | EPECs | 2-[2-(4'-methoxyphenyl)ethyl]-6,7-epoxy-5,8-dihydroxy-5,6,7,8-tetrahydrochromone(isomer) |
| 20 | 21.17 |  | 242.5,330.6 | 359.1479 | 121.0634 |  |  |  | - |
| 21 | 21.58 | C_17_H_14_O_4_ | 222.4,253.2 | 283.0970 | 255.0970,227.1056,192.0406,91.0533 | 2-O- |  | DEPECs | 2-[2-hydroxy--2-(4'-hydroxyphenyl)ethyl]chromone |
| 22 | 22.13 | C_18_H_16_O_5_ | 223.6,254.4 | 313.1069 | 285.1104,192.0401,121.0635,91.0530 | 2-O- | OCH_3_ | DEPECs | Oxidoagarochromone B |
| 23 | 22.72 | C_17_H_14_O_4_ | 218.9,254.4 | 283.0970 | 255.0970,227.1056,192.0406,91.0533 | 2-O- |  | DEPECs | Oxidoagarochromone A |
| 24 | 23.36 | C_18_H_16_O_5_ | 230.7,280.5 | 313.1076 | 285.1092，121.0633,192.0633,91.0528 | 2-O- | OCH_3_ | DEPECs | Oxidoagarochromone B(isomer) |
| 25 | 24.20 | C_17_H_14_O_4_ | 233.1,281.7 | 283.0970 | 255.1000,192.0403,91.0530 |  |  | DEPECs | Oxidoagarochromone A(isomer) |
| 26 | 26.99 | C_36_H_32_O_11_ | 235.4,317.5 | 611.1924 | 329.1003,283.0947,419.1483 | FTPECs | THPECs | D-Dimers | S1-1 |
| 27 | 27.55 | C_19_H_18_O_5_ | 241.3,323.5 | 327.1212 | 121.0638,91.0533 | OH,OCH_3_ | OCH_3_ | FTPECs | 6-hydroxy-7-methoxy-2-[2-(4'-methoxyphenyl)ethyl]chromoe(isomer) |
| 28 | 28.46 | C_18_H_16_O_4_ | 233.1,323.5 | 297.1111 | 206.0579,191.0322,167.0351,91.0529 | OH,OCH_3_ |  | FTPECs | 6-hydroxy-7-methoxy-2-(2-phenylethyl)chromone(isomer) |
| 29 | 29.27 | C_18_H_16_O_6_ | 247.3,380.5 | 329.1042 | 137.0583 | 2OH | OH,OCH_3_ | FTPECS | 6,7-dihydroxy-2-[2-(4'-hydroxy-3'-methoxyphenyl)ethyl]chromone(isomer) |
| 30 | 30.17 | C_18_H_16_O_4_ | 236.6,322.3 | 297.1107 | 121.0638 | OH | OCH_3_ | FTPECs | 6-hydroxy-2-[2-(4'-methoxyphenyl)ethyl]chromone(isomer) |
| 31 | 31.26 | C_17_H_14_O_3_ | 236.6,324.6 | 267.1009 | 176.0457,137.0221,91.0533 | OH |  | FTPECs | 6-hydroxy-2-(2-phenylethyl)chromone(isomer) |
| 32 | 33.27 | C_20_H_20_O_5_ | 248.4,318.7 | 341.1393 | 220.0176,121.0637 | 2OCH_3_ | OCH_3_ | FTPECSs | 6,7-dimethoxy-2-[2-(4'-methoxyphenyl)ethyl]chromone(isomer) |
| 33 | 34.40 | C_19_H_18_O_4_ | 239.0,323.5 | 311.1284 | 220.0720,205.0485,91.0532 | 2OCH_3_ |  | FTPECs | 6,7-dimethoxy-2-(2-phenylethyl)chromone(isomer) |
| 34 | 35.21 | C_35_H_30_O_9_ | 239.0,324.6 | 595.1969 | 565.1846,547.1718,313.1053,283.0935,121.0622,91.0536 | FTPECs | FTPECs | S-Dimers | S8-2 |
| 35 | 36.05 | C_34_H_30_O_8_ | 236.6,315.1 | 567.2003 | 549.1886,531.1179,283.0970,313.1053,121.0622,91.0536 | FTPECs | FTPECs | S-Dimers | S4-1 |
| 36 | 36.40 | C_35_H_32_O_9_ | 235.4,279.3 | 597.2097 | 579.2006,121.0622 | THPECs | FTPECs | D-Dimers | D1-3 |
| 37 | 39.53 | C_17_H_14_O_2_ | 243.7,323.5 | 251.1056 | 160.0504,173.0582,121.0270,91.0531 |  |  | FTPECs | 2-(2-phenylethyl)chromone |
| 38 | 43.00 |  | 241.3,322.3 | 255.1324 |  |  |  |  | - |
| 39 | 45.17 | C_18_H_16_O_4_ | 241.3,321.1 | 297.1128 | 121.0622,91.0536 | OH | OCH_3_ | FTPECs | 6-hydroxy-2-[2-(4'-methoxyphenyl)ethyl]chromone(isome) |
| 40 | 46.20 | C_34_H_28_O_7_ | 252.0,322.3 | 549.1886 | 531.1799,267.1002,91.0530 | FTPECs | FTPECs | S-Dimers | D4-4 |
| 41 | 47.40 | C_35_H_30_O_8_ | 234.2,343.4 | 579.2021 | 488.1468,458.1468,121.0633,91.0529 | FTPECs | EPECs | D-Dimers | S-2 |
| 42 | 48.84 | C_34_H_2_8O_7_ | 249.6,343.4 | 549.1913 | 531.1796,458.1357,283.0950 | FTPECs | FTPECs | S-Dimers | S3-2 |
| 43 | 62.08 | C_20_H_20_O_7_ | 252.0,318.7 | 373.1287 | 220.0176,121.0637 | 2OCH_3_ | OCH_3_ | FTPECs | 5-hydroxy-6,7-dimethoxy-2-[2-(3'-hydroxy-4-methoxyphenyl)ethyl]chromone |

# Supplemented with information on differences and differential metabolites between each of the two adjacent sample groups.

**Supplementary Table S9.** Identification information of differential compounds between BM and 2M.

| **t_R_(min)** | **[M+H]^+^ (*m/z*)** | **FC（2m/bm）** | **VIP** | **p** | **Trend** | **A Ring** | **Bring** | **Type** | **Proposed Compound** |
| --- | --- | --- | --- | --- | --- | --- | --- | --- | --- |
| 7.94 | 319.1168 | 462.38 | 11.3787 | 8.77E-05 | 2m | 4OH |  | THPECs | Agarotetrol |
| 8.88 | 319.1166 | 260.844 | 7.12804 | 3.17E-06 | 2m | 4OH |  | THPECs | Agarotetrol(isomer) |
| 12.88 | 329.1009 | 18.724 | 6.14927 | 0.10498 | 2m | 2-O- | OH,OCH_3_ | DEPECs | Oxidoagarochromone C(isomer) |
| 18.37 | 301.1065 | 52.178 | 9.59 | 7.57E-07 | 2m | -O-,2OH |  | EPECs | 2-(2-phenylethyl)-6,7-epoxy-5,8-dihydroxy-5,6,7,8-tetrahydrochromone |
| 22.22 | 313.1065 | 449.16 | 18.5064 | 0.0078419 | 2m | 2-O- | OCH_3_ | DEPECs | Oxidoagarochromone B |
| 22.80 | 283.097 | 1281.6 | 35.7797 | 1.77E-05 | 2m | 2-O- |  | DEPECs | Oxidoagarochromone A |
| 24.26 | 283.0954 | 64.743 | 7.32083 | 1.39E-07 | 2m | 2-O- |  | DEPECs | Oxidoagarochromone A(isomer) |
| 28.46 | 297.1111 | 19.178 | 7.22823 | 0.0007729 | 2m | OH,OCH_3_ |  | FTPECs | 6-hydroxy-7-methoxy-2-(2-phenylethyl)chromone(isomer) |
| 31.31 | 267.1006 | 29.919 | 13.6068 | 0.0014399 | 2m | OH |  | FTPECs | 6-hydroxy-2-(2-phenylethyl)chromone(isomer) |
| 33.35 | 341.1375 | 259.33 | 11.0239 | 1.97E-08 | 2m | 2OCH_3_ | OCH_3_ | FTPECs | 6,7-dimethoxy-2-[2-(4'-methoxyphenyl)ethyl]chromone(isomer) |
| 34.43 | 311.1276 | 366.72 | 28.1441 | 2.69E-06 | 2m | 2OCH_3_ |  | FTPECs | 6,7-dimethoxy-2-(2-phenylethyl)chromone(isomer) |
| 37.28 | 565.1863 | 6.28 | 6.68431 | 2.09E-05 | 2m | THPECs | FTPECs | S-Dimers | S7-1 |
| 39.55 | 251.1058 | 14.127 | 14.4877 | 0.011579 | 2m |  |  | FTPECs | 2-(2-phenylethenyl)chromone |
| 40.73 | 311.1269 | 23.016 | 8.81883 | 0.0022582 | 2m | OCH_3_ | OCH_3_ | FTPECs | 6-methoxy-2-[2-(4'-methoxyphenyl)ethyl]chromone(isomer) |
| 41.91 | 281.1163 | 92.478 | 16.826 | 0.0065385 | 2m | OCH_3_ |  | FTPECs | 6-methoxy-2-(2-phenylethyl)chromone(isomer) |
| 42.23 | 255.1345 | 9.207 | 6.16243 | 0.00034376 | 2m |  |  | - |  |
| 47.48 | 579.2021 | 11.69 | 6.4475 | 8.72E-05 | 2m | THPECs | FTPECs | S-Dimers | S-2 |
| 48.95 | 549.1913 | 38.604 | 11.937 | 0.00060829 | 2m | THPECs | FTPECs | S-Dimers | S3-2 |

**
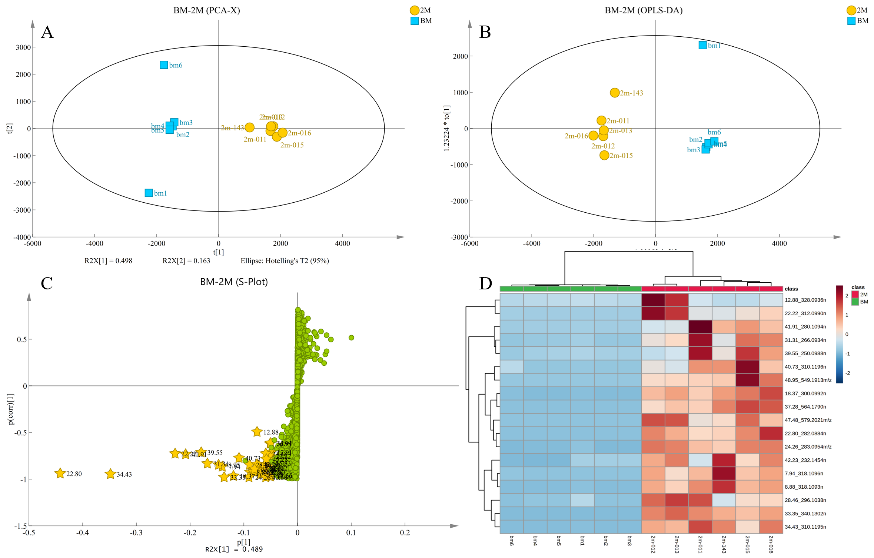
**

**Supplementary Fig. S3** Differential compounds between BM and 2M. (A) Representation of PCA and OPLS-DA analysis. (B) S-plot between BM and 2M. (C) Heatmap of differential 2-(2-phenylethyl)chromone compounds from BM *vs.* 2M comparisons.


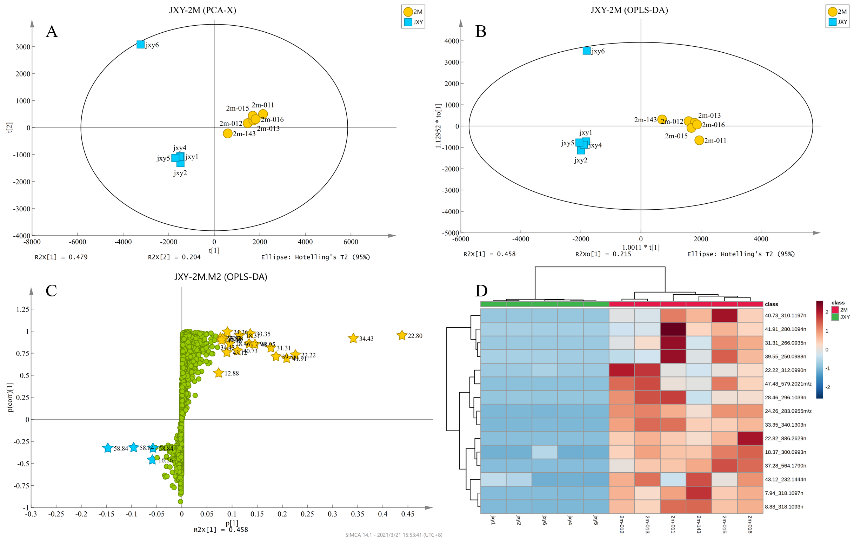


**Supplementary Fig. S4** Differential compounds between JYM and 2M. (A) Representation of PCA and OPLS-DA analysis. (B) S-plot between JYM and 2M. (C) Heatmap of differential 2-(2-phenylethyl)chromone compounds from JYM *vs.* 2M comparisons.


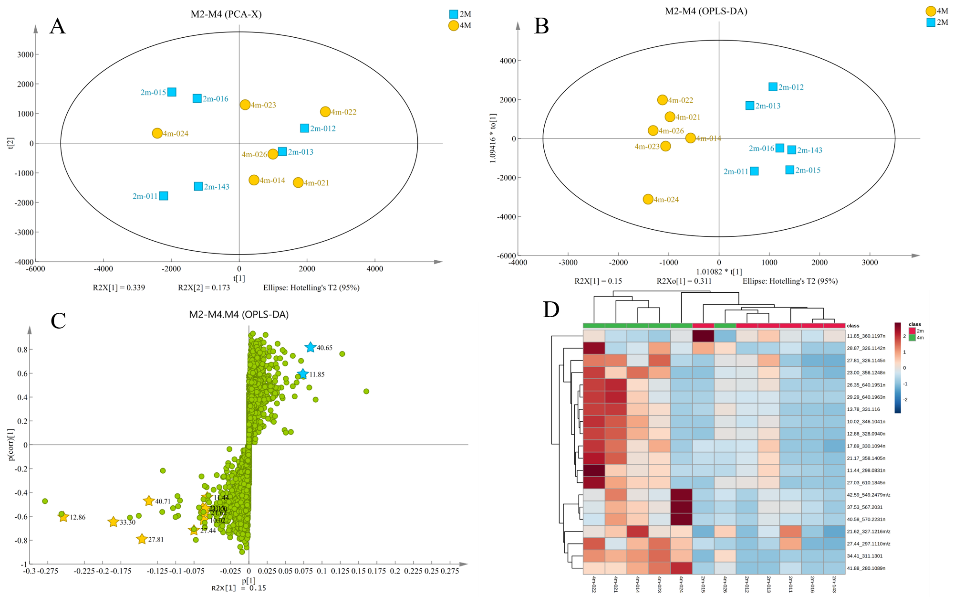


**Supplementary Fig. S5** Differential compounds between 2M and 4M. (A) Representation of PCA and OPLS-DA analysis. (B) S-plot between 2M and 4M. (C) Heatmap of differential 2-(2-phenylethyl)chromone compounds from 2M *vs.* 4M comparisons.


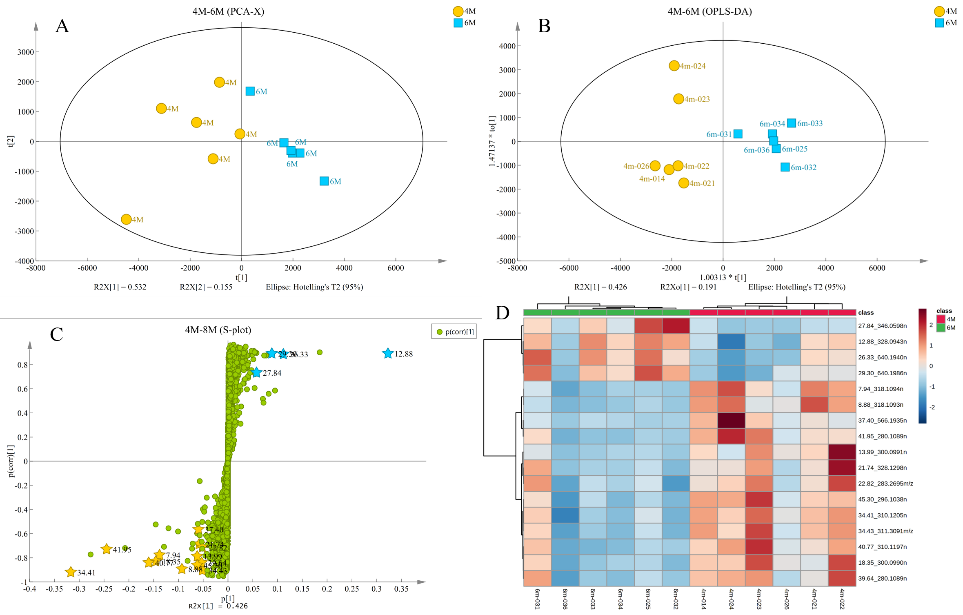


**Supplementary Fig. S6** Differential compounds between 4M and 6M. (A) Representation of PCA and OPLS-DA analysis. (B) S-plot between 4M and 6M. (C) Heatmap of differential 2-(2-phenylethyl)chromone compounds from 4M *vs.* 6M comparisons.


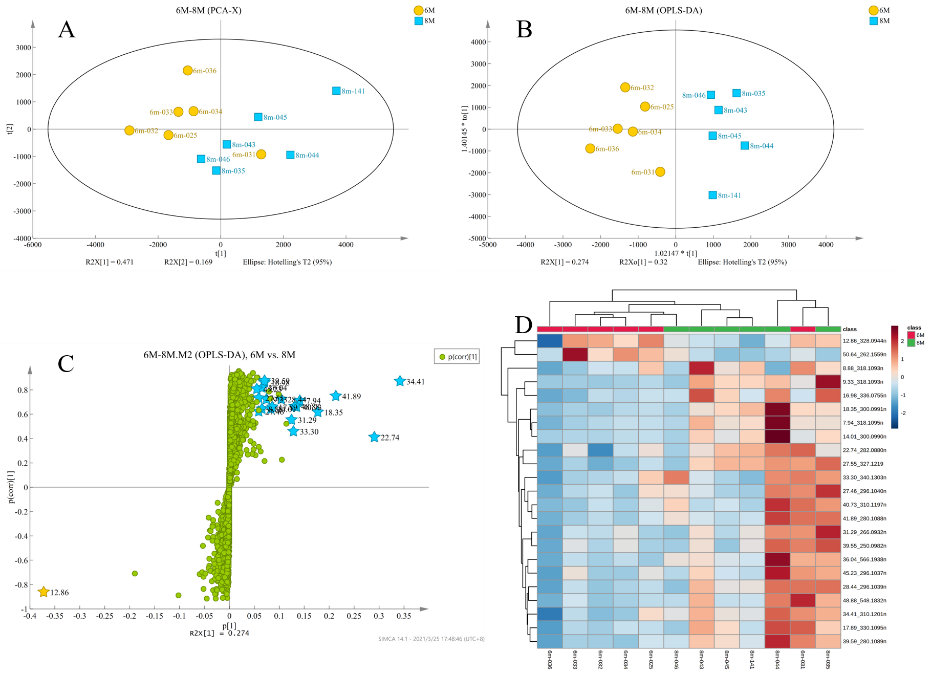


**Supplementary Fig. S7** Differential compounds between 6M and 8M. (A) Representation of PCA and OPLS-DA analysis. (B) S-plot between 6M and 8M. (C) Heatmap of differential 2-(2-phenylethyl)chromone compounds from 6M *vs.* 8M comparisons.


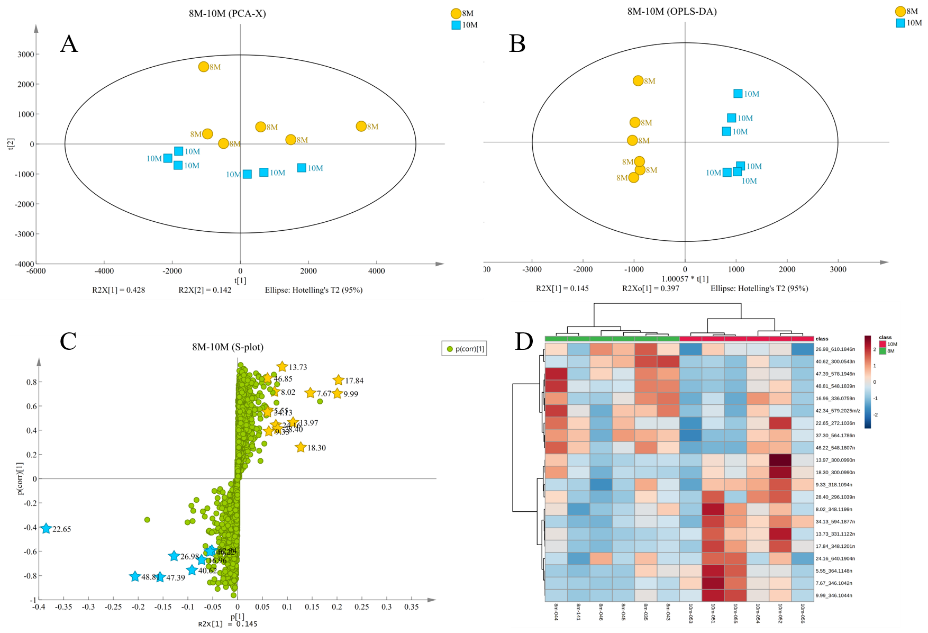


**Supplementary Fig. S8** Differential compounds between 8M and 10M. (A) Representation of PCA and OPLS-DA analysis. (B) S-plot between 8M and 10M. (C) Heatmap of differential 2-(2-phenylethyl)chromone compounds from 8M *vs.* 10M comparisons.


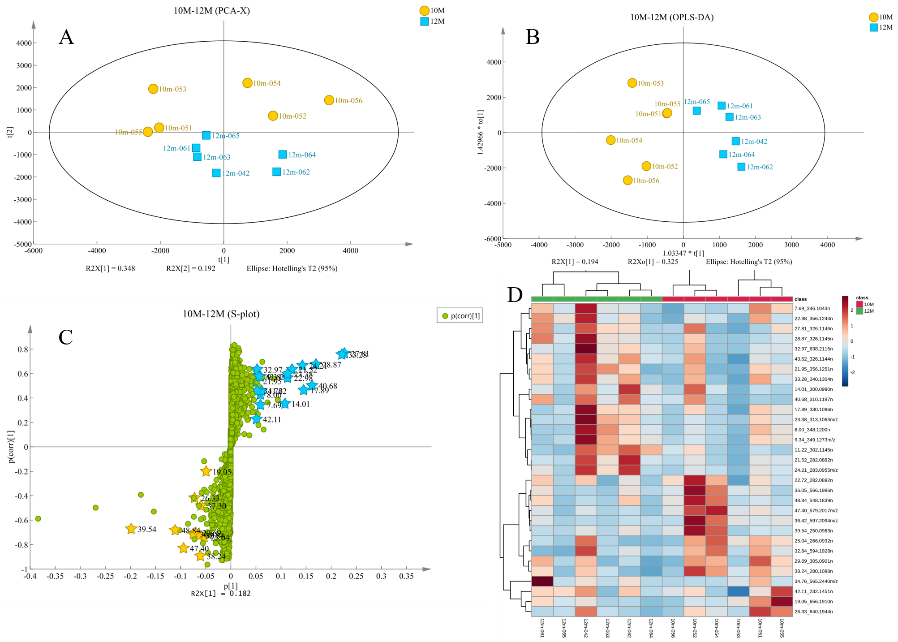


**Supplementary Fig. S9** Differential compounds between 10M and 12M. (A) Representation of PCA and OPLS-DA analysis. (B) S-plot between 10M and 12M. (C) Heatmap of differential 2-(2-phenylethyl)chromone compounds from 10M *vs.* 12M comparisons.

**Supplementary Table S10.** Identification information of differential compounds between JXY and 2M.

| **t_R_(min)** | **[M+H]^+^ (*m/z*)** | **FC** | **VIP** | **p** | **Trend** | **A Ring** | **Bring** | **Type** | **Proposed Compound** |
| --- | --- | --- | --- | --- | --- | --- | --- | --- | --- |
| 7.94 | 319.1168 | 602.33 | 10.2796 | 4.41E-05 | 2m | 4OH |  | THPECs | Agarotetrol |
| 8.88 | 319.1166 | 360.683 | 6.56849 | 5.50E-06 | 2m | 4OH |  | THPECs | Agarotetrol(isomer) |
| 12.88 | 329.1009 | 37.074 | 5.64814 | 0.11754 | 2m | 2-O- | OH,OCH_3_ | DEPECs | Oxidoagarochromone C(isomer) |
| 18.37 | 301.1065 | 14.051 | 8.96017 | 1.46E-05 | 2m | -O-,2OH |  | EPECs | 2-(2-phenylethyl)-6,7-epoxy-5,8-dihydroxy-5,6,7,8-tetrahydrochromone(isomer) |
| 22.22 | 313.1065 | 446.796 | 17.4947 | 0.015491 | 2m | 2-O- | OCH_3_ | DEPECs | Oxidoagarochromone B |
| 22.80 | 283.097 | 1328.4 | 5.93706 | 0.00039315 | 2m | 2-O- |  | DEPECs | Oxidoagarochromone A |
| 24.26 | 283.0954 | 7.0174 | 7.03559 | 1.09E-06 | 2m | 2-O- |  | DEPECs | Oxidoagarochromone A(isomer) |
| 28.46 | 297.1111 | 198.05 | 7.33482 | 0.0029184 | 2m | OH,OCH_3_ |  | FTPECs | 6-hydroxy-7-methoxy-2-(2-phenylethyl)chromone(isomer) |
| 31.31 | 267.1006 | 468.54 | 13.7368 | 0.0076683 | 2m | OH |  | FTPECs | 6-methoxy-2-(2-phenylethyl)chromone(isomer) |
| 33.35 | 341.1375 | 396.16 | 10.4901 | 3.87E-06 | 2m | 2OCH_3_ | OCH_3_ | FTPECs | 6,7-dimethoxy-2-[2-(4'-methoxyphenyl)ethyl]chromone(isomer) |
| 34.43 | 311.1276 | 1466.72 | 28.1441 | 2.69E-06 | 2m | 2OCH_3_ |  | FTPECs | 6,7-dimethoxy-2-(2-phenylethyl)chromone(isomer) |
| 37.28 | 565.1863 | 8.2774 | 6.23834 | 9.69E-05 | 2m | THPECs | FTPECs | S-Dimers | S7-1 |
| 39.55 | 251.1058 | 524.134 | 14.5223 | 0.028054 | 2m |  |  | FTPECs | 2-(2-phenylethenyl)chromone |
| 40.73 | 311.1269 | 435.18 | 8.52994 | 0.004003 | 2m | OCH_3_ | OCH_3_ | FTPECs | 6-methoxy-2-[2-(4'-methoxyphenyl)ethyl]chromone(isomer) |
| 41.91 | 281.1163 | 633.56 | 16.1707 | 0.026764 | 2m | OCH_3_ |  | FTPECs | 6-methoxy-2-(2-phenylethyl)chromone(isomer) |
| 43.12 | 255.1345 | 6.5971 | 6.86154 | 0.0018076 | 2m |  |  | - |  |
| 47.48 | 579.2021 | 8.9737 | 6.11931 | 0.00068699 | 2m | FTPECs | FTPECs | S-Dimers | S-2 |

**Supplementary Table S11.** Identification information of differential compounds between 2M and 4M.

| **t_R_(min)** | **[M+H]^+^ (*m/z*)** | **FC** | **VIP** | ***p*** | **Trend** | **A ring** | **B ring** | **Type** | **Proposed compound** |
| --- | --- | --- | --- | --- | --- | --- | --- | --- | --- |
| 10.02 | 347.1114 | 5.6312 | 5.4636 | 0.024154 | 4m | -O-, 2OH | OH,OCH_3_ | EPECs | 2-[2-(3'-hydroxy-4'-methoxyphenyl)ethyl]-6,7-epoxy-5,8-dihydroxy-5,6,7,8-tetrahydrochromone(isomer) |
| 11.44 | 299.0899 | 2.8117 | 5.55047 | 0.12908 | 4m | 2-O- | OH | DEPECs | 5,6:7,8-diepoxy-2-[2-(4-hydroxy)ethyl]-5,6,7,8-tetrahydrochromone(isomer) |
| 11.85 | 361.1261 | 0.58206 | 5.79329 | 0.14132 | 2m | -O-, OH,OCH_3_ | OH,OCH_3_ | EPECs | 2-[2-(3'-hydroxy-4'-methoxyphenyl)ethyl]-7,8-epoxy-5-methoxy-6-hydroxy-5,6,7,8-tetrahydrochromone(isomer) |
| 12.86 | 329.1013 | 7.4553 | 14.4506 | 0.023233 | 4m | 2-O- | OH,OCH_3_ | DEPECs | Oxidoagarochromone C(isomer) |
| 13.78 | 331.1164 | 4.2757 | 4.30821 | 0.018128 | 4m | -O-,2OH | OCH_3_ | EPECs | 2-[2-(4'-methoxyphenyl)ethyl]-6,7-epoxy-5,8-dihydroxy-5,6,7,8-tetrahydrochromone |
| 17.89 | 331.1167 | 2.4454 | 8.90939 | 0.056241 | 4m | -O-,2OH | OCH_3_ | EPECs | 2-[2-(4'-methoxyphenyl)ethyl]-6,7-epoxy-5,8-dihydroxy-5,6,7,8-tetrahydrochromone |
| 21.17 | 313.1076 | 3.0214 | 5.97864 | 0.077348 | 4m | 2-O- | OCH_3_ | DTPECs | 5,6:7,8-diepoxy-2-[2-(4-methoxy)ethyl]-5,6,7,8-tetrahydrochromone(isomer) |
| 21.62 | 327.1216 | 2.3801 | 4.67679 | 0.044185 | 4m | 2OCH_3_ | OH | FTPECs | 6,7-dimethoxy-2-[2-(3'-hydroxyphenyl)ethyl]chromone |
| 23.00 | 357.1321 | 3.0234 | 5.50378 | 0.041751 | 4m | 2OCH_3_ | OH,OCH_3_ | FTPECs | 6,7-dimethoxy-2-[2-(3'-hydroxy-4'-methoxyphenyl)ethyl]chromone(isomer) |
| 26.35 | 641.2023 | 4.5926 | 9.35067 | 0.060529 | 4m | THPECs | FTPECs | S-Dimers | S1-2(isomer) |
| 27.03 | 611.1918 | 3.7519 | 9.80583 | 0.047741 | 4m | THPECs | FTPECs | S-Dimers | S1-1(isomer) |
| 27.44 | 297.111 | 2.4637 | 5.8559 | 0.040804 | 4m | OCH_3_ | OH | FTPECs | 6-methoxy-2-[2-(4'-hydroxyphenyl)ethyl]chromone(isomer) |
| 27.81 | 327.1218 | 3.4328 | 11.4833 | 0.049628 | 4m | OCH_3_ | OH,OCH_3_ | FTPECs | 6-methoxy-2-[2-(3'-hydroxy-4'-methoxyphenyl)ethyl]chromone(isomer) |
| 28.87 | 327.1218 | 2.9907 | 5.91546 | 0.13586 | 4m | 2OCH_3_ | OCH_3_ | FTPECs | 6,7-dimethoxy-2-[2-(4'-methoxyphenyl)ethyl]chromone(isomer) |
| 29.29 | 641.2017 | 4.779 | 5.64027 | 0.04778 | 4m | THPECs | FTPECs | S-Dimers | S1-2(isomer) |
| 34.41 | 311.1301 | 5.8351 | 5.29381 | 1.15E-06 | 4m | 2OCH_3_ |  | FTPECs | 6,7-dimethoxy-2-(2-phenylethyl)chromone(isomer) |
| 37.53 | 567.2031 | 3.9621 | 5.94726 | 0.05713 | 4m | THPECs | FTPECs | S-Dimers | S4-1 |
| 40.58 | 311.1261 | 5.3263 | 4.88414 | 0.059076 | 4m | OCH_3_ | OCH_3_ | FTPECs | 6-methoxy-2-[2-(4'-methoxyphenyl )ethyl]chromone(isomer) |
| 41.88 | 281.1165 | 6.8552 | 20.0592 | 0.00092905 | 4m | OCH_3_ |  | FTPECs | 6-methoxy-2-(2-phenylethyl)chromone(isomer) |
| 42.59 | 549.2479 | 6.3313 | 8.28605 | 0.050653 | 4m | THPECs | FTPECs | S-Dimers | S3-2 |

**Supplementary Table S12.** Identification information of differential compounds between 4M and 6M.

| **t_R_(min)** | **[M+H]^+^ (*m/z*)** | **FC** | **VIP** | ***p*** | **Trend** | **A ring** | **B ring** | **Type** | **Proposed compound** |
| --- | --- | --- | --- | --- | --- | --- | --- | --- | --- |
| 7.94 | 319.1167 | 0.32948 | 11.0494 | 0.00067155 | 4m | 4OH |  | THPECs | Agarotetrol |
| 8.88 | 319.1166 | 0.2249 | 7.18256 | 0.00049527 | 4m | 4OH |  | THPECs | Agarotetrol(isomer) |
| 12.88 | 329.1016 | 1.8406 | 25.4508 | 0.02968 | 6m | 2-O- | OH,OCH_3_ | DEPECs | Oxidoagarochromone C(isomer) |
| 13.99 | 301.1064 | 0.28494 | 4.72903 | 0.011914 | 4m | -O-,2OH |  | EPECs | 2-(2-phenylethyl)-6,7-epoxy-5,8-dihydroxy-5,6,7,8-tetrahydrochromone(isomer) |
| 18.35 | 301.1062 | 0.39882 | 11.4107 | 0.0073277 | 4m | -O-,2OH |  | EPECs | 2-(2-phenylethyl)-6,7-epoxy-5,8-dihydroxy-5,6,7,8-tetrahydrochromone(isomer) |
| 21.74 | 329.1371 | 0.37039 | 4.5148 | 0.080215 | 4m | 2-O- | OH,OCH_3_ | DEPECs | Oxidoagarochromone C(isomer) |
| 22.65 | 283.0796 | 0.57459 | 4.15549 | 0.036486 | 4m | 2-O- |  | DEPECs | Oxidoagarochromone A |
| 26.33 | 641.2027 | 2.5029 | 8.79275 | 0.026382 | 6m | THPECs | FTPECs | S-Dimers | S1-2(isomer) |
| 27.84 | 327.1212 | 5.7836 | 4.51612 | 0.017675 | 6m | OCH_3_ | OH,OCH_3_ | FTPECs | 6-methoxy-2-[2-(3'-hydroxy-4'-methoxyphenyl)ethyl]chromone(isomer) |
| 29.3 | 641.2024 | 2.4985 | 6.88161 | 0.022078 | 6m | THPECs | FTPECs | Dimers | S1-2(isomer) |
| 34.41 | 311.1278 | 0.47989 | 24.2259 | 0.0033213 | 4m | 2OCH_3_ |  |  | 6,7-dimethoxy-2-(2-phenylethyl)chromone(isomer) |
| 37.4 | 567.2007 | 0.19105 | 5.64132 | 0.02626 | 4m | THPECs | FTPECs | S-Dimers | S4-1(isomer) |
| 39.64 | 251.1047 | 0.43099 | 4.16711 | 0.02797 | 4m |  |  | FTPECs | 2-(2-phenylethyl)chromone |
| 40.77 | 311.127 | 0.31144 | 12.727 | 0.007411 | 4m | 2OCH_3_ |  | FTPECs | 6,7-dimethoxy-2-(2-phenylethyl)chromone(isomer) |
| 41.95 | 281.1165 | 0.26779 | 20.9419 | 0.0056701 | 4m | OCH_3_ |  | FTPECs | 6-methoxy-2-(2-phenylethyl)chromone(isomer) |
| 45.3 | 297.1111 | 0.35897 | 4.88029 | 0.002751 | 4m | OH | OCH_3_ | FTPECs | 6-hydroxy-2-[2-(4'-methoxyphenyl)ethyl]chromone(isomer) |

**Supplementary Table S13.** Identification information of differential compounds between 6M and 8M.

| **t_R_(min)** | **[M+H]^+^ (*m/z*)** | **FC** | **VIP** | ***p*** | **Trend** | **A ring** | **B ring** | **Type** | **Proposed compound** |
| --- | --- | --- | --- | --- | --- | --- | --- | --- | --- |
| 7.94 | 319.1168 | 2.0892 | 4.63251 | 0.051753 | 8m | 4OH |  | THPECs | Agarotetrol |
| 8.88 | 319.1166 | 1.2319 | 4.36956 | 0.50943 | 8m | 4OH |  | THPECs | Agarotetrol(isomer) |
| 9.33 | 319.1166 | 4.0346 | 2.95531 | 0.017296 | 8m | 4OH |  | THPECs | Agarotetrol(isomer) |
| 12.86 | 329.1016 | 0.74202 | 9.40701 | 0.26921 | 6m | 2-O- | OH, OCH_3_ | DEPECs | Oxidoagarochromone C(isomer) |
| 14.01 | 301.1063 | 3.6165 | 3.77145 | 0.064724 | 8m | -O-,2OH |  | EPECs | 2-(2-phenylethyl)-6,7-epoxy-5,8-dihydroxy-5,6,7,8-tetrahydrochromone(isomer) |
| 16.98 | 337.0829 | 2.5713 | 3.47409 | 0.0071891 | 8m | 3OH,Cl |  | THPECs | 8-chloro-5,6,7-trihydroxy-5,6,7,8-tetrahydro-2-(2-phenylethyl)chromone(isomer) |
| 17.89 | 331.1168 | 1.4156 | 2.33302 | 0.2483 | 8m | -O-,2OH | OCH_3_ | EPECs | 2-[2-(4'-methoxyphenyl)ethyl]-6,7-epoxy-5,8-dihydroxy-5,6,7,8-tetrahydrochromone(isomer) |
| 18.35 | 301.1064 | 2.4524 | 6.91632 | 0.052602 | 8m | -O-,2OH |  | EPECs | 2-(2-phenylethyl)-6,7-epoxy-5,8-dihydroxy-5,6,7,8-tetrahydrochromone(isomer) |
| 22.74 | 283.0968 | 1.3198 | 26.6617 | 0.24275 | 8m | 2-O- |  | DEPECs | Oxidoagarochromone A |
| 27.46 | 297.111 | 1.5449 | 5.22723 | 0.34006 | 8m | OCH_3_ | OH | FTPECs | 6-methoxy-2-[2-(4'-hydroxyphenyl)ethyl]chromone(isomer) |
| 27.55 | 327.1219 | 2.3402 | 4.1956 | 0.033196 | 8m | OCH_3_ | OH,OCH_3_ | FTPECs | 6-methoxy-2-[2-(3'-hydroxy-4'-methoxyphenyl)ethyl]chromone(isomer) |
| 28.44 | 297.1112 | 1.6286 | 2.504 | 0.15702 | 8m | OH,OCH3 |  | FTPECs | 6-hydroxy-7-methoxy-2-(2-phenylethyl)chromone(isomer) |
| 31.29 | 267.1005 | 1.4714 | 6.4704 | 0.29321 | 8m | OH |  | FTPECs | 6-hydroxy-2-(2-phenylethyl)chromone(isomer) |
| 33.3 | 341.1376 | 1.3395 | 16.517 | 0.40871 | 8m | 2 OCH_3_ | OCH_3_ | FTPECs | 6,7-dimethoxy-2-[2-(4'-methoxyphenyl)ethyl]chromone(isomer) |
| 34.41 | 311.1274 | 1.5231 | 4.90254 | 0.084886 | 8m | 2 OCH_3_ |  | FTPECs | 6,7-dimethoxy-2-(2-phenylethyl)chromone(isomer) |
| 36.04 | 567.2011 | 2.013 | 2.89994 | 0.08399 | 8m | THPECs | FTPECs | S-Dimers | S4-1(isomer) |
| 39.55 | 251.1056 | 1.3998 | 5.58505 | 0.37504 | 8m |  |  | FTPECs | 2-(2-phenylethyl)chromone |
| 39.59 | 281.1161 | 1.9455 | 1.45651 | 0.092257 | 8m | OCH_3_ |  | FTPECs | 6-methoxy-2-(2-phenylethyl)chromone(isomer) |
| 40.73 | 311.127 | 1.7308 | 9.91685 | 0.29488 | 8m | OCH_3_ | OCH_3_ | FTPECs | 6-methoxy-2-[2-(4'-methoxyphenyl)ethyl]chromone(isomer) |
| 41.89 | 281.1163 | 1.7827 | 12.0714 | 0.2191 | 8m | OCH_3_ |  | FTPECs | 6-methoxy-2-(2-phenylethyl)chromone(isomer) |
| 45.23 | 297.111 | 1.5458 | 2.20363 | 0.26536 | 8m | OH | OCH_3_ | FTPECs | 6-hydroxy-2-[2-(4'-methoxyphenyl)ethyl]chromone(isomer) |
| 48.88 | 549.1912 | 1.4744 | 3.53946 | 0.40111 | 8m | THPECs | FTPECs | S-Dimers | S3-2(isomer) |

**Supplementary Table S14.** Identification information of differential compounds between 8M and 10M.

| **t_R_(min)** | **[M+H]+ (*m/z*)** | **FC** | **VIP** | **p** | **Trend** | **A ring** | **B ring** | **Type** | **Proposed compound** |
| --- | --- | --- | --- | --- | --- | --- | --- | --- | --- |
| 5.55 | 365.122 | 2.4508 | 4.23354 | 0.077096 | 10m | 4OH | OH,OCH_3_ | THPECs | 2-[2-(3-hydroxy-4-methoxyphenyl)ethyl]-5,6,7,8-tetrahydroxy-5,6,7,8-tetrahydrochromone(isomer) |
| 7.67 | 347.1115 | 4.4619 | 9.70635 | 0.014495 | 10m | -O-,2OH | OH,OCH_3_ | EPECs | 2-[2-(3'-hydroxy-4'-methoxyphenyl)ethyl]-6,7-epoxy-5,8-dihydroxy-5,6,7,8-tetrahydrochromone(isomer) |
| 8.02 | 349.1272 | 1.4744 | 4.82744 | 0.15172 | 10m | 4OH | OCH_3_ | THPECs | 2-[2-(4'-methoxyphenyl)ethyl]-5,6,7,8-tetrahydroxy-5,6,7,8-tetrahydrochromone(isomer) |
| 9.33 | 319.1167 | 1.7484 | 4.93559 | 0.091592 | 10m | 4OH |  | THPECs | Agarotetrol(isomer) |
| 9.99 | 347.1117 | 3.4266 | 13.5029 | 0.032581 | 10m | -O-,2OH | OH,OCH_3_ | EPECs | 2-[2-(3'-hydroxy-4'-methoxyphenyl)ethyl]-6,7-epoxy-5,8-dihydroxy-5,6,7,8-tetrahydrochromone(isomer) |
| 13.73 | 331.1171 | 3.1248 | 5.64498 | 0.018575 | 10m | -O-,2OH | OCH_3_ | EPECs | Oxidoagarochromone B(isomer) |
| 13.97 | 301.1063 | 2.3195 | 8.39096 | 0.17334 | 10m | -O-,2OH | OH | EPECs | 2-[2-(4'-hydroxy)ethyl]-6,7-epoxy-5,8-dihydroxy-5,6,7,8-tetrahydrochromone(isomer) |
| 16.96 | 337.0832 | 0.72352 | 4.81758 | 0.24328 | 8m | Cl,3OH |  | THPECs | 8-chloro-5,6,7-trihydroxy-5,6,7,8-tetrahydro-2-(2-phenylethyl)chromone(isomer) |
| 17.84 | 331.1168 | 2.4087 | 13.241 | 0.018737 | 10m | -O-,2OH | OCH_3_ | EPECs | 2-[2-(4'-methoxyphenyl)ethyl]-6,7-epoxy-5,8-dihydroxy-5,6,7,8-tetrahydrochromone(isomer) |
| 18.3 | 301.1063 | 1.594 | 14.4692 | 0.22995 | 10m | -O-,2OH |  | EPECs | 2-(2-phenylethyl)-6,7-epoxy-5,8-dihydroxy-5,6,7,8-tetrahydrochromone(isomer) |
| 22.65 | 283.1004 | 0.70486 | 4.73473 | 0.30868 | 8m | 2-O- |  | DEPECs | Oxidoagarochromone A |
| 24.16 | 641.2021 | 1.3114 | 6.25997 | 0.44514 | 10m | THPECs | FTPECs | S-Dimers | S1-2(isomer) |
| 26.98 | 611.1921 | 0.66114 | 8.53243 | 0.064519 | 8m | THPECs | FTPECs | S-Dimers | S1-1(isomer) |
| 28.4 | 297.1112 | 1.4736 | 6.52507 | 0.13882 | 10m | OH,OCH3 |  | FTPECs | 6-hydroxy-7-methoxy-2-(2-phenylethyl)chromone(isomer) |
| 34.13 | 595.1960 | 2.1896 | 4.13854 | 0.013962 | 10m | THPECs | FTPECs | S-Dimers | S8-2(isomer) |
| 37.3 | 565.1859 | 0.63243 | 4.72049 | 0.094649 | 8m | THPECs | FTPECs | S-Dimers | S7-1(isomer) |
| 40.62 | 311.1262 | 0.33377 | 3.7994 | 0.019808 | 8m | OCH_3_ | OCH_3_ | FTPECs | 6-methoxy-2-[2-(4'- methoxyphenyl)ethyl)]chromone(isomer) |
| 42.34 | 281.1180 | 0.51912 | 3.6714 | 0.0722 | 8m | OCH_3_ |  | FTPECs | 6-methoxy-2-(2-phenylethyl)chromone(isomer) |
| 46.22 | 549.1907 | 0.54319 | 3.89306 | 0.12443 | 8m | THPECs | FTPECs | S-Dimers | S3-2(isomer) |
| 47.39 | 579.2019 | 0.38784 | 9.95739 | 0.022827 | 8m | THPECs | FTPECs | S-Dimers | S-2(isomer) |
| 48.81 | 549.1912 | 0.44071 | 13.3524 | 0.039268 | 8m | THPECs | FTPECs | S-Dimers | S3-2(isomer) |

**Supplementary Table S15.** Identification information of differential compounds between 10M and 12M.

| **t_R_(min)** | **[M+H]+ (*m/z*)** | **FC** | **VIP** | **p** | **Trend** | **A ring** | **B ring** | **Type** | **Proposed compound** |
| --- | --- | --- | --- | --- | --- | --- | --- | --- | --- |
| 7.69 | 347.1116 | 1.3171 | 5.73231 | 0.45781 | 12m | 4OH | OH, OCH_3_ | THPECS | 2-[2-(3-hydroxy-4-methoxyphenyl)ethyl]-5,6,7,8-tetrahydroxy-5,6,7,8-tetrahydrochromone(isomer) |
| 8.00 | 349.1273 | 1.4616 | 4.4058 | 0.25616 | 12m | 4OH | OCH_3_ | THPECS | 2-[2-(4'-methoxyphenyl)ethyl]-5,6,7,8-tetrahydroxy-5,6,7,8-tetrahydrochromone(isomer) |
| 9.34 | 349.1273 | 1.7508 | 4.35237 | 0.18719 | 12m | 4OH | OCH_3_ | THPECS | 2-[2-(4'-methoxyphenyl)ethyl]-5,6,7,8-tetrahydroxy-5,6,7,8-tetrahydrochromone(isomer) |
| 11.22 | 303.1217 | 2.2994 | 5.37789 | 0.068343 | 12m | 3OH |  | THPECS | 5,6,7-trihydroxy-5,6,7,8-tetrahydro-2-(2-phenylethyl)chromone(isomer) |
| 14.01 | 301.1062 | 1.5303 | 12.1273 | 0.34227 | 12m | -O-,2OH | OH | EPECs | 2-[2-(4'-hydroxy)ethyl]-6,7-epoxy-5,8-dihydroxy-5,6,7,8-tetrahydrochromone(isomer) |
| 17.89 | 331.1169 | 1.4563 | 11.8007 | 0.26625 | 12m | -O-,2OH | OCH_3_ | EPECs | 2-[2-(4'-methoxyphenyl)ethyl]-6,7-epoxy-5,8-dihydroxy-5,6,7,8-tetrahydrochromone(isomer) |
| 19.05 | 657.1983 | 0.50278 | 7.28449 | 0.21603 | 10m |  |  |  | - |
| 21.52 | 283.0955 | 1.9849 | 9.67284 | 0.093345 | 12m | 2-O- |  | DEPECs | Oxidoagarochromone A(isomer) |
| 21.95 | 357.1324 | 1.5292 | 3.59668 | 0.26099 | 12m | OCH_3_ | OH,OCH_3_ | FTPECs | 6,7-dimethoxy-2-[2-(3'-hydroxy-4'-methoxyphenyl)ethyl]chromone(isomer) |
| 22.72 | 283.0964 | 0.70324 | 30.3297 | 0.27897 | 10m | 2-O- |  | DEPECs | Oxidoagarochromone A(isomer) |
| 22.98 | 357.1325 | 1.7047 | 7.62225 | 0.19644 | 12m | OCH_3_ | OH,OCH_3_ | FTPECs | 6,7-dimethoxy-2-[2-(3'-hydroxy-4'-methoxyphenyl)ethyl]chromone(isomer) |
| 23.38 | 313.1063 | 1.9453 | 8.32158 | 0.16794 | 12m | 2-O- | OCH_3_ | DEPECs | Oxidoagarochromone B(isomer) |
| 24.21 | 283.0955 | 2.0413 | 10.7668 | 0.099006 | 12m | 2-O- |  | DEPECs | Oxidoagarochromone A(isomer) |
| 25.04 | 267.1004 | 0.55386 | 3.68855 | 0.12975 | 10m |  | OH | FTPECs | 2-[2-(4'-hydroxy phenyl)ethyl]chromone(isomer) |
| 26.33 | 641.2028 | 0.65814 | 4.9653 | 0.27841 | 10m | THPECs | FTPECs | S-Dimers | S1-2(isomer) |
| 27.81 | 327.1219 | 1.6247 | 15.6634 | 0.23617 | 12m | OCH_3_ | OH,OCH_3_ | FTPECs | 6-methoxy-2-[2-(3'-hydroxy-4'-methoxyphenyl)ethyl]chromone(isomer) |
| 28.87 | 327.1218 | 2.8732 | 11.1664 | 0.10232 | 12m | OCH_3_ | OH,OCH_3_ | FTPECs | 6-methoxy-2-[2-(3'-hydroxy-4'-methoxyphenyl)ethyl]chromone(isomer) |
| 29.69 | 611.1925 | 0.66917 | 4.82864 | 0.26297 | 10m | THPECs | FTPECs | S-Dimers | S1-1(isomer) |
| 32.84 | 595.1973 | 0.74414 | 4.49521 | 0.27212 | 10m | THPECs | FTPECs | S-Dimers | S8-2(isomer) |
| 32.97 | 639.2224 | 2.0289 | 3.86557 | 0.22082 | 12m | THPECs | FTPECs | S-Dimers | S6-1(isomer) |
| 33.28 | 341.1377 | 1.4044 | 15.7848 | 0.29343 | 12m | 2OCH_3_ | OCH_3_ | FTPECs | 6,7-dimethoxy-2-[2-(4'-methoxyphenyl)ethyl]chromone(isomer) |
| 34.76 | 565.244 | 1.9875 | 4.65521 | 0.36922 | 10m | THPECs | FTPECs | S-Dimers | S7-1(isomer) |
| 36.05 | 567.2009 | 0.37572 | 5.34975 | 0.11411 | 10m | THPECs | FTPECs | D-Dimers | S4-1(isomer) |
| 38.24 | 281.116 | 0.62509 | 4.217 | 0.085396 | 10m |  | OCH_3_ | FTPECs | 2-[2-(4'-methoxyphenyl)ethyl]chromone(isomer) |
| 39.54 | 251.1056 | 0.46006 | 14.4906 | 0.17156 | 10m |  |  | FTPECs | 2-(2-phenylethyl)chromone |
| 40.68 | 311.127 | 1.9263 | 13.632 | 0.12674 | 12m | OCH_3_ | OCH_3_ | FTPECs | 6-methoxy-2-[2-(4'-methoxyphenyl)ethyl]chromone(isomer) |
| 42.11 | 281.1145 | 1.0854 | 4.85482 | 0.71563 | 12m | OCH_3_ |  | FTPECs | 6-methoxy-2-(2-phenylethyl)chromone(isomer) |
| 43.62 | 327.1217 | 1.6623 | 4.5968 | 0.12828 | 12m | OH,OCH_3_ | OCH_3_ | FTPECs | 6-hydroxy-7-methoxy-2-[2-(4'-methoxyphenyl)ethyl]chromone(isomer) |
| 47.4 | 579.2017 | 0.40596 | 6.57923 | 0.036706 | 10m | THPECs | THPECs | S-Dimers | S-2(isomer) |
| 48.84 | 549.1912 | 0.57711 | 8.07834 | 0.20085 | 10m | THPECs | THPECs | S-Dimers | S3-2(isomer) |

# Information of 58 Differentiated Compounds.

**Supplementary Table S16.** Table of Information on 58 Differentiated Compounds.

|  | **[M+H]^+^ (*m/z*)** | **Proposed compound** |
| --- | --- | --- |
| ① | 361.1261 | 2-[2-(3'-hydroxy-4'-methoxyphenyl)ethyl]-7,8-epoxy-5-methoxy-6-hydroxy-5,6,7,8-tetrahydrochromone(isomer) |
|  | 565.1859 | S7-1(isomer) |
|  | 549.1907 | S3-2(isomer) |
|  | 549.1912 | S3-2(isomer)* |
| ② | 283.0796 | Oxidoagarochromone A* |
|  | 281.1160 | 2-[2-(4'-methoxyphenyl)ethyl]chromone(isomer) |
|  | 579.2017 | S-2(isomer)* |
| ③ | 267.1005 | 6-hydroxy-2-(2-phenylethyl)chromone(isomer) |
|  | 281.1165 | 6-methoxy-2-(2-phenylethyl)chromone(isomer)* |
|  | 297.1111 | 6-hydroxy-7-methoxy-2-(2-phenylethyl)chromone(isomer)* |
|  | 311.127 | 6-methoxy-2-[2-(4'-methoxyphenyl)ethyl]chromone(isomer)* |
|  | 319.1166 | 2-(2-phenylethyl)-5,6,7,8-tetrahydroxy-5,6,7,8-tetrahydrochromone(isomer)* |
|  | 281.1161 | 6-methoxy-2-(2-phenylethyl)chromone(isomer) |
| ④ | 283.0796 | Oxidoagarochromone A* |
|  | 251.1047 | 2-(2-phenylethyl)chromone* |
|  | 567.2007 | D1-4(isomer)* |
|  | 567.2009 | S4-1(isomer) |
| ⑤ | 299.089 | 5,6:7,8-diepoxy-2-[2-(4-hydroxy)ethyl]-5,6,7,8-tetrahydrochromone(isomer) |
|  | 329.1016 | Oxidoagarochromone C(isomer)* |
|  | 657.1990 | D-14(isomer) |
|  | 641.2021 | S1-2(isomer) |
|  | 641.2027 | S1-2(isomer)* |
|  | 611.1921 | S1-1(isomer) |
|  | 641.2024 | S1-2(isomer)* |
|  | 611.1925 | S1-1(isomer) |
|  | 311.1278 | 6,7-dimethoxy-2-(2-phenylethyl)chromone(isomer)* |
| ⑥ | 319.1167 | Agarotetrol* |
|  | 565.2440 | S7-1(isomer) |
|  | 549.2479 | S3-2(isomer) |
|  | 639.2224 | S6-1(isomer) |
|  | 313.1076 | 5,6:7,8-diepoxy-2-[2-(4-methoxy)ethyl]-5,6,7,8-tetrahydrochromone(isomer) |
|  | 327.1219 | 6-methoxy-2-[2-(3'-hydroxy-4'-methoxyphenyl)ethyl]chromone(isomer) |
| ⑦ | 349.1272 | 2-[2-(4'-methoxyphenyl)ethyl]-5,6,7,8-tetrahydroxy-5,6,7,8-tetrahydrochromone(isomer) |
|  | 349.1273 | Agarotetrol(isomer) |
|  | 313.1063 | Oxidoagarochromone B(isomer) |
|  | 331.1171 | 2-[2-(4'-methoxyphenyl)ethyl]-6,7-epoxy-5,8-dihydroxy-5,6,7,8-tetrahydrochromone(isomer)* |
|  | 331.1168 | 2-[2-(4'-methoxyphenyl)ethyl]-6,7-epoxy-5,8-dihydroxy-5,6,7,8-tetrahydrochromone(isomer)* |
| ⑧ | 301.1064 | 2-(2-phenylethyl)-6,7-epoxy-5,8-dihydroxy-5,6,7,8-tetrahydrochromone(isomer)* |
|  | 303.1217 | 5,6,7-trihydroxy-5,6,7,8-tetrahydro-2-(2-phenylethyl)chromone(isomer) |
|  | 283.0955 | Oxidoagarochromone A(isomer) |
|  | 595.196 | S8-2(isomer)* |
|  | 301.1062 | 2-(2-phenylethyl)-6,7-epoxy-5,8-dihydroxy-5,6,7,8-tetrahydrochromone(isomer)* |
|  | 319.1166 | 2-(2-phenylethyl)-5,6,7,8-tetrahydroxy-5,6,7,8-tetrahydrochromone(isomer)* |
|  | 283.0955 | Oxidoagarochromone A(isomer) |
| ⑨ | 327.1212 | 6-methoxy-2-[2-(3'-hydroxy-4'-methoxyphenyl)ethyl]chromone(isomer)* |
|  | 347.1117 | 2-[2-(3'-hydroxy-4'-methoxyphenyl)ethyl]-6,7-epoxy-5,8-dihydroxy-5,6,7,8-tetrahydrochromone(isomer)* |
|  | 365.122 | 2-[2-(3-hydroxy-4-methoxyphenyl)ethyl]-5,6,7,8-tetrahydroxy-5,6,7,8-tetrahydrochromone(isomer) |
|  | 357.1324 | 6,7-dimethoxy-2-[2-(3'-hydroxy-4'-methoxyphenyl)ethyl]chromone(isomer) |
|  | 357.1325 | 6,7-dimethoxy-2-[2-(3'-hydroxy-4'-methoxyphenyl)ethyl]chromone(isomer) |
|  | 347.1115 | 2-[2-(3'-hydroxy-4'-methoxyphenyl)ethyl]-6,7-epoxy-5,8-dihydroxy-5,6,7,8-tetrahydrochromone(isomer) * |
|  | 327.1218 | 6,7-dimethoxy-2-[2-(4'-methoxyphenyl)ethyl]chromone(isomer) |
| ⑩ | 297.1110 | 6-methoxy-2-[2-(4'-hydroxyphenyl)ethyl]chromone(isomer) |
|  | 297.1112 | 6-hydroxy-7-methoxy-2-(2-phenylethyl)chromone(isomer) |
|  | 341.1377 | 6,7-dimethoxy-2-[2-(4'-methoxyphenyl)ethyl]chromone(isomer) |
|  | 327.1217 | 6-hydroxy-7-methoxy-2-[2-(4'-methoxyphenyl)ethyl]chromone(isomer) |
|  | 337.0829 | 8-chloro-5,6,7-trihydroxy-5,6,7,8-tetrahydro-2-(2-phenylethyl)chromone(isomer)* |
|  | 595.1973 | S8-2(isomer) |
|  | 267.1004 | 2-[2-(4'-hydroxy phenyl)ethyl]chromone(isomer) |

Note: * denotes significant difference (P<0.05)
